# Novel Pyrrolidine-Based Pyrazolines as α‑Glucosidase Inhibitors: Microwave-Assisted Synthesis, Antidiabetic Activity, *In Silico* ADMET Prediction, Molecular Docking, and Molecular Dynamics Simulations

**DOI:** 10.1021/acsomega.5c05455

**Published:** 2025-09-22

**Authors:** Bedriye Seda Kurşun Aktar, Yusuf Sıcak, Abdulkadir Bakırdöven, Gizem Tatar Yılmaz, Süleyman Kaya, Ayşegül Karaküçük-İyidoğan, Emine Elçin Oruç-Emre

**Affiliations:** † Department of Hair Care and Beauty Services, Yeşilyurt Vocational School, 531771 Malatya Turgut Özal University, Battalgazi, Malatya 44210, Türkiye; ‡ Department of Medicinal and Aromatic Plants, Köyceğiz Vocational School, 52986 Mugla Sitki Kocman University, Köyceğiz, Muğla 48000, Türkiye; § Department of Biostatistics and Medical Informatics, Faculty of Medicine, 52976 Karadeniz Technical University, Trabzon 61080, Türkiye; ∥ Department of Bioinformatics, Institute of Health Sciences, 52976 Karadeniz Technical University, Trabzon 61080, Türkiye; ⊥ Yılmaz Bilişim R&D Consulting Software Engineering and Services Trade Limited Company, Trabzon 61081, Türkiye; # Department of Chemistry, Faculty of Arts and Sciences, 37512 Gaziantep University, Gaziantep 27310, Türkiye

## Abstract

Due to their unique properties, small multitargeted drugs containing a fluorinated aromatic moiety and nitrogenous heterocycles are widely available on the market. Considering the pharmacological significance of organofluorine and heterocyclic compounds, in this study, we synthesized a series of pyrazoline derivatives (**14–27**) containing a pyrrolidine moiety and substituted them with a fluorine atom or a fluorine-containing (−CF_3_ or -OCF_3_) group at different positions. Also, the antidiabetic activities of new pyrazolines were screened by in vitro α-glucosidase and α-amylase activity assays in order to investigate their potential use in the treatment of *Diabetes Mellitus*, one of the most common and rapidly spreading diseases of today. The findings of this research indicated that compound **21,** having a trifluoromethoxy group at the *ortho* position of the pyrrolidine-based pyrazolines at the phenyl ring, was determined to be the most effective α-glucosidase inhibitor with IC_50_ values of 52.79 ± 6.00 μM, compared to acarbose (IC_50_: 121.65 ± 0.50 μM). Molecular modeling studies demonstrated the high specificity of the most active pyrazoline–pyrrolidine hybrid molecules to the active site of α-glucosidase and their potential to exert inhibitory effects through various interactions with basic residues. Furthermore, molecular dynamics simulations provided comprehensive information about the structural properties and binding mechanisms of the complexes.

## Graphical abstract

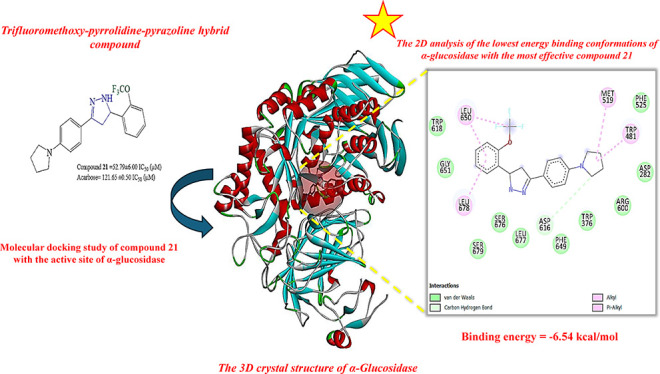

## 1 Introduction

*Diabetes mellitus* (DM), which is categorized as Type I and Type II, is a metabolic disorder resulting from the abnormal metabolism of carbohydrates, lipids, and proteins, which occurs due to the relative or absolute deficiency of insulin secretion, resistance to insulin action in body tissues or both.^1–3^ The International Diabetes Federation (IDF), which currently reports that 589 million adults (ages 20–79) are living with diabetes, predicts that the number of people with diabetes worldwide could reach 853 million by 2050. In addition, according to the Diabetes Atlas Global Report (2024), it is reported that diabetes-related deaths reached 3.4 million in 2024, and there has been a 338% increase in healthcare costs over the last 17 years.^4^ In particular, type II DM (T2DM), which is caused by an imbalance between the amount of insulin released and consumed, is being researched more because it is more frequently diagnosed and preventable.^5^ The contribution of risk factors such as age and obesity to T2DM, which develops due to impaired glucose metabolism known to be linked to dietary changes, lifestyle changes, stress, environmental factors, and lack of physical activity, the presence of hypertension, and some hereditary predispositions, is undeniable.^6^

The most preferred approach to treat T2DM is to control blood glucose levels by reducing postprandial hyperglycemia, which is characterized by elevated blood sugar levels due to suboptimal insulin production or inadequate cellular response.^7^ α-Glucosidase and α-amylase, which regulate postprandial glucose levels by controlling carbohydrate hydrolysis, are the main enzymes that attract the attention of researchers in studies on the treatment of diabetes.^8–11^ α-Amylase, a protein found in the salivary glands and pancreas, catalyzes the breakdown of polysaccharides such as starch and glycogen into oligosaccharides and disaccharides by acting on α-1,4-glycosidic bonds.^12^ Another member of glycoside hydrolases, α-glucosidase, an enzyme usually secreted from the small intestinal epithelium, is involved in the conversion of oligosaccharides and disaccharides to glucose during food digestion. As a result, the glucose produced is absorbed into the bloodstream, increasing postprandial blood glucose levels. When α-glucosidase is inhibited, it delays the production of glucose by hydrolysis of α-(1–4)-linked *D*-glucose residues from the nonreducing end of α-glucoside and controls type II DM by reducing the postprandial blood glucose increase.^13^ In this context, targeting α-amylase and α-glucosidase enzymes would prevent the excessive production of assimilable glucose and provide an effective approach to maintain normoglycemia in type II diabetes. Currently, clinically approved α-glucosidase inhibitors such as acarbose, miglitol, and voglibose are used in the treatment of diabetes to manage blood glucose levels. Acarbose, the most prescribed oral drug, also inhibits the action of α-amylase. However, due to its higher inhibition of salivary and pancreatic α-amylase compared to α-glucosidase, it has been found that most of its serious side effects, such as abdominal pain, gas, bloating, and diarrhea, are due to the accumulation of undigested carbohydrates in the colon.^14^ Therefore, there is an urgent need to find newer and safer selective inhibitors of α-glucosidase that can modulate T2DM.^15^

The presence of organofluorine compounds in the structure of antidiabetic drugs currently used in treatment, such as sitagliptin, has led to the addition of substituents carrying fluorine atoms to the skeletons of designed compounds in studies to discover potential antidiabetic agents.^16^ In addition, it has been determined that substituents carrying fluorine atoms in various positions are selective in increasing α-glucosidase activity and interact with amino acid residues in the active site of the enzyme by hydrogen bonding.^17,18^ Organofluorine compounds are used in various applications in material science such as biomaterials, smart materials, liquid crystal displays, fuel cells, and solar cells due to their unique physicochemical and chemical reactivity features, as well as in agrochemicals and components of many drugs due to their optimal membrane permeability and enhanced bioavailability properties.^19^ The carbon–fluorine bond is known to be relatively stable against chemical or metabolic transformations due to its notably strong bond strength and oxidative stability.^20^ Also, due to the high electronegativity of the fluorine atom and its significantly lower hydrogen bonding activity than oxygen or nitrogen atoms, it has been determined that, in many cases, the hydrophobic interactions of carbon–fluorine bonds rather than C–F···H hydrogen bond interactions play a dominant role in the stabilization of enzyme–substrate complexes.^21^ In contrast, it has been reported that close amide-NH···F and C–F···C=O interactions between fluorine and amide residues in proteins or enzymes are very common and markedly influence protein–ligand interactions, with a resulting considerable increase in binding affinities.^22–24^ Therefore, their importance in drug discovery is well-known, and today, a large number of drug candidate molecules contain one or more fluorine atoms (e.g., CF, CF_2_, and CF_3_). Moreover, approximately one-third of the best-selling drugs (Figure 1) currently marketed are fluorinated molecules.^25,26^ The approach of adding fluorine groups to lead compounds at various positions is systematically applied in drug discovery studies to optimize multiple properties ranging from superior receptor affinity to improved absorption, distribution, metabolism, and excretion (ADME) profile.^27^

**Figure 1:**
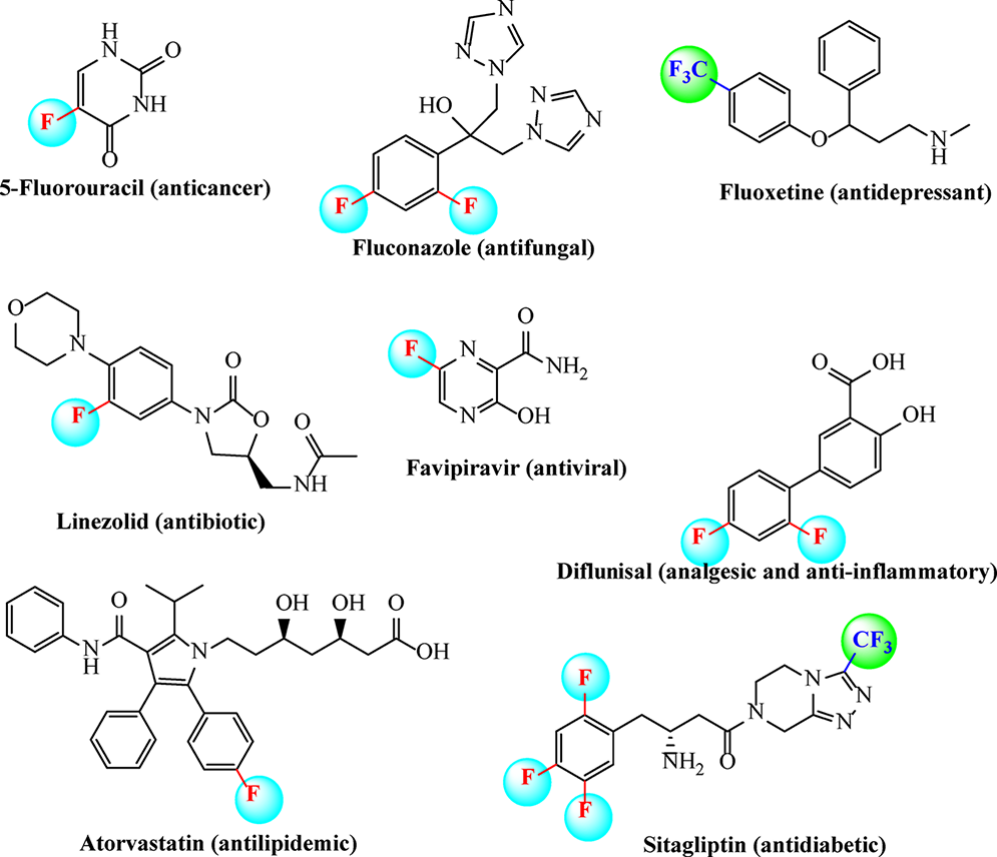
Some marketed drugs containing an organofluorine group.

On the other hand, various nitrogen-containing heterocyclic compounds that have been found in nature exhibit pharmacological and physiological properties and are main constituents of numerous bioactive and vital molecules, such as proteins, nucleic acids, and enzymes.^28^ Therefore, studies focusing on the design and discovery of drug candidate molecules have reported that a nitrogen atom should be present in the structural framework.^29,30^ In cases of abnormally high enzyme activity leading to certain health problems and diseases, it has been shown that heterocyclic compounds containing at least one nitrogen atom in their structures provide significant contributions to pharmacological activities through target enzyme inhibition.^31–33^ Among the heterocyclics, pyrazoline derivatives (Figure 2), which have attracted great attention due to their nitrogenous structural cores, are desirable chemical scaffolds in the discovery of pharmacologically active compounds due to their anticancer, anti-inflammatory, antidiabetic, antifungal, antithrombotic, antiviral, analgesic, antihypertensive, and antidepressant activities.^34,35^

**Figure 2:**
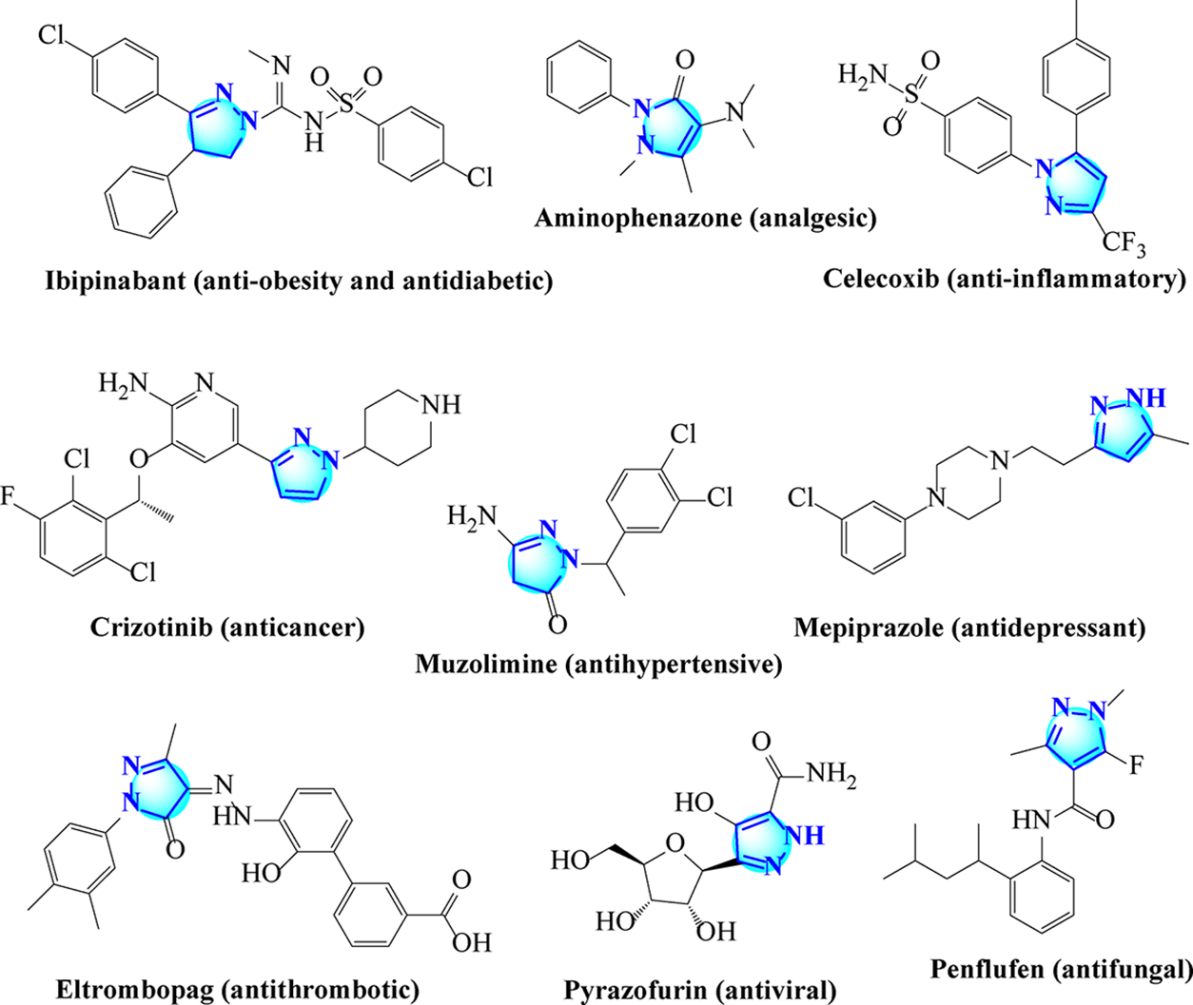
Pyrazoline-based drugs.

In the context of research into the discovery of high-potential agents and drug development studies that can be used in the treatment of diabetes, building blocks such as pyrrolidine and pyrazoline have considerable importance. Pyrrolidine derivatives, one of the nitrogenous heterocycles, have a wide range of biological activities and are the most common heterocyclic moieties in drugs due to their structural versatility and prevalence in natural products.^36^ In addition, the structural characteristics of the pyrrolidine skeleton, which have also attracted attention as potential therapeutic agents in the treatment of DM, can improve their inhibitory activity by affecting the ability of the molecules to fit into the active sites of target enzymes.^37^ Moreover, the integration of variable pharmacophores, notably pyrazoline, within pyrrolidine-based compounds provides a framework for the exploration of structure–activity relationships (SARs) and facilitates the rational design of more potent and selective inhibitors, enhancing therapeutic efficacy in the management of diabetes.^12,38^

Inspired by the therapeutic importance of organofluorine compounds, pyrazoline, and pyrrolidine patterns and as a continuation of our research on identifying new α-glucosidase and α-amylase inhibitors, in this study, we aimed to synthesize pyrazoline derivatives with fluorine substituents at different positions in order to evaluate their potential as α-glucosidase and α-amylase enzyme inhibitors. Therefore, chalcone derivatives carrying fluorine atoms at different positions and types were used as precursors in the study to obtain pyrazolines with an electron-rich and biologically active core.^39^ Briefly, the design strategy of this study is based on the idea that the dimensions, form, and flexibility of pyrazoline scaffolds formed by cyclization of chalcone derivatives can affect the conformation of molecules to the active sites of target enzymes and thus enhance their inhibitory activities. In our previous work, various chalcone analogues of 4-(pyrrolidin-1-yl)­acetophenone were synthesized by reaction with the benzaldehydes containing fluorine groups (F, CF_3,_ and OCF_3_) at the various positions, and their antidiabetic activity was tested.^37^ In the present study, a series of new pyrazoline derivatives containing a pyrrolidine moiety for the first time was obtained by the cyclization of pyrrolidine-based chalcone derivatives prepared in our previous study with hydrazine monohydrate. All pyrazoline derivatives were subjected to in vitro evaluation to determine their inhibitory effects on α-glucosidase and α-amylase enzymes. Additionally, *in silico* studies were performed to gain insights into their interactions with the active pockets of α-glucosidase and α-amylase enzymes.

## 2 Experimental Section

### 2.1 Materials and Methods

The chemicals and solvents used in this study were of analytical grade, procured from Acros, Alfa Aesar, Sigma-Aldrich, and Merck. All reactions with microwave irradiation were accomplished using a Milestone StartSYNTH Microwave Labstation under reflux. Monitoring of chemical reaction was performed using thin-layer chromatography (TLC, Merck 60 F_254_). An SMP20 instrument was used to determine the melting points of compounds that were uncorrected. FT-IR spectra of the compounds were obtained using a PerkinElmer 1620 model FT-IR spectrophotometer, covering the wavelength range from 4000 to 400 cm^–1^. ^1^H- and ^13^C NMR spectra were recorded on a Bruker Avance III HD 600 MHz NMR. Elemental analyses (CHNS) were performed on a Thermo Scientific Flash 2000 Organic elemental analyzer. The Agilent Technologies 1260 Infinity II LC-MS/MS 6460 Triple Quad Mass Spectrometer device was used to perform the mass analyses by the ionization method. The α-glucosidase and α-amylase inhibitory activities were assessed using a 96-well microplate reader, SpectraMax 340PC^384^, Molecular Devices (USA). Spectroscopic data of compounds **14–28** are given in the Supporting Information.

### 2.2 General Procedure for the Synthesis of Pyrazolines **(14–27)**

Novel pyrrolidine-based pyrazolines **(14–27)** derivatives were obtained from cyclization products according to the literature method^40,41^ of (*E*)-3-[substituted phenyl]-1-[4-(pyrrolidin-1-yl)­phenyl]­prop-2-en-1-one **1–13** ^37^ derivatives, previously reported by my research group. For the details of the synthesis procedure, see the Supporting Information.

#### 2.2.1 5-Phenyl-3-[4-(pyrrolidin-1-yl)­phenyl]-4,5-dihydro-1*H*-pyrazole **(14)**

Brown solid, yield: 36%, m.p: 140–142 °C. **FTIR ν_max_ (cm** ^**–1**^ **):** 1484, 1594, 1606 (C=C); 2964, 3028 (C–H); 3273 (N–H). ^**1**^ **H NMR (600 MHz) (DMSO-** ***d***_**6**_ **/TMS) δ ppm:** 1.79 (t, 4H, *J*_1_,_2_ = 6.6 Hz, protons of the pyrrolidine ring); 2.57–2.61 (dd, 1H, *J*_1_,_2_ = 10.8 Hz, pyrazoline ring CH_2_); 3.08 (t, 4H, *J*_1_ = 7.2 Hz, *J*_2_ = 6.6 Hz, protons of the pyrrolidine ring); 3.14 (s, 1H); 3.19–3.24 (dd, 1H, *J*_1_ = 10.2 Hz, *J*_2_ = 10.8 Hz, pyrazoline ring CH_2_); 4.58 (t, 1H, *J*_1_ = 10.8 Hz, *J*_2_ = 10.2 Hz, pyrazoline ring CH); 6.36 (d, 2H, *J* = 8.4 Hz); 7.09 (t, 1H, *J*_1_,_2_ = 7.2 Hz); 7.17 (t, 2H, *J*_1_ = 7.8 Hz, *J*_2_ *=* 7.2 Hz); 7.22 (d, 2H, *J* = 9.0 Hz); 7.29 (d, 2H, *J* = 8.4 Hz). ^**13**^ **C NMR (151 MHz) (DMSO-** ***d***_**6**_ **/TMS) δ ppm:** 25.4, 45.4, 47.7, 47.8, 111.7, 125.5, 126.4, 127.3, 128.5, 129.2, 130.2, 132.6, 151.5. Anal. Calcd for C_19_H_21_N_3_ (291.40 g/mol): C, 78.32; H, 7.26; N, 14.42%. Found: C, 78.46; H, 7.30; N, 14.49%. LC-MS (*m*/*z*): 292.10 [M + H]^+^.

#### 2.2.2 5-(2-Fluorophenyl)-3-[4-(pyrrolidin-1-yl)­phenyl]-4,5-dihydro-1*H*-pyrazole **(15)**

Light brown solid, yield: 48%, m.p: 143–145 °C. **FTIR ν_max_ (cm** ^**–1**^ **):** 1226 (C–F); 1455, 1485, 1524, 1593 (C=C); 2844, 2967 (C–H); 3196 (N–H). ^**1**^ **H NMR (600 MHz) (DMSO-** ***d***_**6**_ **/TMS) δ ppm:** 1.95 (t, 4H, *J*_1,2_ = 6.6 Hz, protons of the pyrrolidine ring); 2.74–2.78 (dd, 1H, *J*_1,2_ = 10.2 Hz, pyrazoline ring CH_2_); 3.31 (s, 1H); 3.36 (4H, protons of the pyrrolidine ring); 3.40–3.44 (dd, 1H, *J*_1,2_ = 10.2 Hz, pyrazoline ring CH_2_); 4.95 (t, 1H, *J*_1_ = 10.8, *J*_2_ = 10.2 Hz, pyrazoline ring CH); 6.52 (d, 2H, *J* = 9.0 Hz); 7.18 (d, 1H, *J* = 7.8 Hz); 7.20 (d, 1H, *J* = 3.6 Hz); 7.31 (t, 1H, *J*_1_ = 8.4, *J*_2_ 5.4 Hz); 7.45 (d, 2H, *J* = 9.0 Hz); 7.50 (t, 1H, *J*_1_ = 6.6, *J*_2_ = 9.6 Hz). ^**13**^ **C NMR (151 MHz) (DMSO-** ***d***_***6***_ **/TMS) δ ppm:** 25.4, 46.2, 47.7, 47.8, 111.2, 111.9, 117.2, 125.4, 126.7, 130.1, 130.8, 131.4, 134.1, 151.2, 151.6. Anal. Calcd for C_19_H_20_FN_3_ (309.39 g/mol): C, 73.76; H, 6.52; N, 13.58%. Found: C, 73.81; H, 6.59; N, 13.63%. LC-MS (*m*/*z*): 310.10 [M + H]^+^.

#### 2.2.3 5-(3-Fluorophenyl)-3-[4-(pyrrolidin-1-yl)­phenyl]-4,5-dihydro-1*H*-pyrazole **(16)**

Brown solid, yield: 49%, m.p: 158–160 °C. **FTIR ν_max_ (cm** ^**–1**^ **):** 1227 (C–F); 1485, 1526, 1594, 1609 (C=C); 2965, 3049 (C–H); 3300 (N–H). ^**1**^ **H NMR (600 MHz) (DMSO-** ***d***_**6**_ **/TMS) δ ppm:** 1.94 (t, 4H, *J*_1,2_ = 6.6 Hz, protons of the pyrrolidine ring); 2.74–2.78 (dd, 1H, *J*_1,2_ = 10.8 Hz, pyrazoline ring CH_2_); 3.23 (t, 4H, *J*_1,2_ = 6.6 Hz, protons of the pyrrolidine ring); 3.35 (s, 1H); 3.37–3.42 (dd, 1H, *J*_1,2_ = 10.2 Hz, pyrazoline ring CH_2_); 4.77 (t, 1H, *J*_1_ = 10.8, *J*_2_ *=* 10.2 Hz, pyrazoline ring CH); 6.52 (d, 2H, *J* = 9.0 Hz); 6.59–6.63 (dd, 1H, *J*_1_ = 9.0, *J*_2_ = 8.4 Hz); 7.09 (t, 1H, *J*_1_ = 8.4, *J*_2_ = 6.0 Hz); 7.36–7.40 (dd, 1H, *J*_1_ = 7.8, *J*_2_ = 6.0 Hz); 7.45 (d, 2H, *J* = 9.0 Hz); 7.82 (t, 1H, *J*_1_= 9.0, *J*_2_ = 7.2 Hz). ^**13**^ **C NMR (151 MHz) (DMSO-** ***d***_**6**_ **/TMS) δ ppm:** 25.4, 45.4, 47.7, 47.8, 111.2, 112.1, 122., 126.63, 127.4, 130.6, 130.9, 131.5, 140.5, 151.2, 151.6. Anal. Calcd for C_19_H_20_FN_3_ (309.39 g/mol): C, 73.76; H, 6.52; N, 13.58%. Found: C, 73.84; H, 6.62; N, 13.65%. LC-MS (*m*/*z*): 310.10 [M + H]^+^.

#### 2.2.4 5-(4-Fluorophenyl)-3-[4-(pyrrolidin-1-yl)­phenyl]-4,5-dihydro-1*H*-pyrazole **(17)**

Yellow solid, yield: 52%, m.p: 95–96 °C. **FTIR ν_max_ (cm** ^**–1**^ **):** 1220 (C–F); 1485, 1525, 1609 (C=C); 2967 (C–H); 3361 (N–H). ^**1**^ **H NMR (600 MHz) (DMSO-** ***d***_**6**_ **/TMS) δ ppm:** 1.96 (t, 4H, *J*_1_ = 7.2, *J*_2_ = 6.6 Hz, protons of the pyrrolidine ring); 2.71–2.76 (dd, 1H, *J*_1_ = 10.8, *J*_2_ = 11.4 Hz, pyrazoline ring CH_2_); 3.25 (t, 4H, *J*_1_ = 7.2, *J*_2_ = 6.6 Hz, protons of the pyrrolidine ring); 3.36 (DMSO water peak and pyrazoline ring CH_2_ (1H), NH (1H)); 4.75 (t, 1H, *J*_1_ = 10.2, *J*_2_ = 10.8 Hz, pyrazoline ring CH); 6.53 (d, 2H, *J* = 9.0 Hz); 7.16 (t, 2H, *J*_1,2_ = 9.0 Hz); 7.41–7.43 (dd, 2H, *J*_1_ = 6.0, *J*_2_ = 5.4 Hz); 7.45 (d, 2H, *J* = 9.0 Hz). ^**13**^ **C NMR (151 MHz) (DMSO-** ***d***_**6**_ **/TMS) δ ppm:** 25.4, 45.4, 47.7, 47.8, 111.2, 111.7, 116.1, 116.3, 126.6, 130.0, 130.2, 138., 151.5. Anal. Calcd for C_19_H_20_FN_3_ (309.39 g/mol): C, 73.76; H, 6.52; N, 13.58%. Found: C, 73.82; H, 6.57; N, 13.66%. LC-MS (*m*/*z*): 310.90 [M + H]^+^.

#### 2.2.5 3-[4-(Pyrrolidin-1-yl)­phenyl]-5-[2-(trifluoromethyl)­phenyl]-4,5-dihydro-1*H*-pyrazole **(18)**

Light brown solid, yield: 49%, m.p: 159–161 °C. **FTIR ν_max_ (cm** ^**–1**^ **):** 1227 (C–F); 1485, 1526, 1594, 1609 (C=C); 2850, 2963, 3049 (C–H); 3346 (N–H). ^**1**^ **H NMR (600 MHz) (DMSO-** ***d***_**6**_ **/TMS) δ ppm:** 1.94 (t, 4H, *J*_1_ = 18.0, *J*_2_ = 21.6 Hz, protons of the pyrrolidine ring); 2.73–2.79 (dd, 1H, *J*_1_ = 16.2, *J*_2_ = 10.8 Hz, pyrazoline ring CH_2_); 3.23 (t, 4H, *J*_1_ = 7.2, *J*_2_ = 6.0 Hz, protons of the pyrrolidine ring); 3.31 (s, 1H); 3.40–3.43 (dd, 1H, *J*_1_ = 4.8, *J*_2_ = 5.4 Hz, pyrazoline ring CH_2_); 5.04 (t, 1H, *J*_1_ = 10.2, *J*_2_ = 10.8 Hz, pyrazoline ring CH); 6.54 (d, 2H, *J* = 9.0 Hz); 7.45 (d, 2H, *J* = 6.6 Hz); 7.67 (d, 1H, *J* = 7.8 Hz); 7.71 (d, 1H, *J* = 12.0 Hz); 7.82 (d, 2H, *J* = 9.0 Hz). ^**13**^ **C NMR (151 MHz) (DMSO-** ***d***_**6**_ **/TMS) δ ppm:** 25.4, 42.9, 47.4, 47.7, 111.2, 111.8, 124.7, 127.3, 128.9, 130.8, 131.6, 133.1, 133.4, 145.4, 148., 151.2. Anal. Calcd for C_20_H_20_F_3_N_3_ (359.40 g/mol): C, 66.84; H, 5.61; N, 11.69%. Found: C, 66.86; H, 5.67; N, 11.72%. LC-MS (*m*/*z*): 360.10 [M + H]^+^.

#### 2.2.6 3-[4-(Pyrrolidin-1-yl)­phenyl]-5-[3-(trifluoromethyl)­phenyl]-4,5-dihydro-1*H*-pyrazole **(19)**

Yellow solid, yield: 46%, m.p: 103–105 °C. **FTIR ν_max_ (cm** ^**–1**^ **):** 1221 (C–F); 1462, 1464, 1525, 1608 (C=C); 2837, 2968 (C–H); 3293 (N–H). ^**1**^ **H NMR (600 MHz) (DMSO-** ***d***_**6**_ **/TMS) δ ppm:** 1.93–1.99 (dd, 4H, *J*_1,2_ = 16.2 Hz, protons of the pyrrolidine ring); 2.09 (s, 1H); 2.76–2.80 (dd, 1H, *J*_1,2_ = 10.8 Hz, pyrazoline ring CH_2_); 3.25 (t, 4H, *J*_1_ = 6.6, *J*_2_ = 7.2 Hz, protons of the pyrrolidine ring); 3.43–3.48 (dd, 1H, *J*_1,2_ = 10.2 Hz, pyrazoline ring CH_2_); 4.87 (t, 1H, *J*_1,2_ = 10.8 Hz, pyrazoline ring CH); 6.53 (d, 2H, *J* = 9.0 Hz); 7.45 (d, 2H, *J* = 9.0 Hz); 7.59 (t, 1H, *J*_1,2_ = 7.8 Hz); 7.63 (d, 1H, *J* = 9.0 Hz); 7.71 (d, 1H, *J* = 9.0 Hz); 7.74 (s, 1H). ^**13**^ **C NMR (151 MHz) (DMSO-** ***d***_**6**_ **/TMS) δ ppm:** 25.4, 47.7, 47.8, 56.4, 111.2, 111.8, 125.4, 126.7, 127.5, 130.47, 130.8, 133.7, 134.9, 148.4, 151.2, 151.8. Anal. Calcd for C_20_H_20_F_3_N_3_ (359.40 g/mol): C, 66.84; H, 5.61; N, 11.69%. Found: C, 66.89; H, 5.63; N, 11.75%. LC-MS (*m*/*z*): 360.10 [M + H]^+^.

#### 2.2.7 3-[4-(Pyrrolidin-1-yl)­phenyl]-5-[4-(trifluoromethyl)­phenyl]-4,5-dihydro-1*H*-pyrazole **(20)**

Yellow solid, yield: 45%, m.p: 95–97 °C. **FTIR ν_max_ (cm** ^**–1**^ **):** 1219 (C–F); 1485, 1510, 1526, 1610 (C=C); 2850, 2965 (C–H); 3345 (N–H). ^**1**^ **H NMR (600 MHz) (DMSO-** ***d***_**6**_ **/TMS) δ ppm:** 1.96 (t, *J*_1_ = 6.6, *J*_2_ = 14.4 Hz, 5H, (protons of the pyrrolidine ring, pyrazoline ring CH_2_), (NH, 1H); 2.71–2.75 (dd, 1H, *J*_1_ = 11.4, *J*_2_ = 10.8 Hz, pyrazoline ring CH_2_); 3.24 (t, 4H, *J*_1,2_ = 6.6, Hz, protons of the pyrrolidine ring); 4.75 (t, 1H, *J*_1_ = 10.2, *J*_2_ = 10.8 Hz, pyrazoline ring CH); 6.53 (d, 2H, *J* = 9.0 Hz); 7.16 (t, 2H, *J*_1_ = 8.4, *J*_2_ = 9.0 Hz); 7.41 (dd, 2H, *J*_1_ = 3.6, *J*_2_ = 5.4 Hz); 7.44 (d, 2H, *J* = 9.0 Hz). ^**13**^ **C NMR (151 MHz) (DMSO-** ***d***_**6**_ **/TMS) δ ppm:** 25.4, 46.6, 47.7, 47.8, 91.7, 111.7, 116.1, 121.0, 122.7, 126.6, 128.3, 130.2, 130.9, 151.5. Anal. Calcd for C_20_H_20_F_3_N_3_ (359.40 g/mol): C, 66.84; H, 5.61; N, 11.69%. Found: C, 66.89; H, 5.68; N, 11.77%. LC-MS (*m*/*z*): 360.10 [M + H]^+^.

#### 2.2.8 3-[4-(Pyrrolidin-1-yl)­phenyl]-5-[2-(trifluoromethoxy)­phenyl]-4,5-dihydro-1*H*-pyrazole **(21)**

Yellow solid, yield: 53%, m.p: 96–97 °C. **FTIR ν_max_ (cm** ^**–1**^ **):** 1226 (C–F); 1480, 1487, 1526, 1612 (C=C); 2851, 2960 (C–H); 3345 (N–H). ^**1**^ **H NMR (600 MHz) (DMSO-** ***d***_**6**_ **/TMS) δ ppm:** 1.79 (t, 4H, *J*_1,2_ = 6.6 Hz, protons of the pyrrolidine ring); 2.54–2.59 (dd, 1H, *J*_1,2_ = 10.2 Hz, pyrazoline ring CH_2_); 3.08 (t, 4H, *J*_1,2_ = 6.6 Hz, protons of the pyrrolidine ring); 3.16 (s, 1H); 3.24–3.28 (dd, 1H, *J*_1,2_ = 10.2 Hz, pyrazoline ring CH_2_); 4.82 (t, 1H, *J*_1_ = 10.8, *J*_2_ = 10.2 Hz, pyrazoline ring CH); 6.37 (d, 2H, *J* = 9.0 Hz); 7.20 (d, 1H, *J* = 7.8 Hz); 7.23–7.26 (m, 2H); 7.29 (d, 2H, *J* = 9.0 Hz); 7.49 (d, 1H, *J* = 7.2 Hz). ^**13**^ **C NMR (151 MHz) (DMSO-** ***d***_**6**_ **/TMS) δ ppm:** 25.4, 46.4, 47.7, 47.8, 111.2, 111.7, 120.5, 122.7, 128.5, 130.1, 130.6, 130.8, 131.5, 133.4, 151.2, 151.7. Anal. Calcd for C_20_H_20_F_3_N_3_O (375.40 g/mol): C, 63.99; H, 5.37; N, 11.19%. Found: C, 64.01; H, 5.41; N, 11.23%. LC-MS (*m*/*z*): 376.10 [M + H]^+^.

#### 2.2.9 3-[4-(Pyrrolidin-1-yl)­phenyl]-5-[3-(trifluoromethoxy)­phenyl]-4,5-dihydro-1H-pyrazole **(22)**

Brown solid, yield: 15%, m.p: 122–124 °C. **FTIR ν_max_ (cm** ^**–1**^ **):** 1212 (C–F); 1451, 1485, 1526, 1609 (C=C); 2844, 2966 (C–H); 3342 (N–H). ^**1**^ **H NMR (600 MHz) (DMSO-** ***d***_**6**_ **/TMS) δ ppm:** 1.95 (t, 4H, *J*_1,2_ = 6.6 Hz, protons of the pyrrolidine ring); 2.73–2.78 (dd, 1H, *J*_1_ = 10.8, *J*_2_ = 11. Hz, pyrazoline ring CH_2_); 3.24 (t, 4H, *J*_1_ = 6.6, *J*_2_ = 7.2 Hz, protons of the pyrrolidine ring); 3.31 (s, 1H); 3.41–3.45 (dd, 1H, *J*_1_ = 10.8, *J*_2_ = 10.2 Hz, pyrazoline ring CH_2_); 4.81 (t, 1H, *J*_1,2_ = 10.8 Hz, pyrazoline ring CH); 6.53 (d, 2H, *J* = 9.0 Hz); 7.26 (d, 1H, *J* = 8.4 Hz); 7.37 (s, 1H); 7.43–7.50 (m, 4H). ^**13**^ **C NMR (151 MHz) (DMSO-** ***d***_**6**_ **/TMS) δ ppm:** 25.4, 47.7, 47.8, 51.3, 111.2, 111.7, 111.8, 119.7, 124.8, 126.4, 126.7, 130.5, 130.8, 131.3, 149.4, 151.7. Anal. Calcd for C_20_H_20_F_3_N_3_O (375.40 g/mol): C, 63.99; H, 5.37; N, 11.19%. Found: C, 64.03; H, 5.39; N, 11.25%. LC-MS (*m*/*z*): 376.10 [M + H]^+^.

#### 2.2.10 3-[4-(Pyrrolidin-1-yl)­phenyl]-5-[4-(trifluoromethoxy)­phenyl]-4,5-dihydro-1*H*-pyrazole **(23)**

Yellow solid, yield: 47%, m.p: 127–129 °C. **FTIR ν_max_ (cm** ^**–1**^ **):** 1217 (C–F); 1461, 1487, 1527, 1610, (C=C); 2848, 2966 (C–H); 3322 (N–H). ^**1**^ **H NMR (600 MHz) (DMSO-** ***d***_**6**_ **/TMS) δ ppm:** 1.96 (t, 4H, *J*_1,2_ = 6.6 Hz, protons of the pyrrolidine ring); 2.74–2.79 (dd, 1H, *J*_1,2_ = 10.8 Hz, pyrazoline ring CH_2_); 3.24–3.26 (t, 4H, *J*_1,2_ = 6.6 Hz, protons of the pyrrolidine ring); 3.31 (s, 1H); 3.36–3.42 (dd, 1H, *J*_1_ = 10.4, *J*_2_ = 10.2 Hz, pyrazoline ring CH_2_); 4.79 (t, 1H, *J*_1_ = 10.8, *J*_2_ = 10.2 Hz, pyrazoline ring CH); 6.53 (d, 2H, *J* = 9.0 Hz); 7.33 (d, 2H, *J* = 9.0 Hz); 7.45 (d, 2H, *J* = 9.0 Hz); 7.51 (d, 2H, *J* = 8.4 Hz). ^**13**^ **C NMR (151 MHz) (DMSO-** ***d***_**6**_ **/TMS) δ ppm:** 25.0, 41.4, 47.7, 47.8, 111.7, 121.4, 121.6, 126.6, 127.2, 129.6, 130.4, 130.9, 134.6, 151.6. Anal. Calcd for C_20_H_20_F_3_N_3_O (375.40 g/mol): C, 63.99; H, 5.37; N, 11.19%. Found: C, 64.05; H, 5.41 N, 11.27%. LC-MS (*m*/*z*): 376.10 [M + H]^+^.

#### 2.2.11 5-[3,5-Bis­(trifluoromethyl)­phenyl]-3-[4-(pyrrolidin-1-yl)­phenyl]-4,5-dihydro-1*H*-pyrazole **(24)**

Yellow solid, yield: 41%, m.p: 156–158 °C. **FTIR ν_max_ (cm** ^**–1**^ **):** 1459, 1483, 1525, 1609 (C=C); 2852, 2916, 2964 (C–H); 3323 (N–H). ^**1**^ **H NMR (600 MHz) (DMSO-** ***d***_**6**_ **/TMS) δ ppm:** 1.96 (t, 4H, *J*_1,2_ = 6.6 protons of the pyrrolidine ring); 2.83–2.88 (dd, 1H, *J*_1_ = 11.4, *J*_2_ = 11. Hz, pyrazoline ring CH_2_); 3.25 (t, 4H, *J*_1_ = 6.6, *J*_2_ = 7.2 Hz, protons of the pyrrolidine ring); 3.49–3.54 (dd, 1H, *J*_1,2_ = 10.8 Hz, pyrazoline ring CH_2_); 4.99 (t, 1H, *J*_1,2_ = 10.8 Hz, pyrazoline ring CH); 6.53 (d, 2H, *J* = 9.0 Hz); 7.46 (d, 2H, *J* = 9.0 Hz); 8.02 (s, 1H); 8.10 (s, 2H). ^**13**^ **C NMR (151 MHz) (DMSO-** ***d***_**6**_ **/TMS) δ ppm:** 25.4, 41.5, 47.7, 47.9, 111.9, 124.7, 126.7, 127.8, 128.3, 130.6, 130.9, 131.3, 138.2, 151.9. Anal. Calcd for C_21_H_19_F_6_N_3_ (427.39 g/mol): C, 59.02; H, 4.48; N, 9.83%. Found: C, 59.05; H, 4.51; N, 9.87%. LC-MS (*m*/*z*): 428.10 [M + H]^+^.

#### 2.2.12 5-[2-Fluoro-3-(trifluoromethyl)­phenyl]-3-[4-(pyrrolidin-1-yl)­phenyl]-4,5-dihydro-1*H*-pyrazole **(25)**

Yellow solid, yield: 52%, m.p: 111–113 °C. **FTIR ν_max_ (cm** ^**–1**^ **):** 1210 (C–F); 1485, 1525, 1594, 1608 (C=C); 2844, 2967 (C–H); 3343 (N–H). ^**1**^ **H NMR (600 MHz) (DMSO-** ***d***_**6**_ **/TMS) δ ppm:** 1.79 (t, 4H, *J*_1,2_ = 6.6 Hz, protons of the pyrrolidine ring); 2.63–2.68 (dd, 1H, *J*_1,2_ = 10.2 Hz, pyrazoline ring CH_2_); 3.08 (t, 4H, *J*_1,2_ = 6.6 Hz, protons of the pyrrolidine ring); 3.31–3.35 (dd, 1H, *J*_1_ = 10.8, *J*_2_ = 10.8 Hz, pyrazoline ring CH_2_); 3.70 (s, 1H); 4.86 (t, 1H, *J*_1_ = 10.8, *J*_2_ = 10.2 Hz, pyrazoline ring CH); 6.37 (d, 2H, *J* = 9.0 Hz); 7.24 (d, 1H, *J* = 7.8 Hz); 7.31 (d, 2H, *J* = 8.4 Hz); 7.54 (t, 1H, *J*_1,2_ = 7.2 Hz); 7.69 (t, 1H, *J*_1_ = 7.8, *J*_2_ = 6.6 Hz). ^**13**^ **C NMR (151 MHz) (DMSO-** ***d***_**6**_ **/TMS) δ ppm:** 25.4, 45.4, 47.7, 47.8, 111.7, 125.5, 126.4, 127.3, 128.5, 129.2, 130.2, 132.6, 151.5. Anal. Calcd for C_20_H_19_F_4_N_3_ (377.39 g/mol): C, 59.02; H, 4.48; N, 9.83%. Found: C, 59.05; H, 4.51; N, 9.87%. LC-MS (*m*/*z*): 296.10 [M + H]^+^.

#### 2.2.13 5-[2-Fluoro-4-(trifluoromethyl)­phenyl]-3-[4-(pyrrolidin-1-yl)­phenyl]-4,5-dihydro-1*H*-pyrazole **(26)**

Yellow solid, yield: 51%, m.p: 109–110 °C. **FTIR ν_max_ (cm** ^**–1**^ **):** 1216 (C–F); 3288 (N–H); 2966, 2847 (C–H); 1485, 1526, 1595, 1609 (C=C); ^**1**^ **H NMR (600 MHz) (DMSO-** ***d***_**6**_ **/TMS) δ ppm:** 1.95 (t, 4H, *J*_1_ = 3.6, *J*_2_ = 6.6 Hz, protons of the pyrrolidine ring); 2.78–2.83 (dd, 1H, *J*_1_ = 10.2 Hz, pyrazoline ring CH_2_); 3.24 (t, 4H, *J*_1,2_ = 6.6 Hz, protons of the pyrrolidine ring); 3.32 (s, 1H); 3.46–3.50 (dd, 1H, *J*_1,2_ = 10.8 Hz, pyrazoline ring CH_2_); 5.00 (t, 1H, *J*_1_ = 10.2, *J*_2_ = 10.8 Hz, pyrazoline ring CH); 6.53 (d, 2H, *J* = 9.0 Hz); 7.45 (d, 2H, *J* = 9.0 Hz); 7.60 (d, 1H, *J* = 8.4 Hz); 7.68 (d, 1H, *J* = 10.2 Hz); 7.73 (t, 1H, *J*_1,2_ = 7.8 Hz). ^**13**^ **C NMR (151 MHz) (DMSO-** ***d***_**6**_ **/TMS) δ ppm:** 25.4, 40.2, 47.7, 47.9, 111.2, 111.8, 111.9, 120.5, 122.2, 126.8, 127.6, 130.4, 130.9, 131.5, 148.5, 151.3. Anal. Calcd for C_20_H_19_F_4_N_3_ (377.39 g/mol): C, 63.65; H, 5.07; N, 11.13%. Found: C, 63.68; H, 5.10; N, 11.15%. LC-MS (*m*/*z*): 378.10 [M + H]^+^

#### 2.2.14 5-[4-Fluoro-3-(trifluoromethyl)­phenyl]-3-[4-(pyrrolidin-1-yl)­phenyl]-4,5-dihydro-1*H*-pyrazole **(27)**

Brown solid, yield: 53%, m.p: 97–98 °C. **FTIR ν_max_ (cm** ^**–1**^ **):** 1259 (C–F); 1506, 1526, 1593, 1608 (C=C); 2845, 2967 (C–H); 3299 (N–H). ^**1**^ **H NMR (600 MHz) (DMSO-** ***d***_**6**_ **/TMS) δ ppm:** 1.95 (t, 4H, *J*_1_ = 3.0, *J*_2_ = 3.6 Hz, protons of the pyrrolidine ring); 2.71–2.76 (dd, 1H, *J*_1,2_ = 10.8, Hz, pyrazoline ring CH_2_); 3.25 (t, 4H, *J*_1_ = 6.6, *J*_2_ = 9.6 Hz, protons of the pyrrolidine ring); 3.31 (s, 1H); 3.36–3.40 (dd, 1H, *J*_1,2_ = 10.2 Hz, pyrazoline ring CH_2_); 4.78 (t, 1H, *J*_1,2_ = 10.8 Hz, pyrazoline ring CH); 6.54 (t, 2H, *J*_1_ = 9.0, *J*_2_ = 7.2 Hz); 7.24 (d, 1H, *J* = 8.4 Hz); 7.44 (d, 1H, *J* = 9.0 Hz); 7.62 (d, 2H, *J* = 9.0 Hz); 7.81 (t, 1H, *J*_1_ = 9.0, *J*_2_ = 6.6 Hz). ^**13**^ **C NMR (151 MHz) (DMSO-** ***d***_**6**_ **/TMS) δ ppm:** 25.4, 47.7, 47.7, 56.5, 111.2, 112.9, 113.4, 122., 124.7, 126.6, 127.3, 130.9, 132.2, 138.1, 148.2, 151.2. Anal. Calcd for C_20_H_19_F_4_N_3_ (377.39 g/mol): C, 63.65; H, 5.07; N, 11.13%. Found: C, 63.70 H, 5.12; N, 11.17%. LC-MS (*m*/*z*): 378.10 [M + H]^+^.

### 2.3 Antidiabetic Activity Assays

The antidiabetic inhibitory activity of novel pyrrolidine-based pyrazolines **(14–27)** derivatives was tested against α-amylase and α-glucosidase. α-Amylase and α-glucosidase inhibition procedures were tested according to the procedure previously reported by our research group.^37^ For details of α-amylase^42^ and α-glucosidase^43^ inhibition activity procedures, see the Supporting Information.

### 2.4 *In Silico* Studies

#### 2.4.1 Molecular Docking Simulations

Molecular docking is a computational technique used to predict the potential interactions between target proteins and small molecules. It plays a pivotal role in modern drug discovery strategies and is widely utilized in applications such as virtual screening, lead compound identification, and structure-based drug design.^44,45^ In this study, the three-dimensional structure of the α-glucosidase enzyme was obtained from the Protein Data Bank (PDB ID: 5NN4) (http://www.rcsb.org/pdb). It underwent standard preprocessing procedures to ensure the structure’s suitability for docking calculations. Crystallographic water molecules and ions were removed, and hydrogen atoms were added to the structure under neutral pH conditions (pH 7.0).

The binding site was determined using the AGFR 1.2 program^46^ to include the catalytic GH31 domain of the α-glucosidase enzyme. The center of the binding site was defined using Cartesian coordinates (*x* = −12.941, *y* = −29.032, and *z* = 97.326). The dimensions of the grid box were set to 62 × 68 × 66 points with a grid spacing of 0.375 Å. This setup was used to generate the grid parameter file (.gpf). By the way, 3D structures of pyrrolidine-based pyrazoline derivatives (compounds **14–27**) were constructed, hydrogen atoms were added, and geometry optimizations were performed using Discovery Studio Client.^47^ Molecular docking simulations were performed using AutoDock 4.2 ^48^ according to standard protocols, where the receptor was kept rigid and the ligands were treated as flexible. The Lamarckian Genetic Algorithm (LGA) was employed with 100 independent runs for each ligand, allowing a maximum of 2,500,000 energy evaluations and 27,000 generations per run.

AutoDock utilizes an empirical free energy scoring function to estimate ligand–receptor binding affinity. This function incorporates van der Waals, electrostatic, and hydrogen bonding interactions as well as desolvation effects and torsional entropy penalties. The total binding free energy (Δ*G*_binding) is calculated as the sum of these components, allowing for an approximate prediction of binding strength and inhibition constant (*K*_i_) for each ligand.

The overall binding free energy (Δ*G*_binding) is computed as the sum of the individual energy components:

$$\Delta G_{\mathrm{binding}}=\Delta G_{\mathrm{vdW}}+\Delta G_{H-\mathrm{bond}}+\Delta G_{\mathrm{electrostatics}}+\Delta G_{\mathrm{desolvation}}+\Delta G_{\mathrm{torsion}}$$

This empirical scoring function enables AutoDock to rank docking poses and estimate the inhibition constant (*K*_i_), providing an approximation of the ligand’s binding affinity based on thermodynamic principles. Based on this scoring, AutoDock ranks docking poses and estimates the inhibition constant (*K*_i_) for each compound.

#### 2.4.2 Molecular Dynamics Simulation

To further evaluate the temporal stability of the docked complexes, molecular dynamics (MD) simulations were performed for the α-glucosidase enzyme in complex with compounds **18**, **21**, and **22**, which demonstrated the highest binding affinities, comparable to that of the reference compound, acarbose. The simulations were performed for 100 ns (ns) utilizing Desmond software, with configuration steps executed via the Maestro 13.8 graphical interface.^49^ Each protein–ligand complex was embedded in an orthorhombic simulation box solvated with TIP3P water molecules,^50^ and Na^+^ ions were added at a concentration of 0.15 M to neutralize the system. The OPLS4 force field was employed for molecular force calculations. Simulations were run under the NPT ensemble, maintaining a constant particle number, pressure, and temperature. The temperature was kept at 300 K using the Nose–Hoover thermostat, while the pressure was maintained at 1.01325 bar via the Martyna–Tobias–Klein barostat. Long-range electrostatic interactions were computed using the Particle Mesh Ewald (PME) method, while a 9.0 Å cutoff was applied for short-range electrostatic and van der Waals interactions.^51^ Simulation trajectories were analyzed to evaluate the structural behavior of the complexes over time. Metrics such as root-mean-square deviation (RMSD) and root-mean-square fluctuation (RMSF) were calculated alongside analyses of hydrogen bonding, hydrophobic interactions, ionic contacts, and water-mediated bridges. These results provide insights into the stability and flexibility of the ligand–enzyme complexes and offer valuable guidance for the rational design of novel structure-based α-glucosidase inhibitors.

## 3 Results and Discussion

### 3.1 Chemistry

5-[substituted phenyl]-3-[4-(pyrrolidin-1-yl)­phenyl]-4,5-dihydro-1*H*-pyrazoles **(14–27)** were synthesized by treating (*E*)-3-[substituted phenyl]-1-[4-(pyrrolidin-1-yl)­phenyl]­prop-2-en-1-ones **(1–13)**, which we synthesized previously, with hydrazine monohydrate. In the synthesis of target compounds, the microwave-assisted method was used, which is a faster and more environmentally friendly method, due to some disadvantages of the traditional method, such as higher temperatures, longer reaction times, byproduct formation, and harmful environmental effects.^52,53^ The synthesis pathway and R groups of 5-[substituted phenyl]-3-[4-(pyrrolidin-1-yl)­phenyl]-4,5-dihydro-1*H*-pyrazoles **(14** **–** **27)** are shown in Scheme 1. Pyrazolines containing pyrrolidine rings were synthesized in 53–15% yield.

**Scheme 1:**
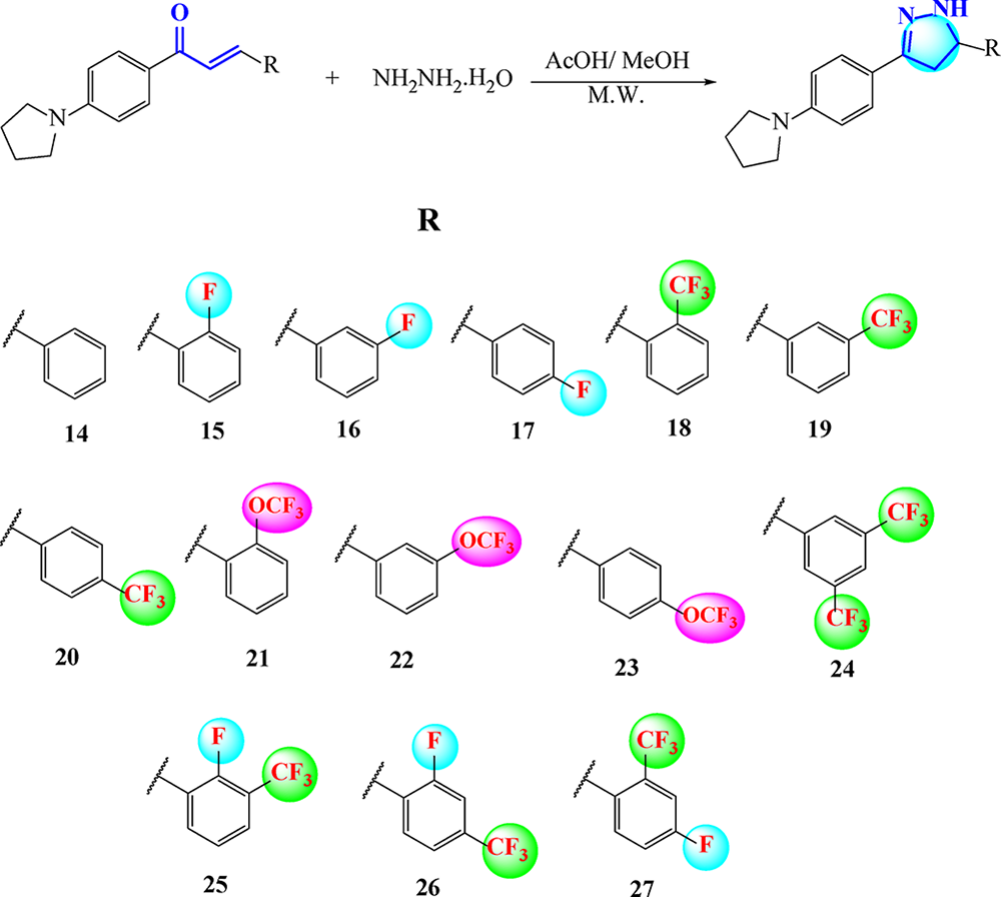
Synthetic Pathway of 5-[Substituted phenyl]-3-[4-(pyrrolidin-1-yl)­phenyl]-4,5-dihydro-1*H*-pyrazoles **(14**–**27)**

One of the most important pieces of evidence for the O=C–C=C structure in the chalcone structure into a pyrazoline ring is the observation of the N–H peak, while no C=O stretching bands were observed. The N–H stretching bands of compounds **14**–**27** were observed between 3361 and 3196 cm^–1^. Aromatic C–H stretching bands were detected in the range of 3049–2960 cm^–1^; C=C stretching bands were detected in the range of 1612–1451 cm^–1^; and C–F stretching bands were detected in the range of 1259–1210 cm^–1^.

When the ^1^H NMR and ^13^C NMR spectra of the pyrazoline derivatives were examined, the CH_2_ peaks belonging to the pyrrolidine ring were detected as triplets at 1.79–1.96 and 3.08–3.25 ppm, respectively. The most important evidence for the formation of the pyrazoline ring was the observation of CH_2_ peaks as double doublets at 2.54–2.88 and 3.19–3.54 ppm. In addition, the CH peak of the pyrazoline ring was detected as a triplet at 4.58–5.04 ppm, and the proton of NH was detected in the range of 1.96–3.70 ppm.

### 3.2 Antidiabetic Activity and SAR Study

The antidiabetic activity of the pyrrolidine–pyrazoline hybrid molecules **(14–27)** was determined against α-glucosidase and α-amylase compared to that of acarbose as a reference standard. The α-amylase and α-glucosidase inhibition activity results of new molecules are given in Table 1. Although the nonsubstituted compound **14** (IC_50_: 110.71 ± 1.51 μM) exhibited the best activity against α-amylase, it was not more effective than acarbose (IC_50_: 85.56 ± 1.56 μM). Therefore, SAR was not discussed due to the low α-amylase inhibitory activity of the compounds.

**Table 1:**
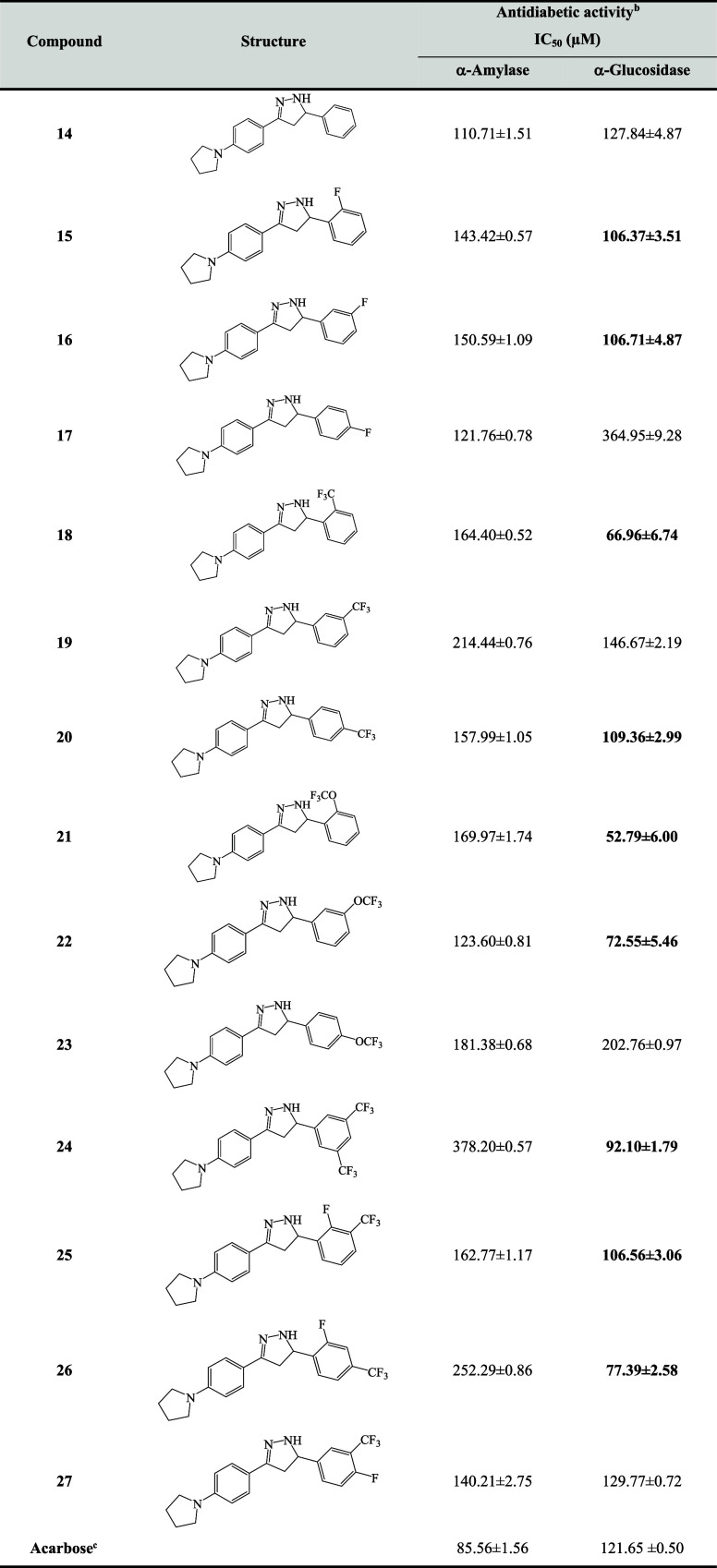
Antidiabetic Activities of Pyrrolidine-Based Pyrazoline Derivatives (**14–27**)­^a^

a Values expressed herein are mean ± SEM of three parallel measurements. *p* < 0.05.

b All compounds for α-amylase α-glucosidase assay are in the concentration range of 25–50–100–200 μM.

c Reference compounds.

According to the α-glucosidase activity results, the most potent inhibitors were determined to be compounds **21** (IC_50_: 52.79 ± 6.00 μM), **18** (IC_50_: 66.96 ± 6.74 μM), **22** (IC_50_: 72.55 ± 5.46 μM), **26** (IC_50_: 77.39 ± 2.58 μM), and **24** (IC_50_: 92.10 ± 1.79 μM) with the lowest IC_5__0_ values. Compound **21** was found to be approximately 2 times more effective than the IC_5__0_ of acarbose (121.65 ± 0.50 μM).

When the SAR of new compounds against α-glucosidase was evaluated, a significant increase in activity was observed compared to compound **14** (IC_5__0_: 127.84 μM), carrying a nonsubstituted phenyl ring by attaching fluorinated substituents to different positions of the phenyl ring, except for compounds **17, 19**, **23** and **27.**

As demonstrated in Table 1, the presence of a fluorine atom at the 2- and 3-positions of the phenyl ring (compounds **15** and **16**; IC_5__0_ ∼ 106 μM), resulted in an enhancement in the activity observed, while the fluorine atom located at 4-position (compound **17**; IC_5__0_: 364.95 μM) exhibited a notable decrease in activity. Similar results were determined for pyrazolines bearing a trifluoromethoxy (OCF_3_) group at the phenyl ring. Compound **21** having 2-OCF_3_ and compound **22** having 3-OCF_3_ group exhibited the most potent activity with IC_5__0_ values of 52.79 and 72.55 μM, respectively. However, the activity of compound **23** (IC_5__0_: 202.76 μM) decreased significantly when the OCF_3_ group was in position 4 of the phenyl ring. It seems that when the inhibitory activities of compounds with trifluoromethyl (CF_3_) groups at the 2-, 3-, and 4- positions of the phenyl ring were examined, it was determined that compound **18** (IC_5__0_: 66.96 μM) was more active than compounds **19** and **20** carrying the same substituent. It was also observed that the activity decreased in other positions of the phenyl ring. When compound **18** (CF_3_) and compound **21** (OCF_3_) were compared, it was determined that the presence of the oxygen atom had a positive effect on the activity.

It has been observed that good activity was detected in compounds **24**, **25**, and **26** where the F atom and CF_3_ groups were disubstituted at different positions. It was determined that the activity of compound **24** (IC_5__0_: 92.10 μM) was better with a double CF_3_ group in the 3- and 5-positions of the phenyl ring. Compound **25** carrying 2-F and 3-CF_3_ exhibited moderate activity (IC_5__0_: 106.56 μM), and the compound **26** having 2-F and 4-CF_3_ exhibited better activity (IC_5__0_: 77.39 μM). The activity was reduced in compound **27,** which contained 3-CF_3_ and 4-F (IC_5__0_: 129.77 μM).

Compounds bearing −OCF_3_ groups have exhibited better inhibitor performance due to both electron attraction and hydrophilic–hydrophobic balance. Compounds bearing pyrazoline and pyrrolidine rings bind to the active site of α-glucosidase and prevent substrate binding. As a result, the substitution of −OCF_3_ at position 2 of the phenyl ring exhibited the best activity and showed the strongest inhibitory effect. Compounds still showed good activity in the presence of −CF_3_, although not as much as that of −OCF_3_. It was noticeable that the F atom showed moderate activity at the 2- and 3-positions, while its substitution at the 4-position decreased the activity.

While the α-glucosidase inhibitor activity decreased with the R_3_ substitution of −OCF_3_, it was observed that the substitution of −CF_3_ at the 4-position kept the activity at a moderate level. The highest inhibitory effect was provided by the substitution of the −OCF_3_ group at the 2-position (compound **21**). While the CF_3_ group at the 2-position of the phenyl ring exhibited higher activity (compound **18**), it was observed that the activity was reduced when this group was located at 3 and 4- positions (compounds **19** and **20**). It was demonstrated that polysubstituted CF_3_ groups (compound **24**) increased the inhibitory effect. The F atom at 4-position (compound **17**) significantly reduced the activity, but the fluorine at the 2- and 3- positions (compounds **15** and **16**) had a positive effect. Especially the fluorine and −OCF_3_ groups at the 4-position of the phenyl ring generally decreased the activity. SAR showed that organofluorine modifications at different positions on the phenyl ring markedly changed the inhibitory effect.

The effect of pyrrolidine-based pyrazolines, where −F, −CF_3,_ and −OCF_3_ groups were positioned on the phenyl ring, on α-glucosidase inhibition was generally in the direction of increasing the biological activity of the molecule by these electron-withdrawing and hydrophobic substituents. The −F, −CF_3,_ and −OCF_3_ groups had strong electron-withdrawing properties. This property reduced the electron density of the phenyl ring and caused the pyrazoline core to change its binding affinity to the enzyme. It was hypothesized that this situation had a positive effect on the activity by binding the molecule more strongly or in a suitable conformation in the active site of the enzyme. Since the −CF_3_ and −OCF_3_ groups provided high hydrophobicity to the molecules, it was seen that they increased the inhibitory activity by strengthening the interaction with the hydrophobic pockets in the active site of the α-glucosidase, especially in their substitutions in R_1_. The substituents on the phenyl ring had a significant effect on the affinity and selectivity of the inhibitor together with the pyrrolidine–pyrazole core.

### 3.3 Molecular Docking Analysis

Since none of the compounds, except compound **14**, exhibited significant inhibitory activity against α-amylase, docking studies were performed only for the α-glucosidase enzyme. The molecular docking studies have elucidated the binding interactions and binding affinities between the α-glucosidase enzyme and synthetic compounds. As the reference compound, the binding energy of acarbose was determined to be −**4.66** kcal/mol. These compounds exhibit lower (i.e., more negative) binding energies than the reference ligand (Table 2)**.** Among these, compounds **18** (−6.67 kcal/mol), **21** (−6.54 kcal/mol), and **22** (−7.23 kcal/mol) demonstrated particularly notable binding scores, indicative of substantial inhibitory potential. These compounds also showed favorable IC_5__0_ values of 66.96 ± 6.74, 52.79 ± 6.00, and 72.55 ± 5.46 μM, respectively, reinforcing their significance as promising enzyme inhibitors. In the binding position of compound **18**, π–π T-stacking interactions with *Trp376*, π-alkyl interactions with *Leu650* and *Leu678*, and aliphatic interactions with Trp481 and Met519 were noted. Furthermore, hydrogen bonds with *Asp616* and *Arg600* and van der Waals interactions with adjacent residues have been observed. The binding structure of compound **21** reveals alkyl contacts with *Trp481* and *Met519*, π-alkyl interactions with *Leu650* and *Leu678*, and a hydrogen bond with the *Asp616* residue. Furthermore, many van der Waals interactions were detected with *Trp618, Gly651, Ser676, Ser679, Leu677, Phe649*, and *Trp376*. Compound **22** demonstrated a π–π stacking contact with Trp376, π-alkyl interactions with *Leu650* and *Leu678*, and alkyl interactions with *Trp481* and *Met519*. Furthermore, it established standard hydrogen bonds with *Asn652, Ser679,* and *Asp616*, carbon–hydrogen bonds with *Gly651* and *Leu678*, and halogen bonds involving fluorine atoms. The molecule formed van der Waals interactions with adjacent residues (Figure 3). The *in silico* data indicate that compounds **18**, **21**, and **22** can bind with high specificity to the active site of the α-glucosidase enzyme and demonstrate inhibitory effects through diverse interactions with essential residues. The overlap of the amino acids with which these compounds interact, identified in prior literature as the enzyme’s binding pockets, corroborates the precision of the *in silico* studies.^54^

**Table 2:** Molecular Docking Analysis of Compounds with *the* α-Glucosidase Enzyme: Binding Energy and Amino Acid Interactions

compound number	α-glucosidase binding energy (kcal/mol)	interactions with amino acids^a^
**acarbose** ^b^	–4.66	**Leu677, Leu678, Asp404** *,* **Asp282**
**14**	–7.03	**Asp616** *, Met519, Trp481, Leu650, Leu678*
**15**	–6.81	**Asp616,** *Trp376, Met519, Trp481, Leu650, Leu678*
**16**	–6.99	**Gly651, Asp616** *, Trp376, Met519, Trp481, Leu650, Leu678*
**17**	–6.37	**Asp616,** *Met519, Trp481, Leu650, Leu678*
**18**	–6.67	**Asp616,** *Trp376, Met519, Trp481, Leu650, Leu678*
**19**	–7.06	**Ser679, Gly651, Asp616,** *Trp376, Met519, Leu678, Trp481, Leu650, Leu678*
**20**	–6.16	**Asp404, Asp616, His674, Ser676,** *Leu678, Trp516, Trp613, Phe649, His674, Leu650*
**21**	–6.54	**Asp616,** *Met519, Leu650, Trp481, Leu678*
**22**	–7.23	**Asn652, Gly651, Ser679, Asp616,** *Leu678, Trp376, Met519, Trp481, Leu650*
**23**	–6.16	**Trp613, Asp616, Arg672, His674, Ser676,** *Asp645, Asp518, Phe649, Leu650, Leu678, Trp516, Trp613,*
**24**	–6.56	**Arg281** *,* **Ala284** *,* **Asn524** *,* **Leu283** *,* **Ala555** *, Asp282, Ser523, Trp481, Trp516, Phe525, Trp613, Phe649, His674*
**25**	–6.43	**Arg281** *,* **Leu283** *, Asp518, Asp282, Trp481, Trp516, Phe525, Phe649, His674, Ala555*
**26**	–6.27	**Asp404** *,* **Asp616** *, Asp518, Ser676, His674, Leu678, Phe649, Leu650*
**27**	–6.26	**Arg281** *,* **Leu283** *, Asp282, Trp481, Trp516, Phe525, Phe649, His674, Ala555, Met519*

a The amino acids highlighted in bold signify hydrogen bond formation.

b Reference compound.

**Figure 3:**
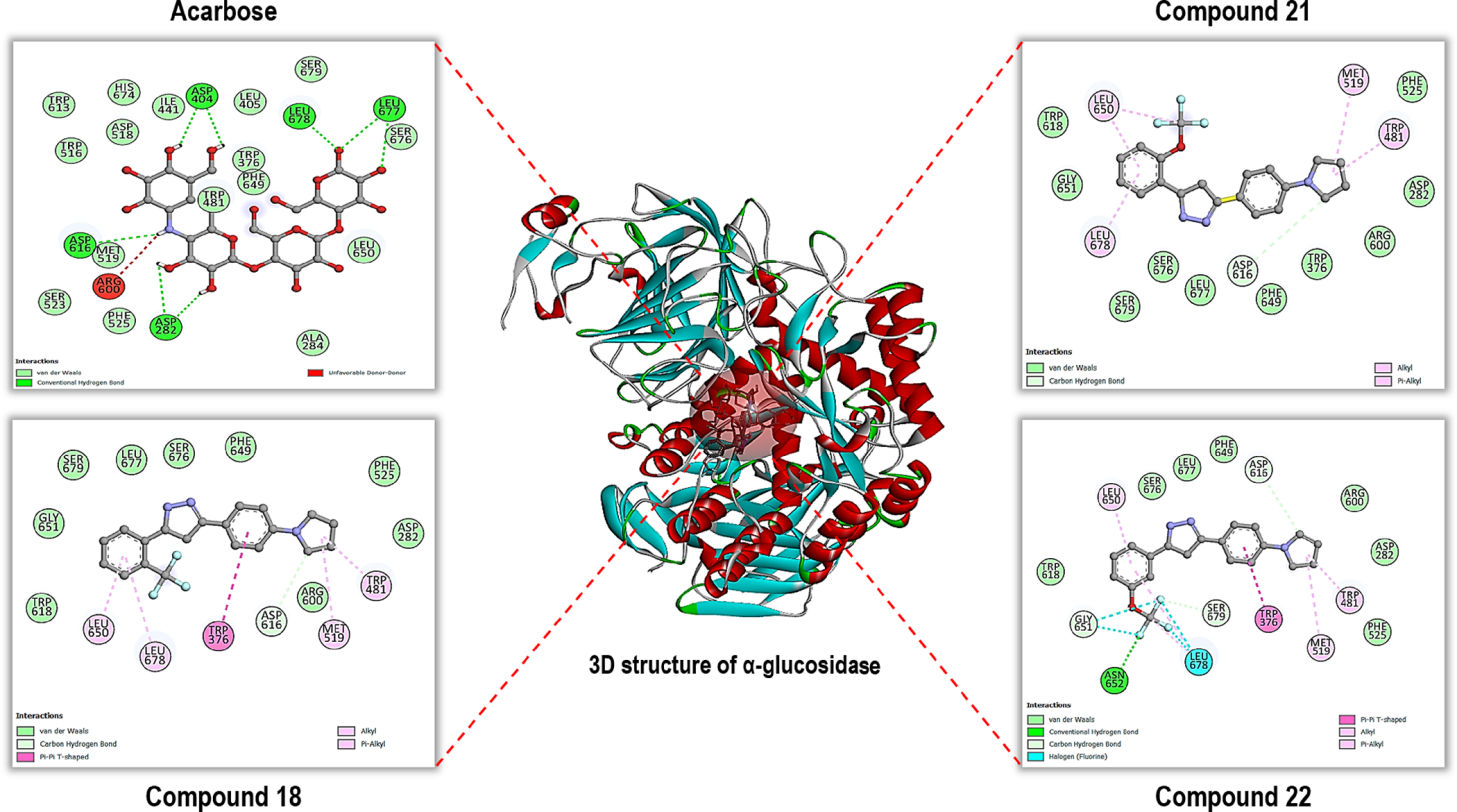
2D analysis of the lowest-energy binding conformations of compounds **18**, **21**, and **22**, which exhibit the best binding affinities and biological activity for α-glucosidase.

### 3.4 Analysis of MD Simulation

This study assessed the dynamic stability and binding affinity of complexes generated by active compounds (**18**, **21**, and **22**) interacting with the α-glucosidase enzyme and the reference molecule, acarbose, throughout a 100 ns simulation period utilizing Desmond software. The simulations yielded comprehensive insights into the complexes’ structural characteristics and binding mechanisms. Analyses of RMSD performed to assess the overall stability of the complexes have indicated conformational alterations at both the protein and ligand levels. The RMSD values for the Cα atoms of the α-glucosidase protein varied from 0.4 to 1.6 Å in the acarbose complex; for compound **18**, they ranged from 0.6 to 1.8 Å, for compound **21**,they ranged from 0.5 to 2.0 Å, and for compound **22**, they ranged from 0.4 to 1.8 Å. The RMSD values in the binding regions of the ligands were as follows: acarbose exhibited values between 0.6 and 4.2 Å; compound **18** ranged from 0.2 to 1.8 Å; compound **21** ranged from 1.5 to 2.3 Å; and compound **22** ranged from 0.2 to 2.2 Å (Figures 4 and 5)**.** These results suggest that the more active compounds maintained greater stability in the binding pocket compared to acarbose.

**Figure 4:**
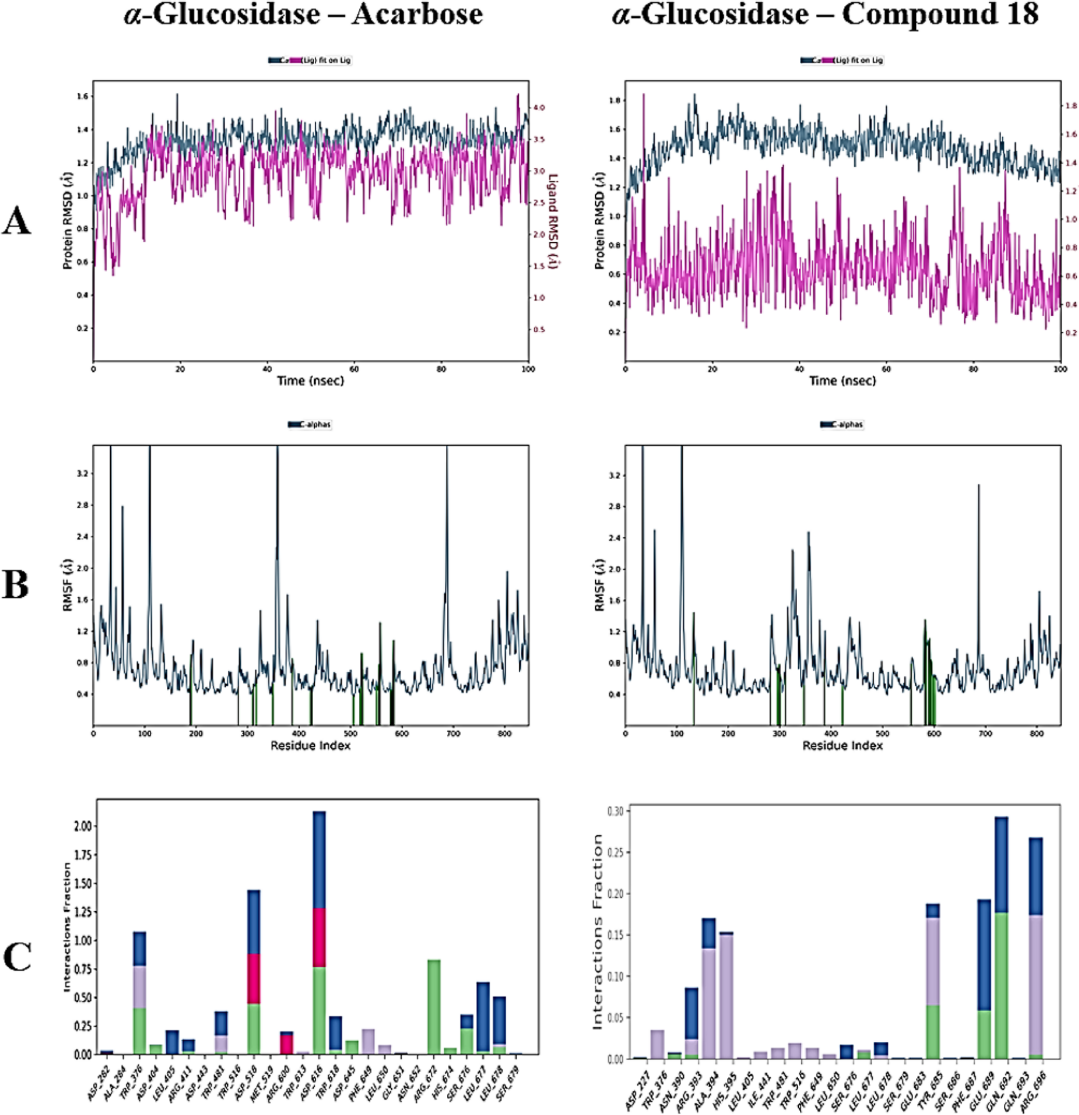
MD simulation results for the α-glucosidase complexes with the reference compound acarbose and compound **18**. (A) RMSD profiles of the protein (blue line) and ligand (pink line) over the 100 ns simulation, illustrating structural stability of the complexes. (B) RMSF analysis of α-glucosidase residues, indicating the flexibility profile upon ligand binding. (C) Stacked bar charts depicting the persistence of protein–ligand interactions throughout the simulation trajectory, expressed as a percentage of total simulation time. Interaction types are color-coded as follows: hydrogen bonds (green), hydrophobic contacts (purple), ionic interactions (pink), and water bridges (blue).

**Figure 5:**
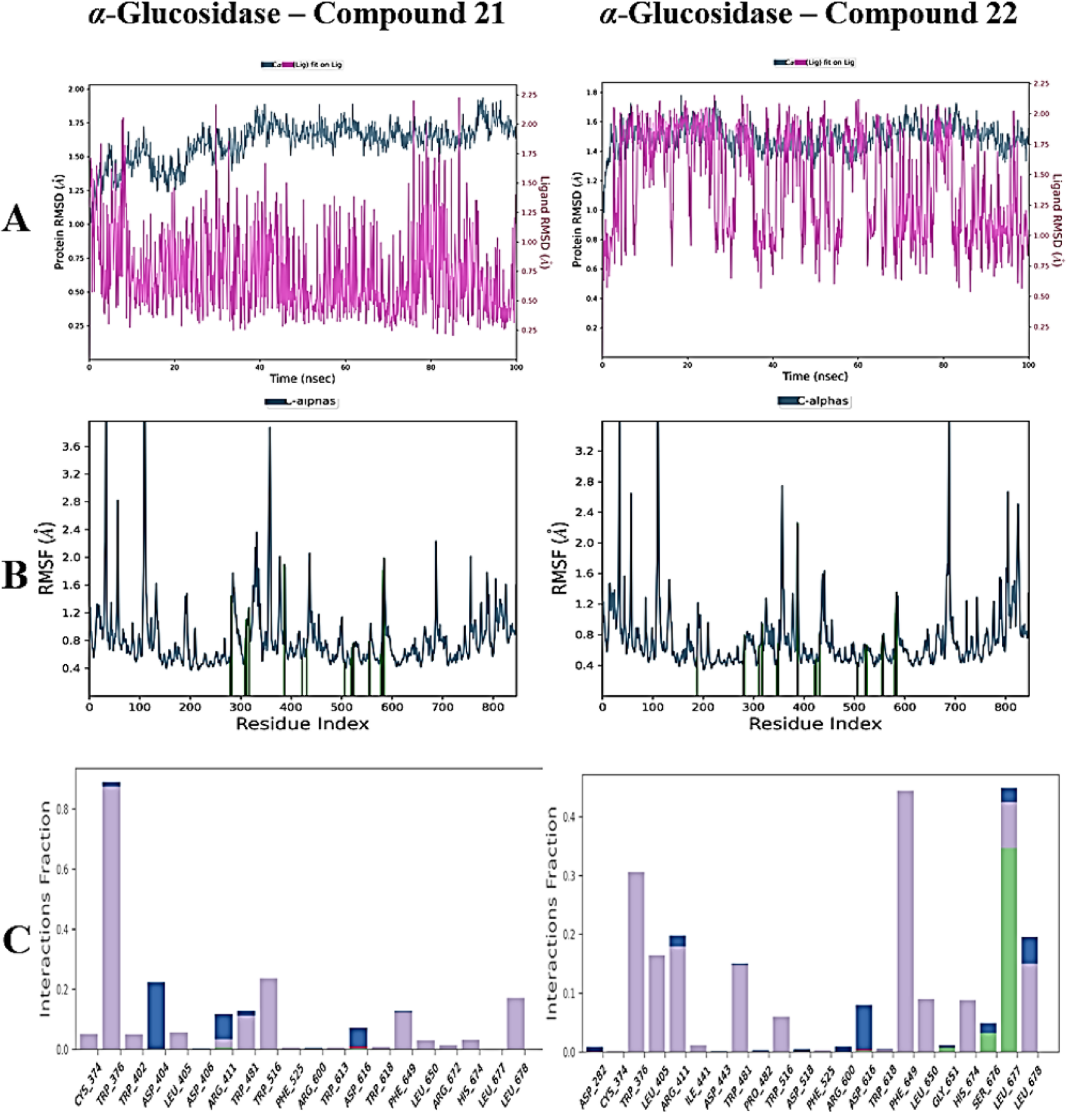
MD simulation results for the α-glucosidase complexes with the compound **21** and **22**. (A) RMSD profiles of the protein (blue line) and ligand (pink line) over the 100 ns simulation, illustrating the structural stability of the complexes. (B) RMSF analysis of α-glucosidase residues, indicating the flexibility profile upon ligand binding. (C) Stacked bar charts depicting the persistence of protein–ligand interactions throughout the simulation trajectory, expressed as a percentage of total simulation time. Interaction types are color-coded as follows: hydrogen bonds (green), hydrophobic contacts (purple), ionic interactions (pink), and water bridges (blue).

In the RMSF analysis, which assesses the flexibility of protein residues, minimal fluctuation values were noted in all complexes, with the exception of the terminal regions. The RMSF values were recorded as follows: 0.4–3.2 Å for amino acids in α-glucosidase–acarbose, 0.4–3.2 Å for amino acids in α-glucosidase–compound **18**, 0.4–3.6 Å for amino acids in α-glucosidase–compound **21**, and 0.4–3.2 Å for amino acids in α-glucosidase–compound **22** (Figures 4 and 5)**.** This circumstance suggests that the residues next to the ligand-binding areas demonstrate significant stability.

The persistence of noncovalent interactions within the protein–ligand complexes during MD simulations was thoroughly examined. The reference compound, acarbose, consistently maintained hydrogen bonds with *Arg672* and *Asp645*, alongside hydrophobic and water-bridged interactions involving *Trp376, Arg672, Leu677, Leu678, Ser676,* and *Trp618*. Compound **18** exhibited stable hydrogen bonds and water bridges with *Gln692* and *Glu689*, while also forming hydrophobic interactions with *Trp376, His395, Ile441, Trp516, Phe649*, and *Leu650*. Compound **21** preserved water bridges with *Asp404, Arg411*, and Asp616 and displayed hydrophobic interactions with *Cys374, Trp376, Trp402, Leu404, Trp516, Phe649*, and *Leu678*. Compound **22** demonstrated hydrogen bond interactions and water bridges with *Ser676* and *Leu677*, and hydrophobic contacts with *Trp376, Leu405, Arg411, Ile441, Trp481*, *Trp516, Phe649, Leu650* ,and *His674* (Figures 4 and 5)**.** These sustained interactions contribute to the binding stability observed during simulation and further support the inhibitory potential of these compounds.

The binding free energies derived from the simulation data were computed via the MM-GBSA method. This investigation determined the binding energies as follows: −32.33 kcal/mol for acarbose, −26.24 kcal/mol for compound **18**, −25.84 kcal/mol for compound **21**, and −22.82 kcal/mol for compound **22**. The results demonstrate that although the selected candidate compounds have comparatively lower binding energies than the reference molecule, they can form stable and substantial interactions with the enzyme.

### 3.5 *In Silico* ADME Studies

*In silico* analysis of absorption, distribution, and physicochemical properties was conducted primarily to evaluate the synthesis molecules’ drug likeness and pharmacokinetic behavior. The pharmacokinetic appropriateness of compounds **14–27** was assessed utilizing the SwissADME platform.^55^ The molecular weights of the compounds varied from 291.39 to 427.39 g/mol, with all remaining beneath the 500 g/mol limit. The quantity of hydrogen bond acceptors (HBA) ranged from 1 to 7, while the number of hydrogen bond donors (HBD) remained constant at 1 for all compounds. The number of rotatable bonds (NRB) ranged from 3 to 5. The topological polar surface area (TPSA) computed values ranged from 27.63 to 36.86 Å^2^. The computed *i*Log*P* values varied from 2.96 to 3.56. All compounds, except compound **24**, were classed as exhibiting high gastrointestinal absorption; compound **24** was designated as having low absorption. Regarding blood–brain barrier permeability, only compound **24** was forecasted to be nonpermeant, while all other compounds were deemed permeant. Following Lipinski’s rule of five, all compounds exhibited zero or one infraction adherence. The synthetic accessibility ratings ranged from 3.28 to 3.66 (Table 3, Figures 6 and 7)**.**

**Table 3:** *In Silico* ADME and Drug-likeness Assessment of Compounds **14–27** ^a^

**compound number**	*M*_W_ (g/mol)	HBA (≤10)	HBD (≤5)	NRB (≤140 A^2^)	TPSA Å^2^	*I*Log *P*	GI absorption	BBB permeant	Lipinski	synthetic accessibility
**14**	291.39	1	1	3	27.63	2.96	high	yes	0	3.28
**15**	309.38	2	1	3	27.63	3.10	high	yes	0	3.43
**16**	309.38	2	1	3	27.63	3.22	high	yes	0	3.36
**17**	309.38	2	1	3	27.63	3.06	high	yes	0	3.33
**18**	359.39	4	1	4	27.63	3.23	high	yes	1	3.55
**19**	359.39	4	1	4	27.63	3.30	high	yes	1	3.51
**20**	359.39	4	1	4	27.63	3.26	high	yes	1	3.45
**21**	375.39	5	1	5	36.86	3.44	high	yes	1	3.58
**22**	375.39	5	1	5	36.86	3.43	high	yes	0	3.50
**23**	375.39	5	1	5	36.86	3.42	high	yes	0	3.44
**24**	427.39	7	1	5	27.63	3.56	low	no	1	3.66
**25**	377.38	5	1	4	27.63	3.28	high	yes	1	3.64
**26**	377.38	5	1	4	27.63	3.26	high	yes	1	3.61
**27**	377.38	5	1	4	27.63	3.16	high	yes	1	3.56

a *M*_W_, molecular weights; HBA, H-bond acceptors; HBD, H-bond donors; NRB, number of rotatable bonds; TPSA, topological polar surface area; GI absorption, gastrointestinal absorption; and BBB permeant, blood–brain barrier.

**Figure 6:**
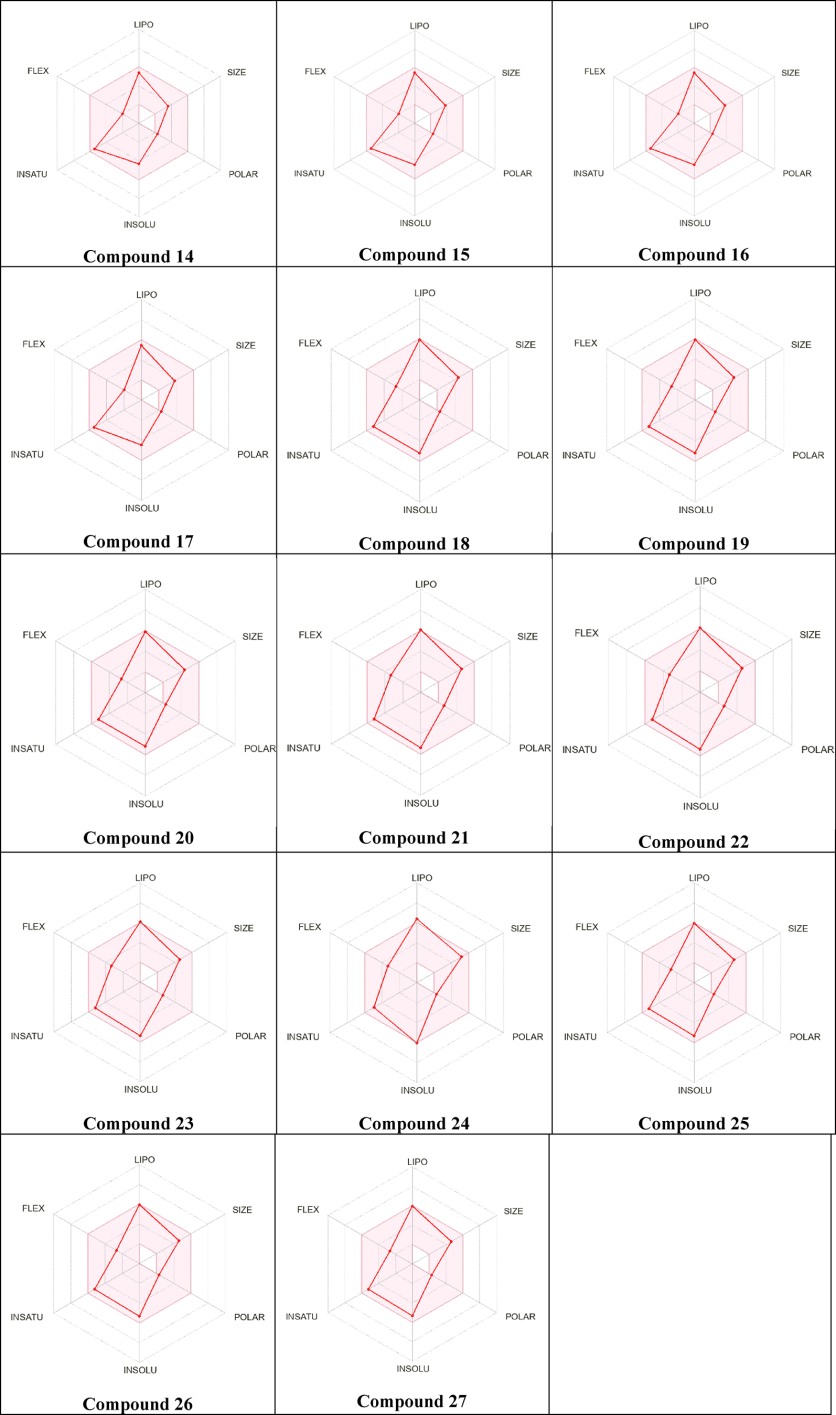
Bioavailability radars of pyrazolines **(14–27)** for drug-likeness properties.

**Figure 7:**
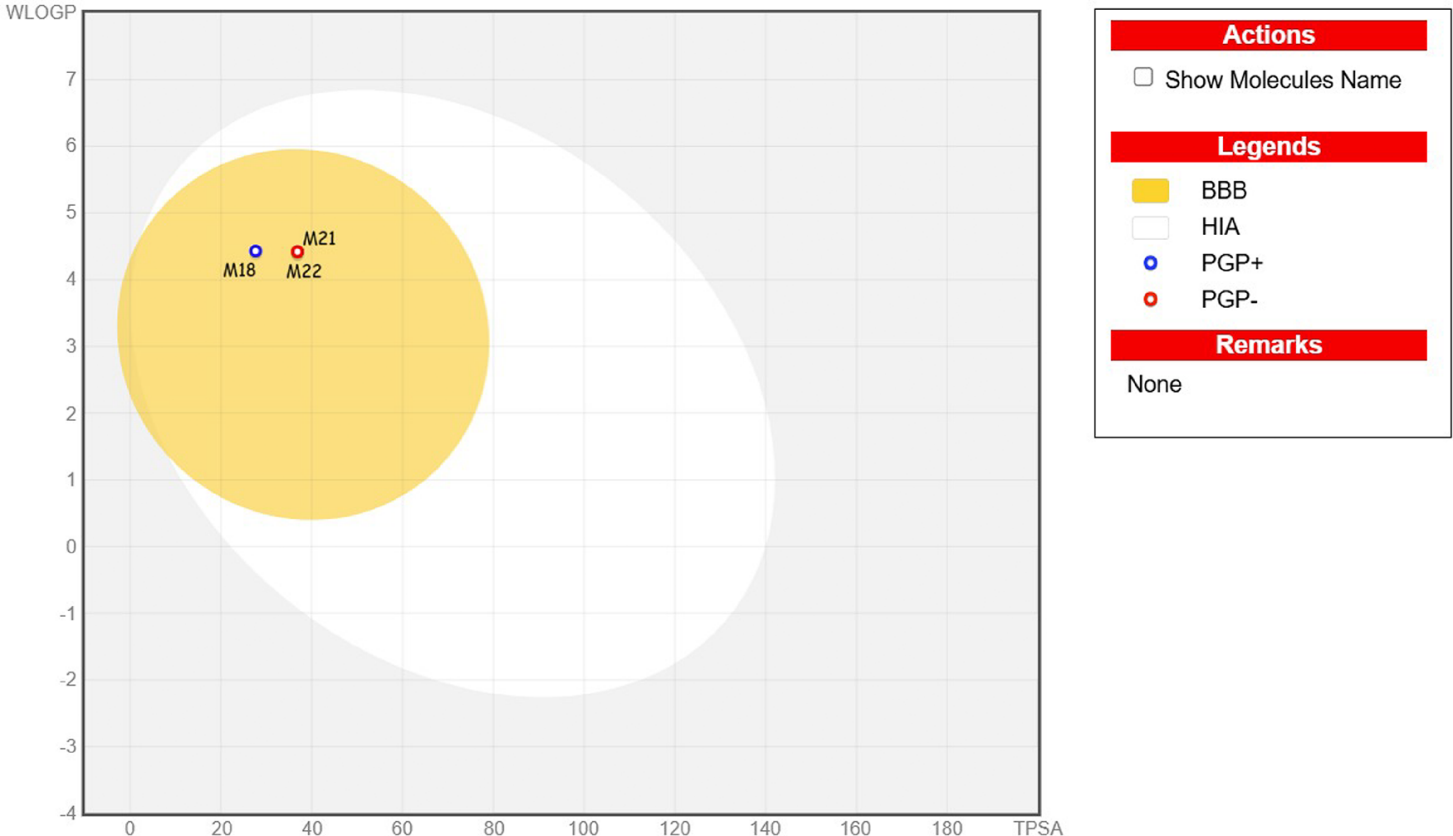
BOILED-Egg plot of the most active pyrazolines (**18**, **21,** and **22**).

## 4 Conclusions

In the present study, novel pyrazoline derivatives, incorporating pyrrolidine and fluorine atoms, were synthesized and their inhibitory effects against α-amylase and α-glucosidase were discussed. Furthermore, the SARs of these derivatives were evaluated. The compounds exhibited weak inhibitory activity against α-amylase while demonstrating selective inhibition of α-glucosidase. Compound **21** was evaluated as the lead compound due to its potency in inhibiting α-glucosidase being twice that of the reference drug acarbose. Molecular docking studies have determined the binding interactions and binding affinities between α-glucosidase and the compounds. The *in silico* data demonstrated the capacity of compounds **18**, **21,** and **22** to bind to the active site of α-glucosidase enzyme with high specificity, with the potential to exhibit inhibitory effects through various interactions with basic residues. Additionally, MD simulations of active compounds (**18**, **21,** and **22**) provided comprehensive information on the structural properties and binding mechanisms of the complexes. Consequently, it is thought that this study will guide the rational design of more potent and selective inhibitors with better therapeutic efficacy for the treatment of diabetes.

## Supplementary Material



## Notes

^1^H NMR; ^13^C NMR; FT-IR; and mass spectrum of compounds **14–27** (PDF)
