# Supplementary material for: Novel Pyrrolidine-Based Pyrazolines as α‑Glucosidase Inhibitors: Microwave-Assisted Synthesis, Antidiabetic Activity, In Silico ADMET Prediction, Molecular Docking, and Molecular Dynamics Simulations
Source: ACS Omega. 2025 Sep 22;10(39):45450–64. doi: 10.1021/acsomega.5c05455 (PMC12509173; doi:10.1021/acsomega.5c05455)
Supplement: Supplementary file 1 [file ao5c05455_si_001.pdf]

SUPPLEMENTARY DATA

Title: Novel pyrrolidine-based pyrazoles for α-amylase and α-glucosidase inhibitors: Synthesis, *in silico* prediction of ADMET and molecular docking

Bedriye Seda Kurşun Aktar[a], Yusuf Sıcak[b], Abdulkadir Bakırdöven[a*], Gizem Tatar Yılmaz[c,d,e], Süleyman Kaya[c], Ayşegül Karaküçük-İyidoğan[f], Emine Elçin Oruç-Emre[f]

[a]Department of Hair Care and Beauty Services, Yeşilyurt Vocational School, Malatya Turgut Özal University, Malatya, Türkiye

[b]Department of Medicinal and Aromatic Plants, Köyceğiz Vocational School, Mugla Sitki Kocman University, Köyceğiz, Muğla, Türkiye

[c]Department of Biostatistics and Medical Informatics, Faculty of Medicine, Karadeniz Technical University, Trabzon, Türkiye

[d]Department of Bioinformatics, Institute of Health Sciences, Karadeniz Technical University, Trabzon, Türkiye

[e]Yılmaz Bilişim R&D Consulting Software Engineering and Services Trade Limited Company, Trabzon, Türkiye

[f]Department of Chemistry, Faculty of Arts and Sciences, Gaziantep University, Gaziantep, Türkiye

***Correspondence Author:**

Bedriye Seda Kurşun Aktar

Department of Hair Care and Beauty Services, Yeşilyurt Vocational School, Malatya Turgut Özal University, Malatya, Türkiye

Phone number: +90 252 211 32 58

Fax number: +90 252 211 50 41

E-mail: bseda.kursunaktar@ozal.edu.tr

General Procedure for the Synthesis of Pyralozines (14-27)

1 mmol of (*E*)-3-[substituted phenyl]-1-[4-(pyrrolidin-1-yl)phenyl]prop-2-en-1-one **1-13** [37] was dissolved in methanol. To this solution, 1 mmol of hydrazine monohydrate and a few drops of acetic acid were added. The reaction mixture was then subjected to microwave irradiation at 70 °C and 700 W for 15 minutes. The progress of the reaction was monitored by thin-layer chromatography (TLC), and upon completion, the mixture was poured onto ice to terminate the reaction. The resulting crude product was collected by filtration and purified by recrystallization from methanol [40].

Determination of α-amylase inhibitory activity

The α-amylase inhibitory activity of compounds **14-27** was evaluated using a spectrophotometric method, with slight modifications from previously established protocols [42]. Briefly, 25 μL of the sample solution at various concentrations was mixed with 50 μL of α-amylase solution (0.1 U/mL) in a phosphate buffer (20 mM, pH 6.9, prepared with 6 mM NaCl) in a 96-well microplate. This mixture was pre-incubated at 37 °C for 10 min. Following the pre-incubation, 50 μL of a starch solution (0.05%) was added, and the reaction mixture was incubated for another 10 min at 37 °C. The reaction was then terminated by adding 25 μL of HCl (0.1 M), followed by the addition of 100 μL of Lugol's solution for monitoring. Absorbance was measured at 565 nm using a 96-well microplate reader.

2.3.2. Determination of α-glucosidase inhibitory activity

The α-glucosidase inhibitory activity of compounds **14-27** was determined using a spectrophotometric method, with slight modifications from previously reported methods [43]. Specifically, 50 μL of phosphate buffer (10 mM, pH 6.9), 25 μL of *p*-nitrophenyl-α-D-glucopyranoside in phosphate buffer (10 mM, pH 6.9), 10 μL of sample solution, and 25 μL of α-glucosidase (0.1 U/mL) in phosphate buffer (10 mM, pH 6.0) were combined in a 96-well microplate. After 20 min of incubation at 37 °C, 90 μL of Na_2CO_3 (100 mM) was added to each well to halt the enzymatic reaction. The absorbance was measured at 400 nm using a 96-well microplate reader.

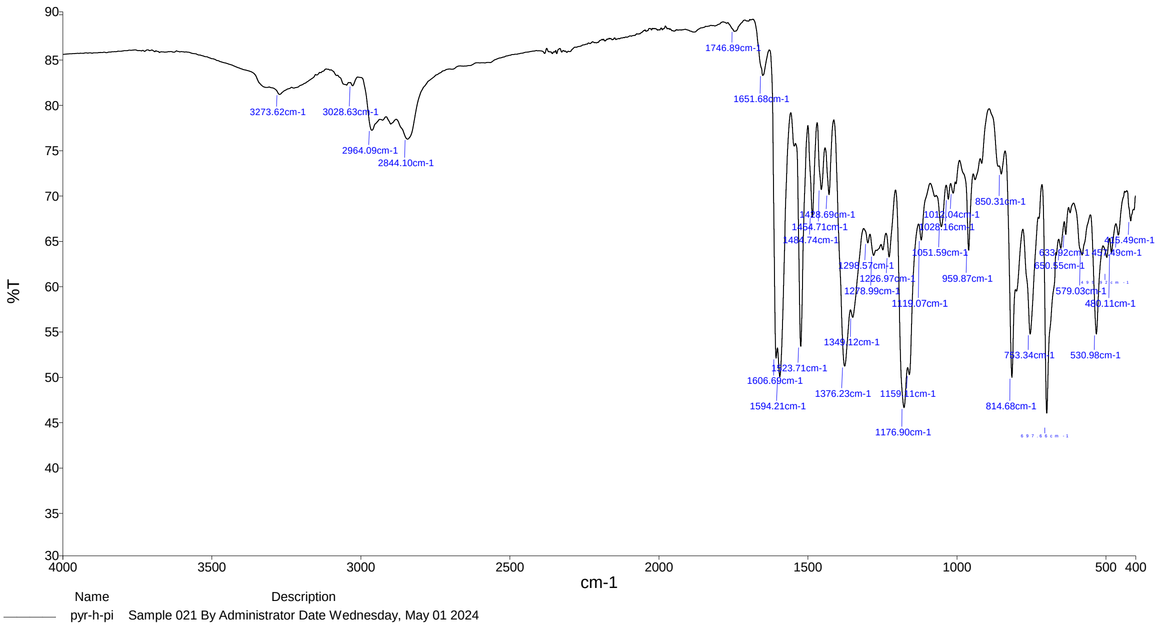

Figure S1. FTIR spectrum of compound 14

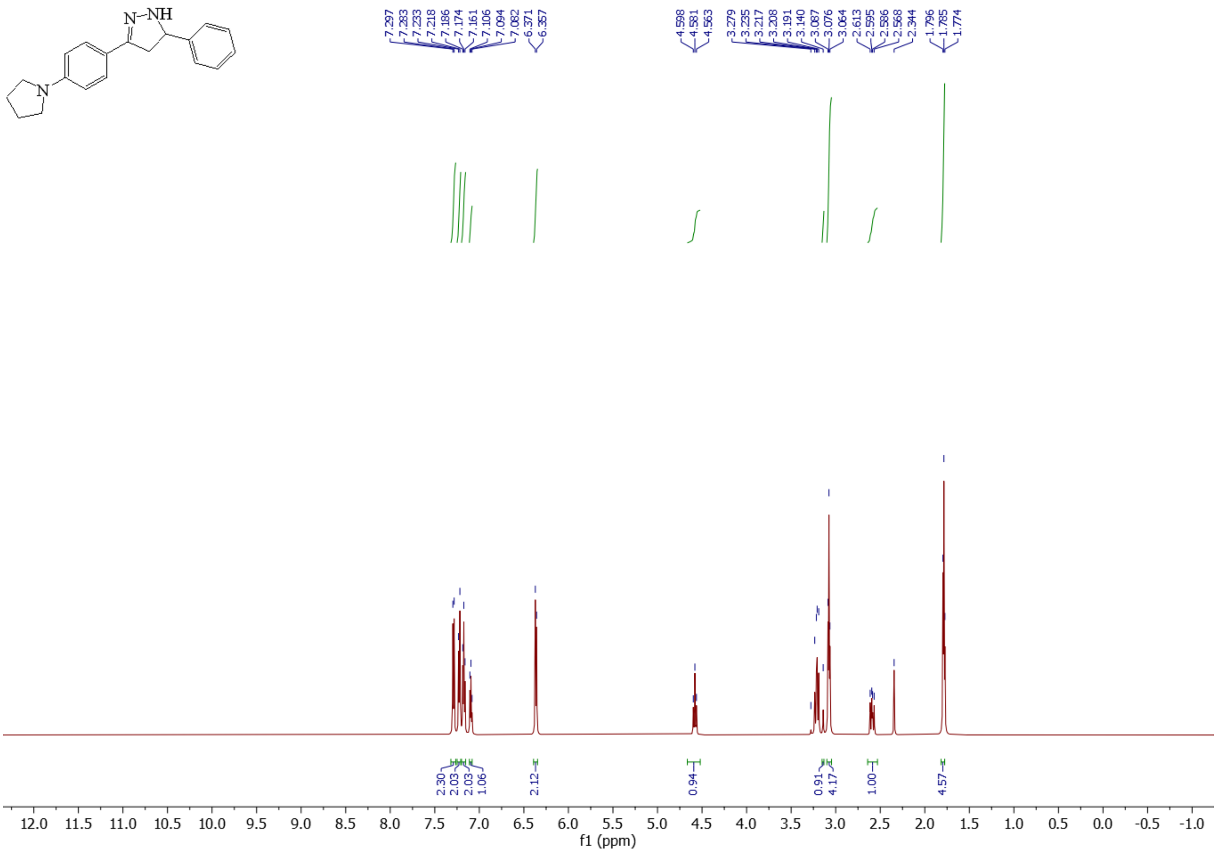

Figure S2. ^{1}H NMR spectrum of compound **14**

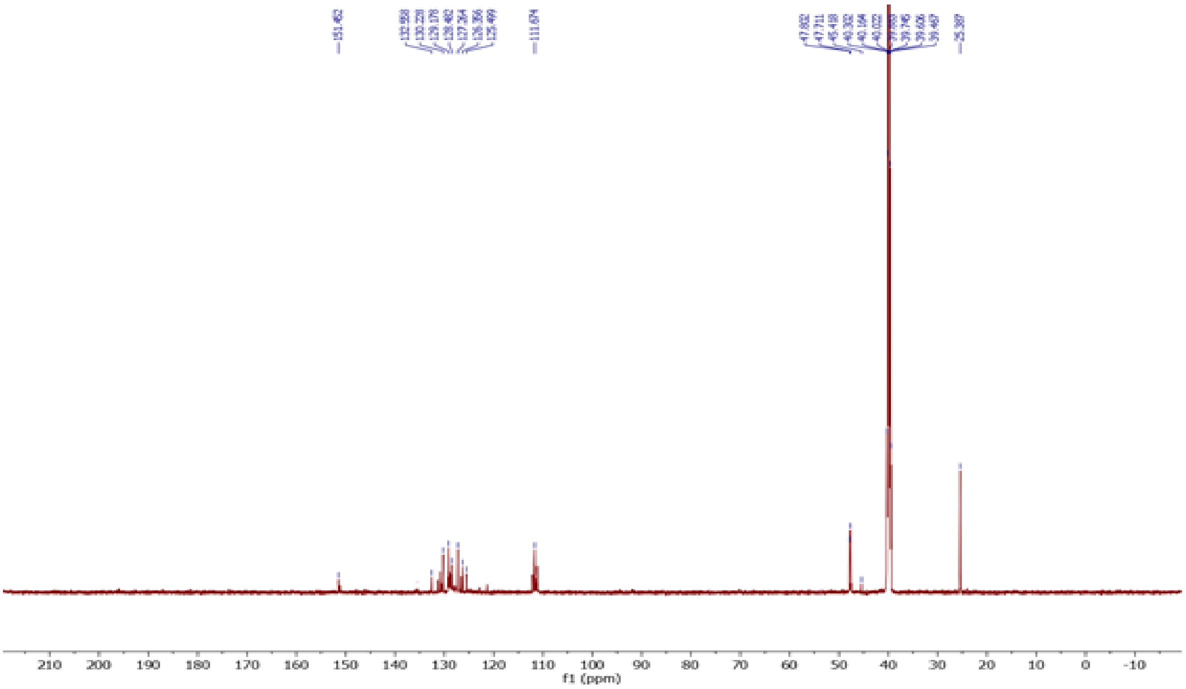

Figure S3. ^{13}C NMR spectrum of compound **14**

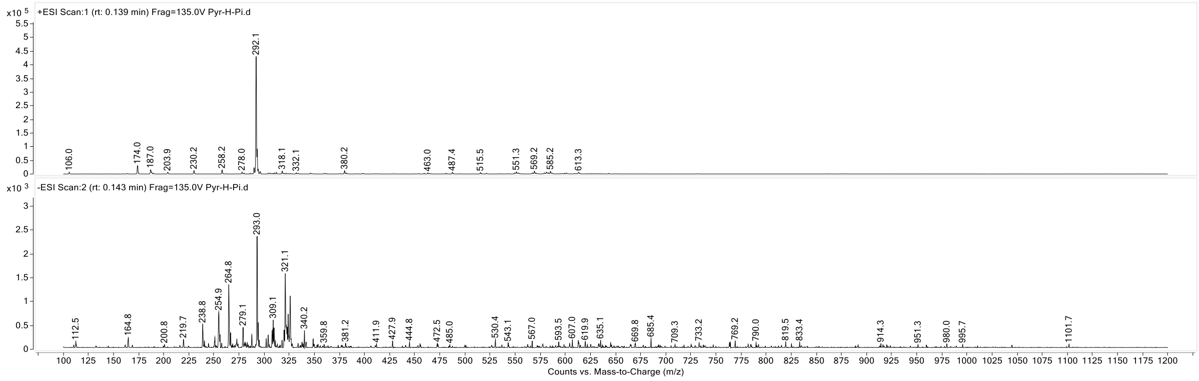

Figure S4. Mass spectrum of compound **14**

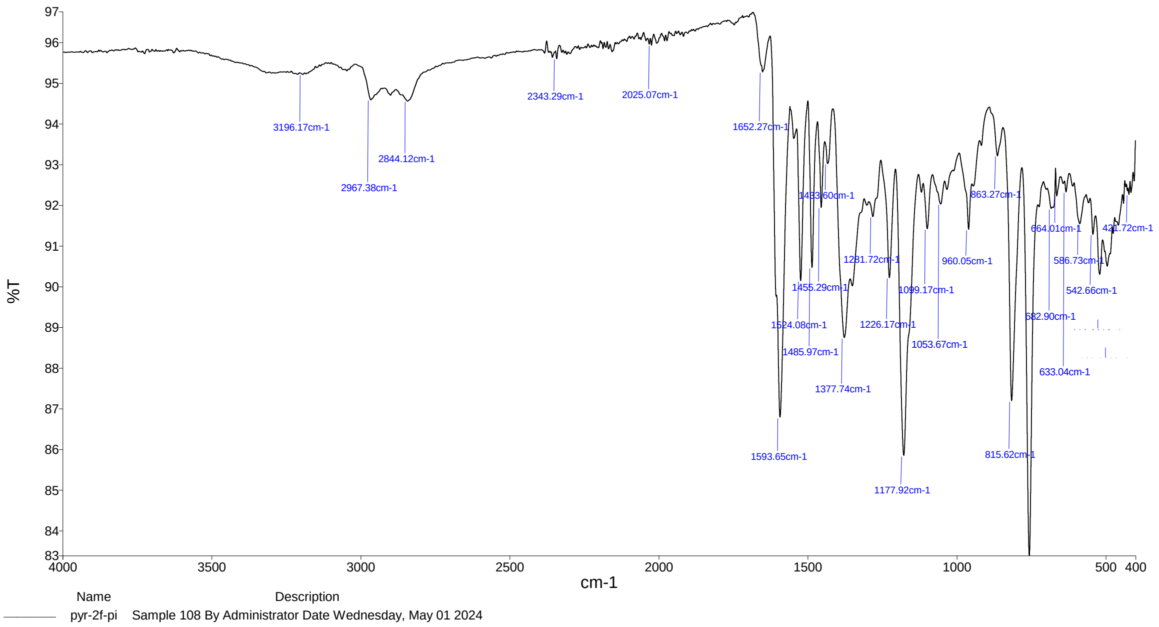

Figure S5. FTIR spectrum of compound 15

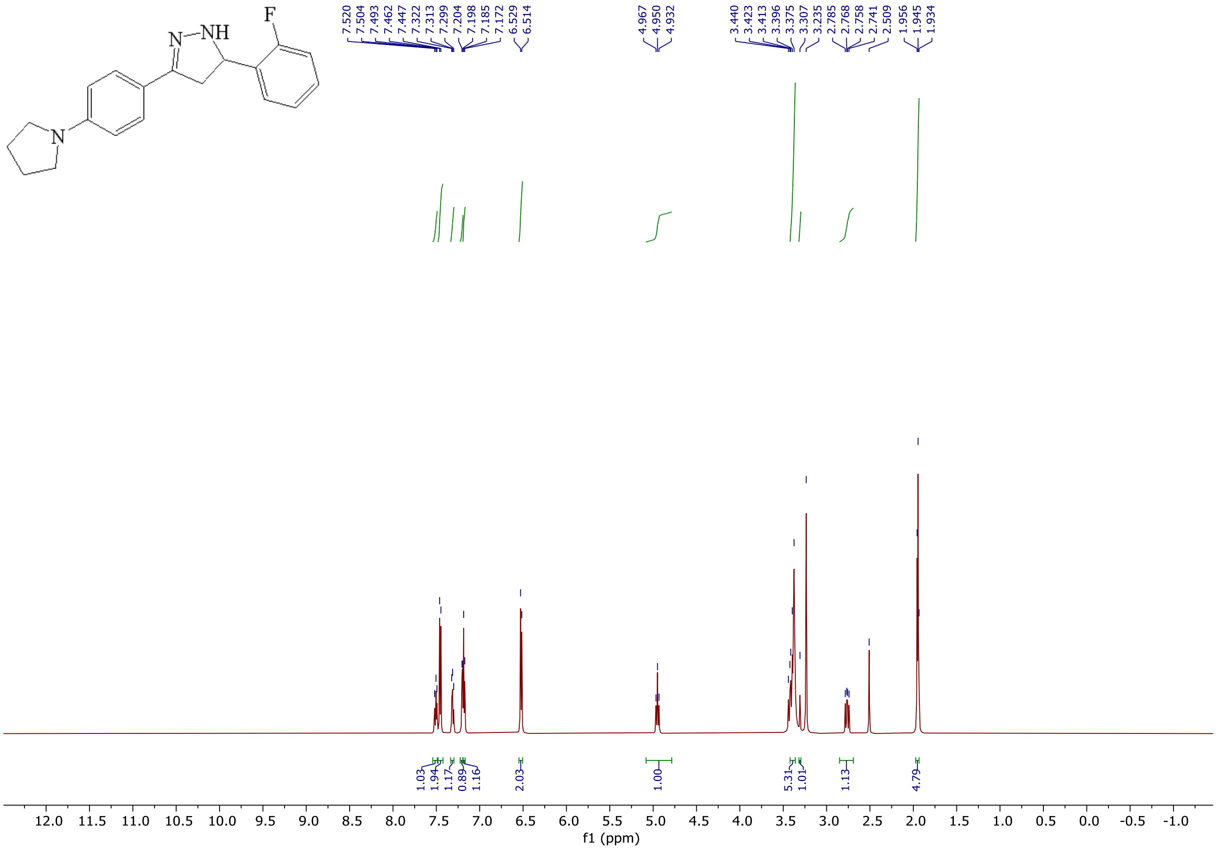

Figure S6. ^{1}H NMR spectrum of compound **15**

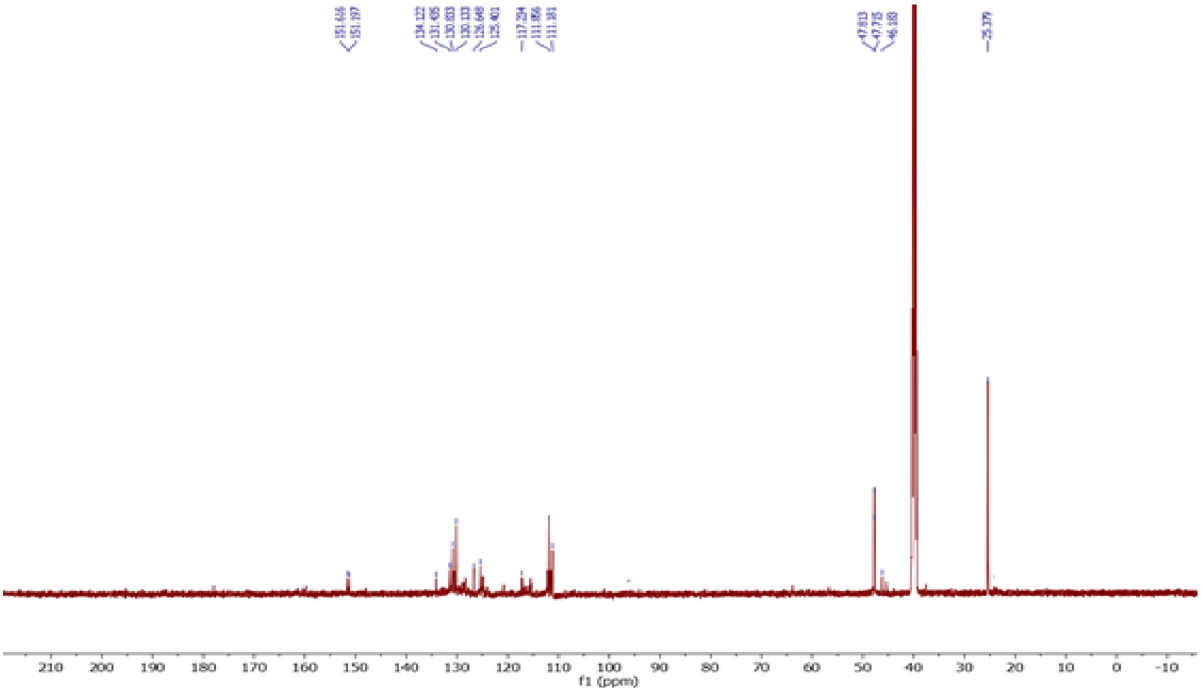

Figure S7. ^{13}C NMR spectrum of compound **15**

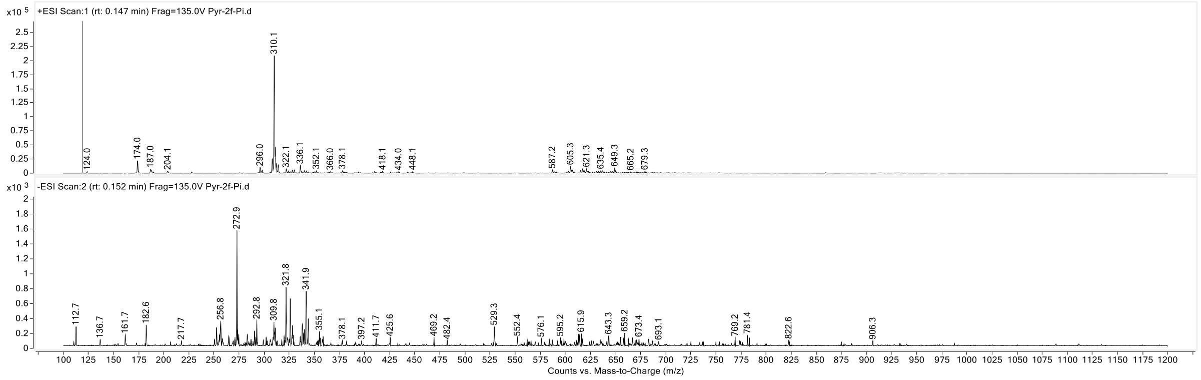

Figure S8. Mass spectrum of compound **15**

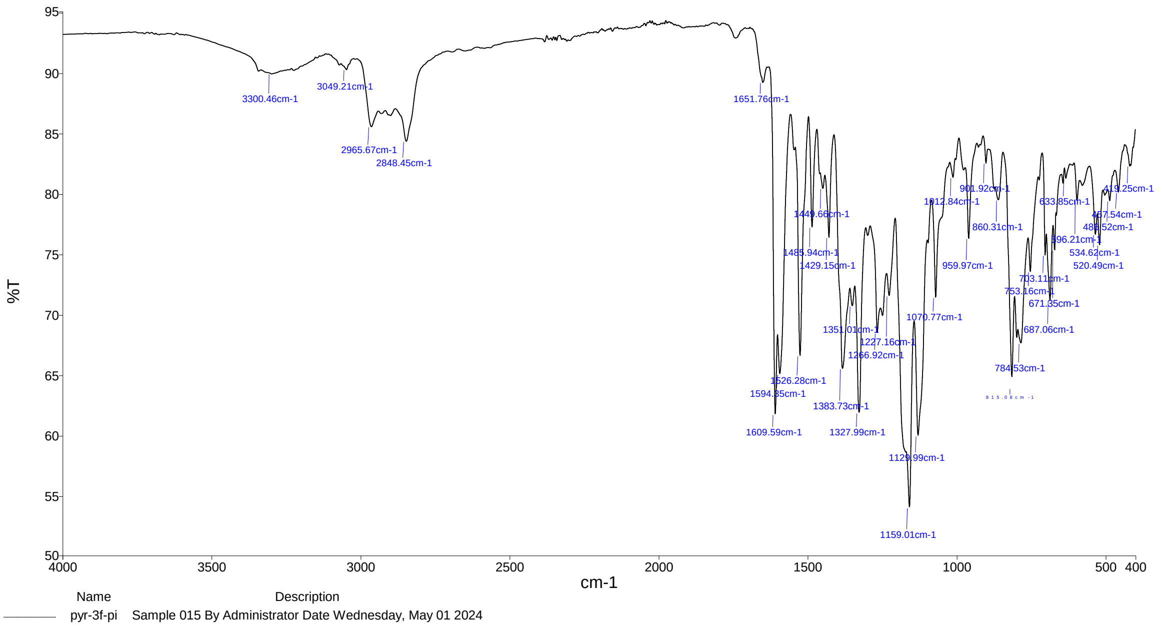

Figure S9. FTIR spectrum of compound **16**

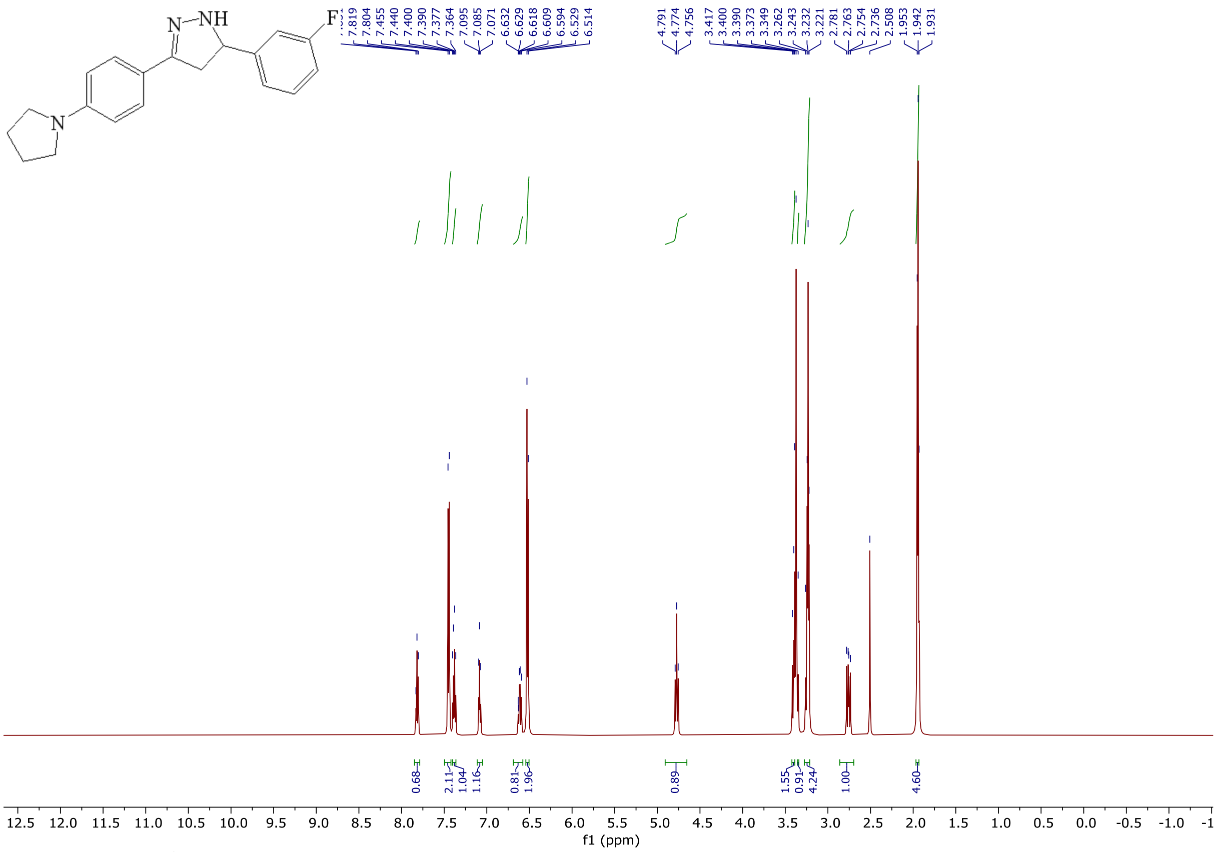

Figure S10. ^{1}H NMR spectrum of compound **16**

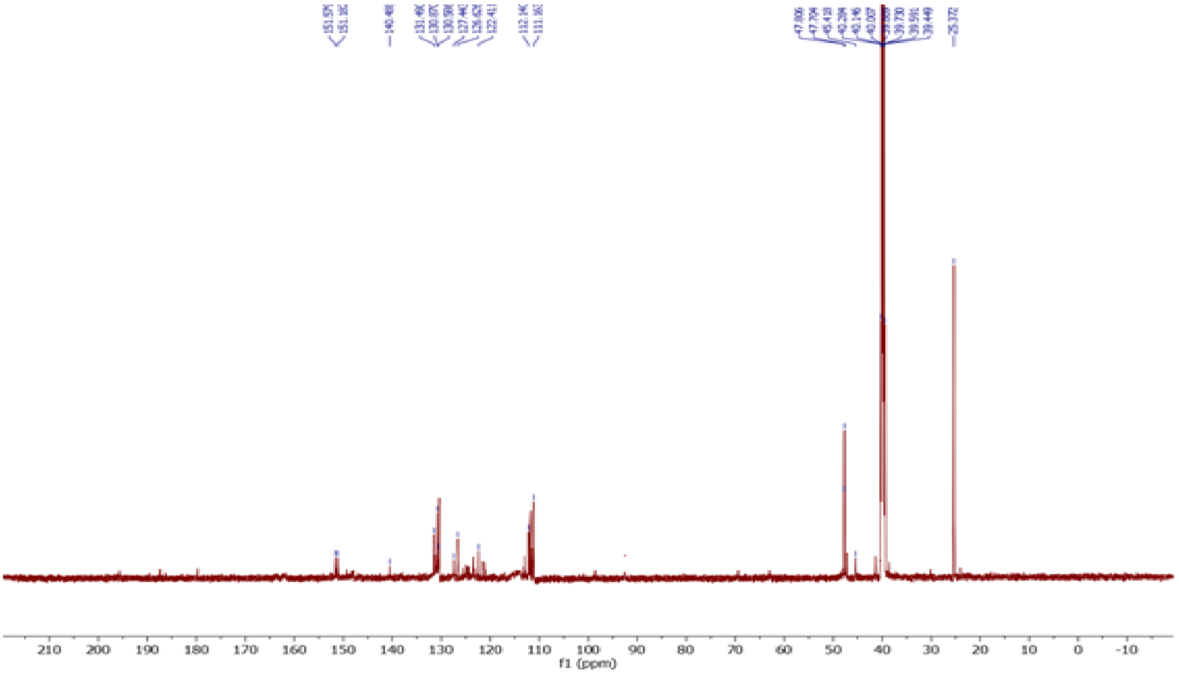

Figure S11. ^{13}C NMR spectrum of compound **16**

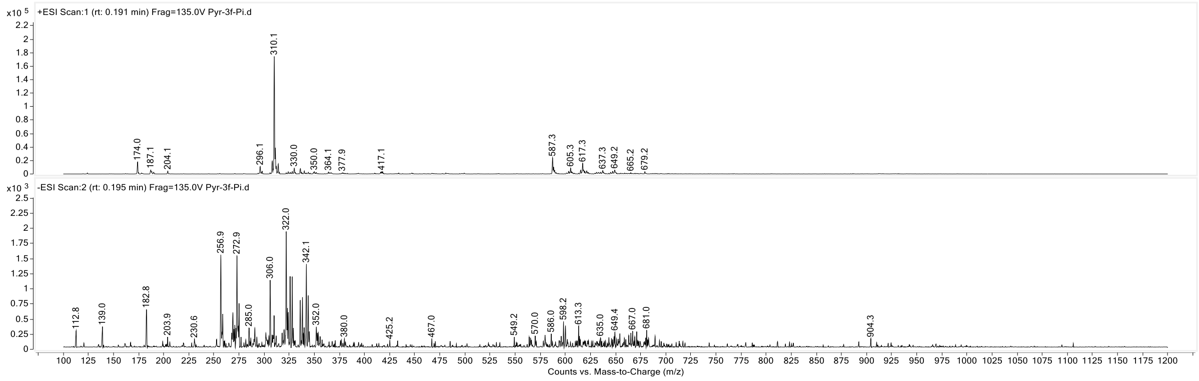

Figure S12. Mass spectrum of compound **16**

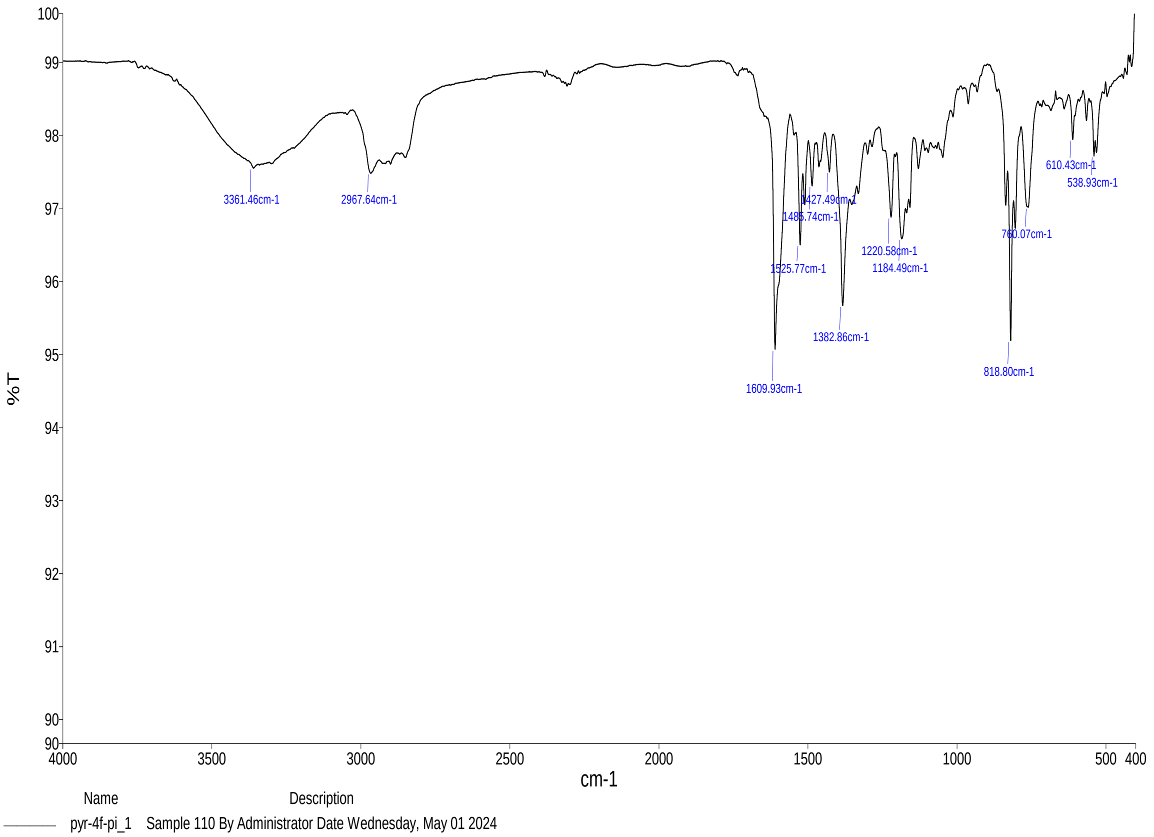

Figure S13. FTIR spectrum of compound **17**

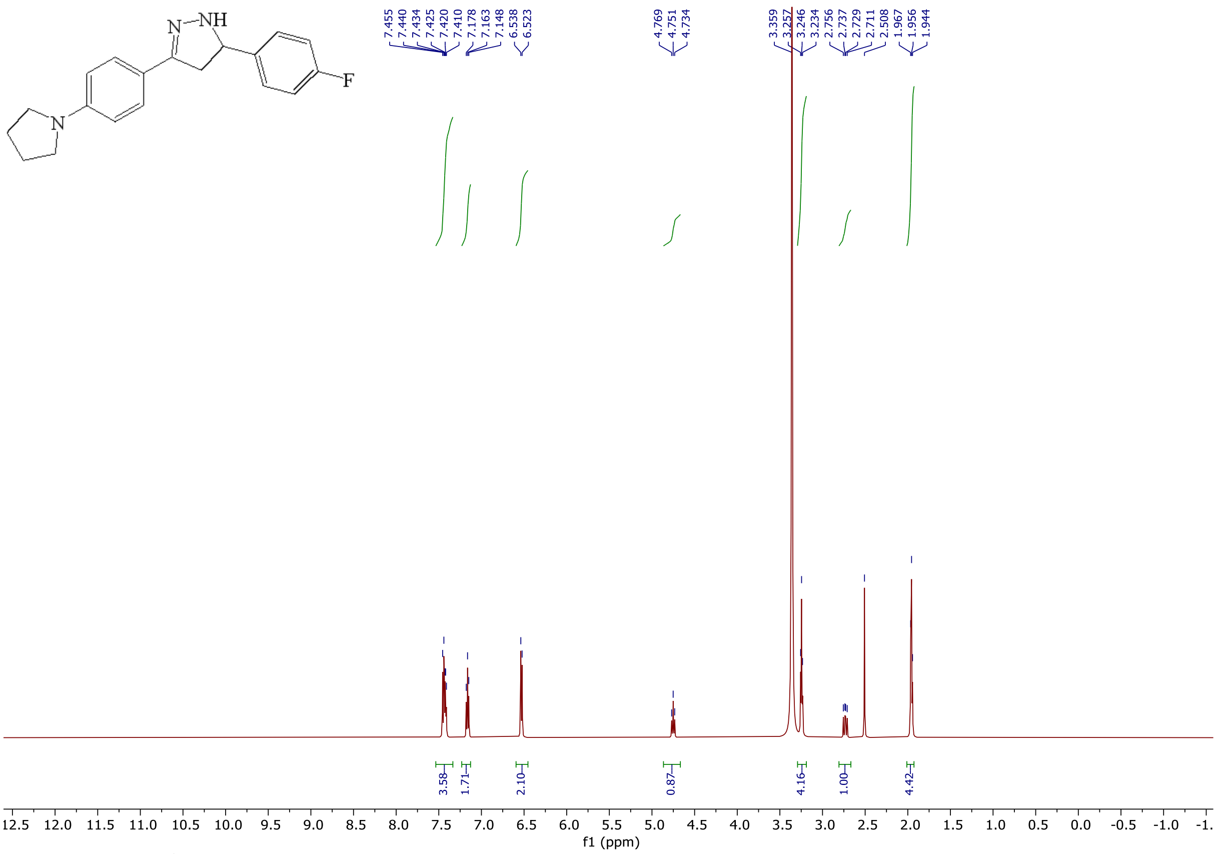

Figure S14. ^{1}H NMR spectrum of compound **17**

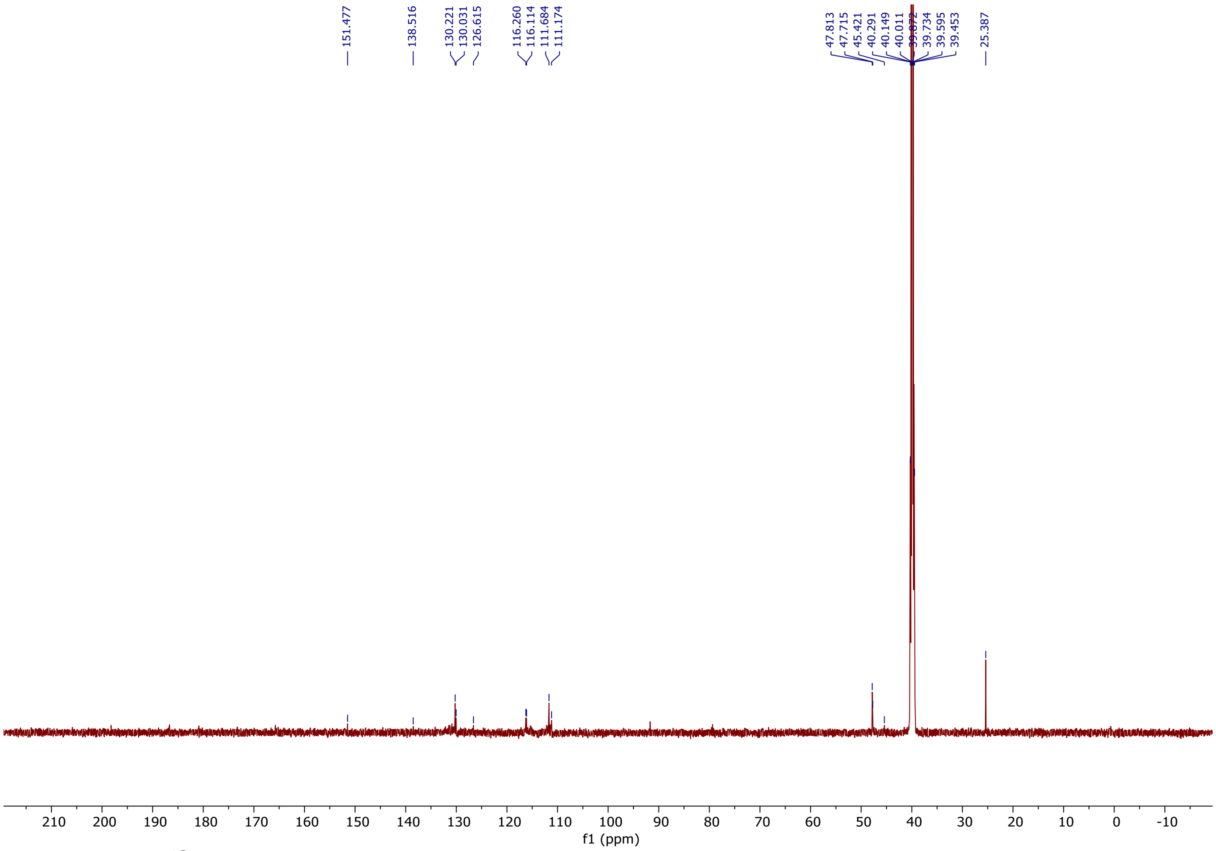

Figure S15. ^{13}C NMR spectrum of compound **17**

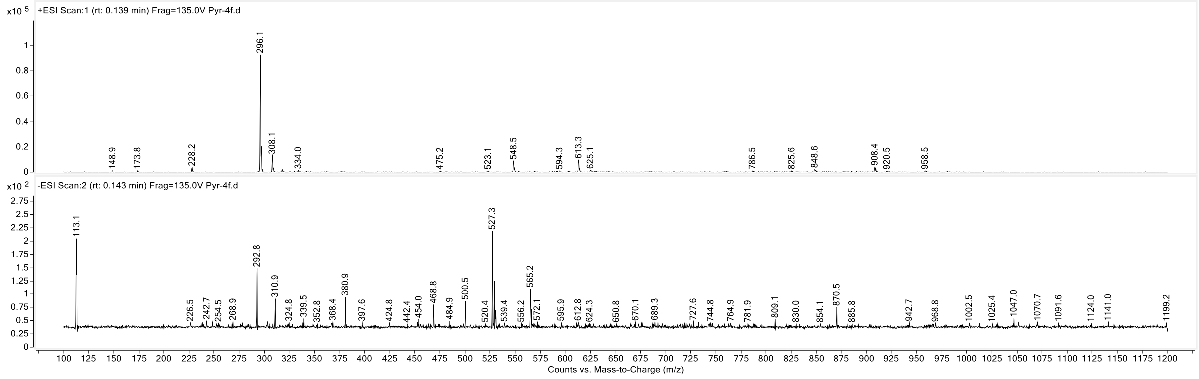

Figure S16. Mass spectrum of compound **17**

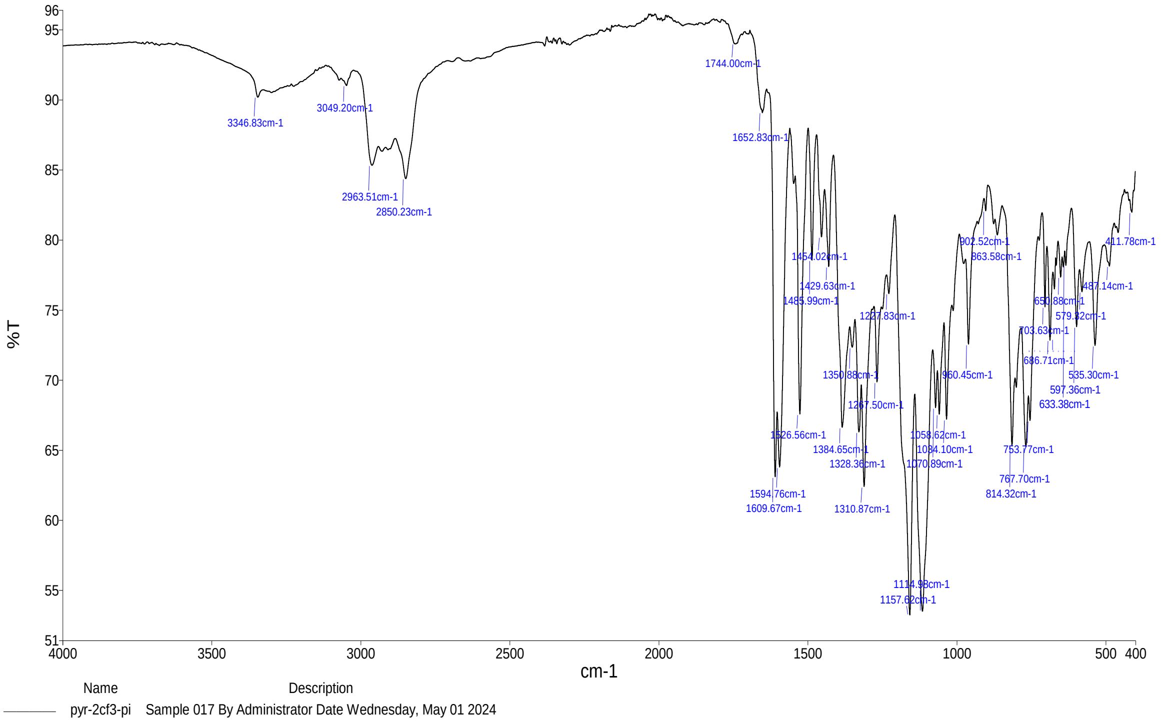

Figure S17. FTIR spectrum of compound **18**

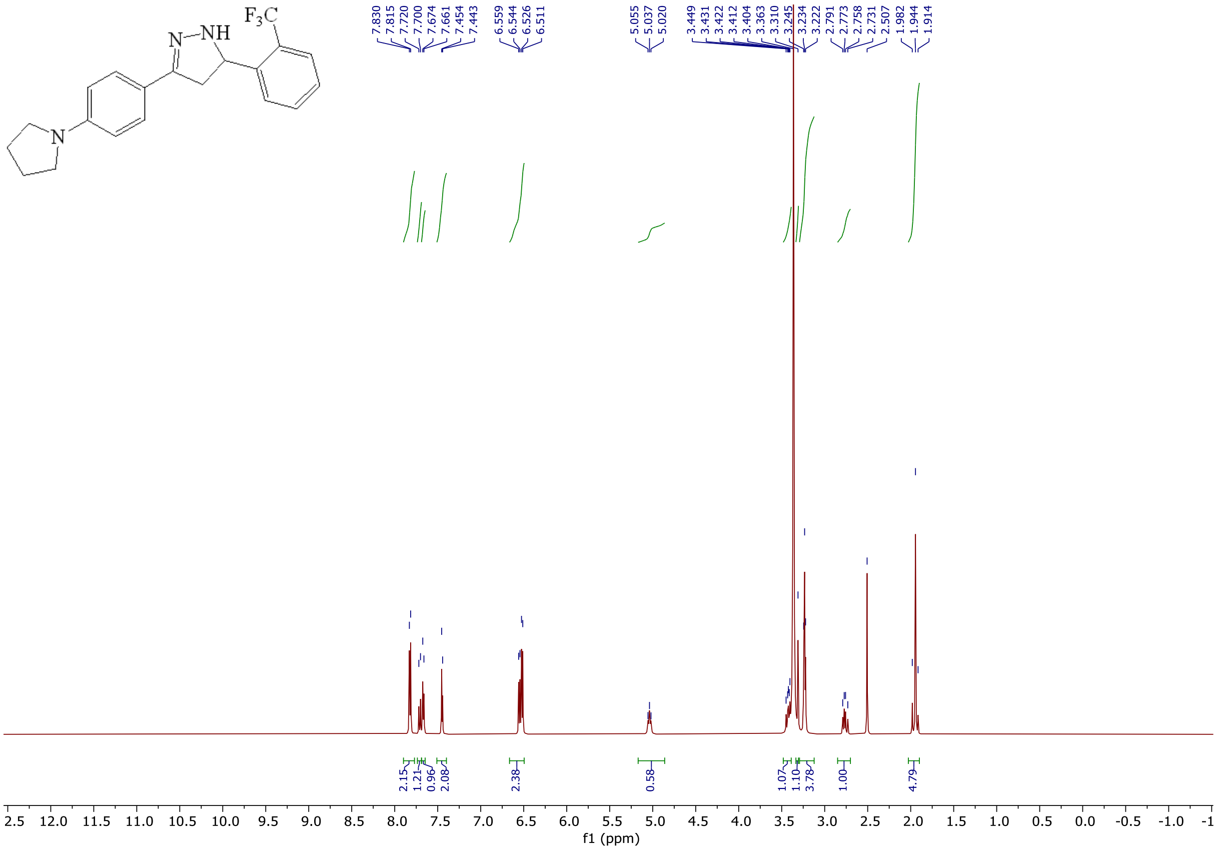

Figure S18. ^{1}H NMR spectrum of compound **18**

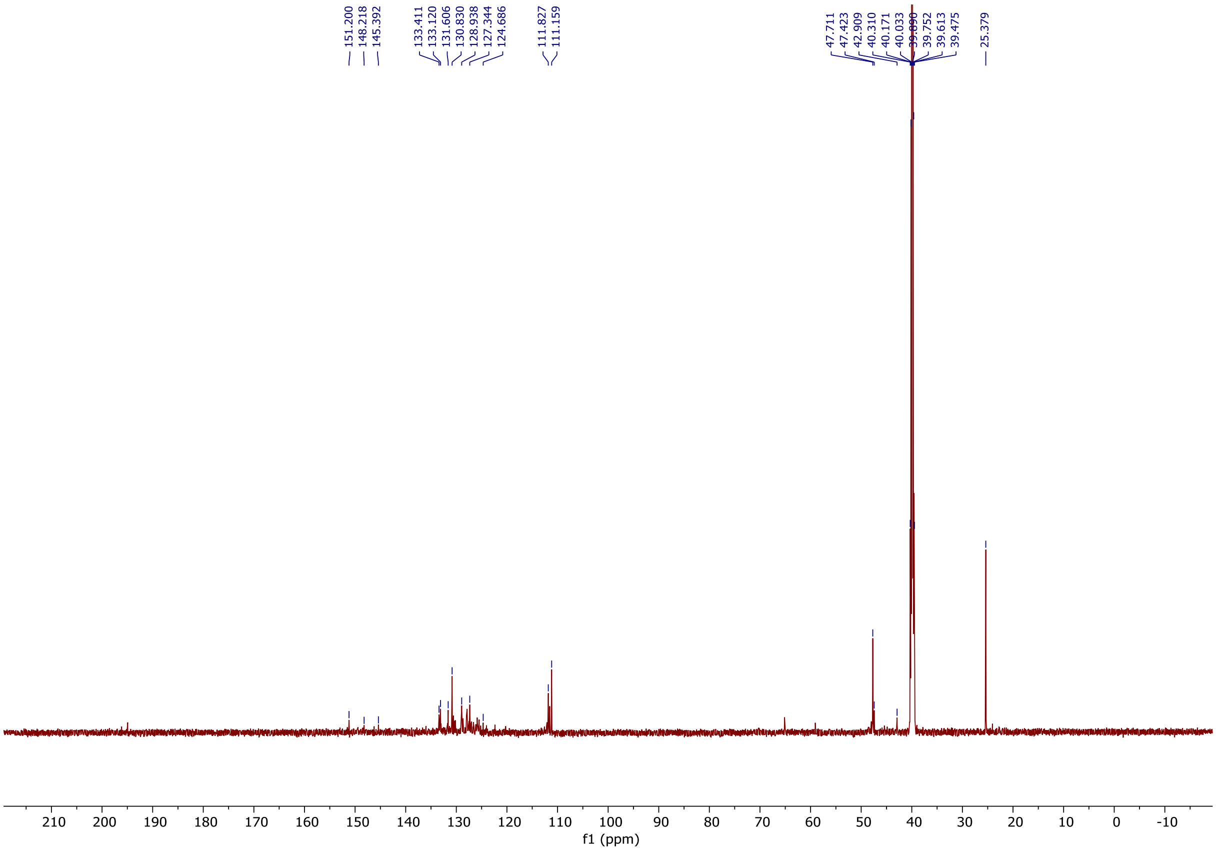

Figure S19. ^{13}C NMR spectrum of compound **18**

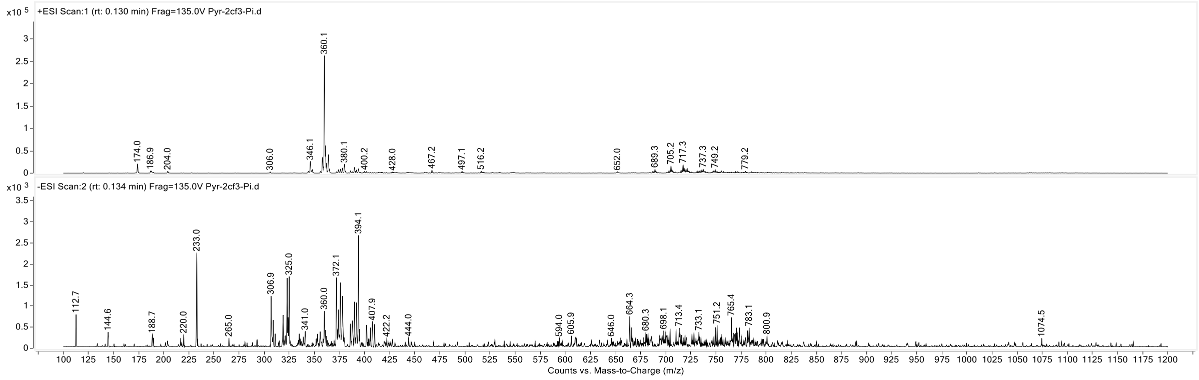

Figure S20. Mass spectrum of compound **18**

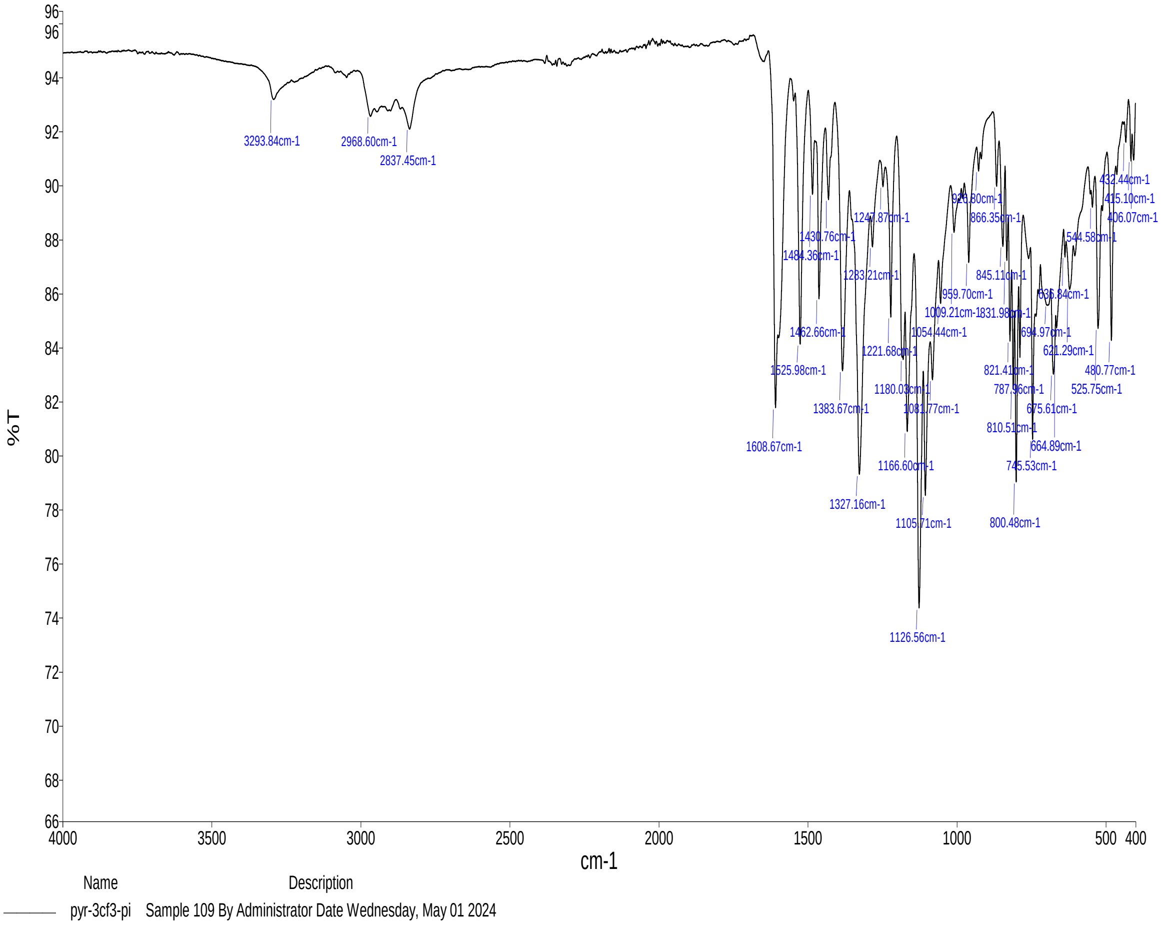

Figure S21. FTIR spectrum of compound **19**

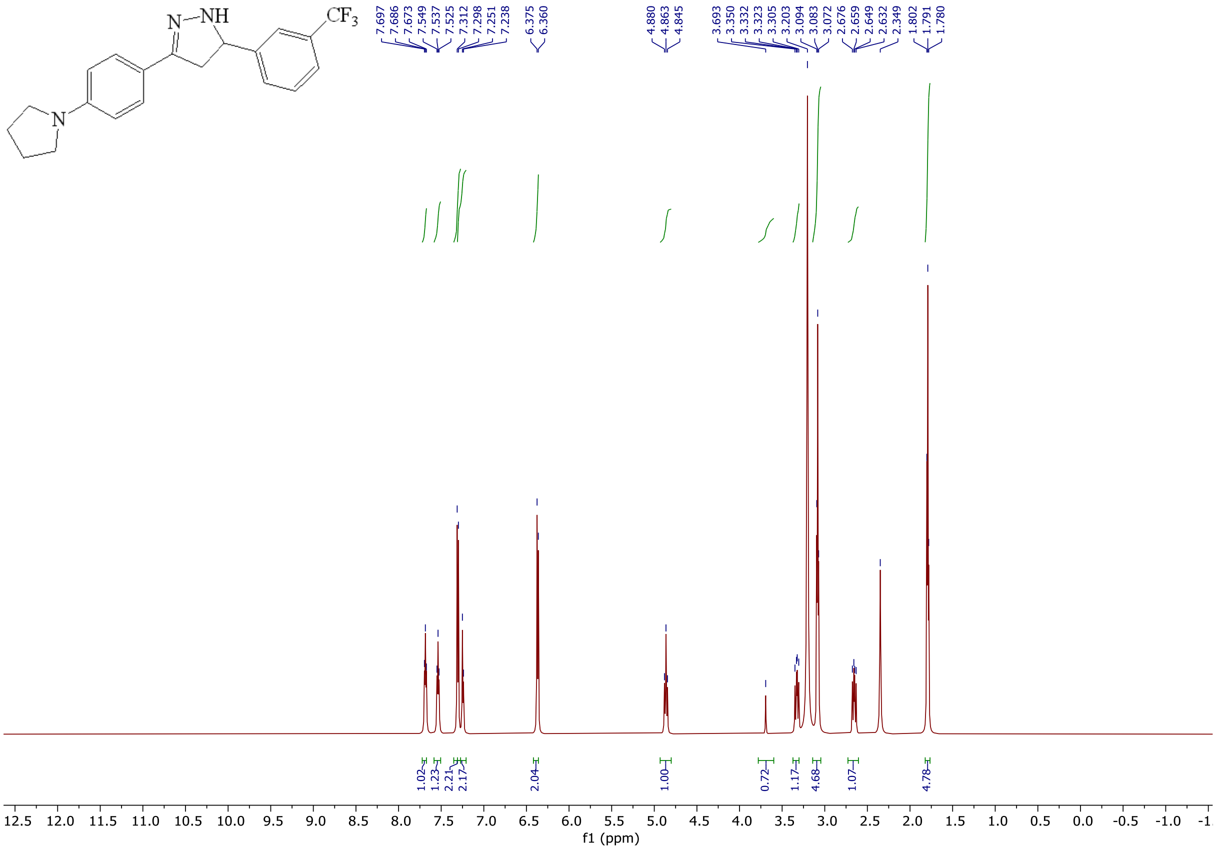

Figure S22. ^{1}H NMR spectrum of compound **19**

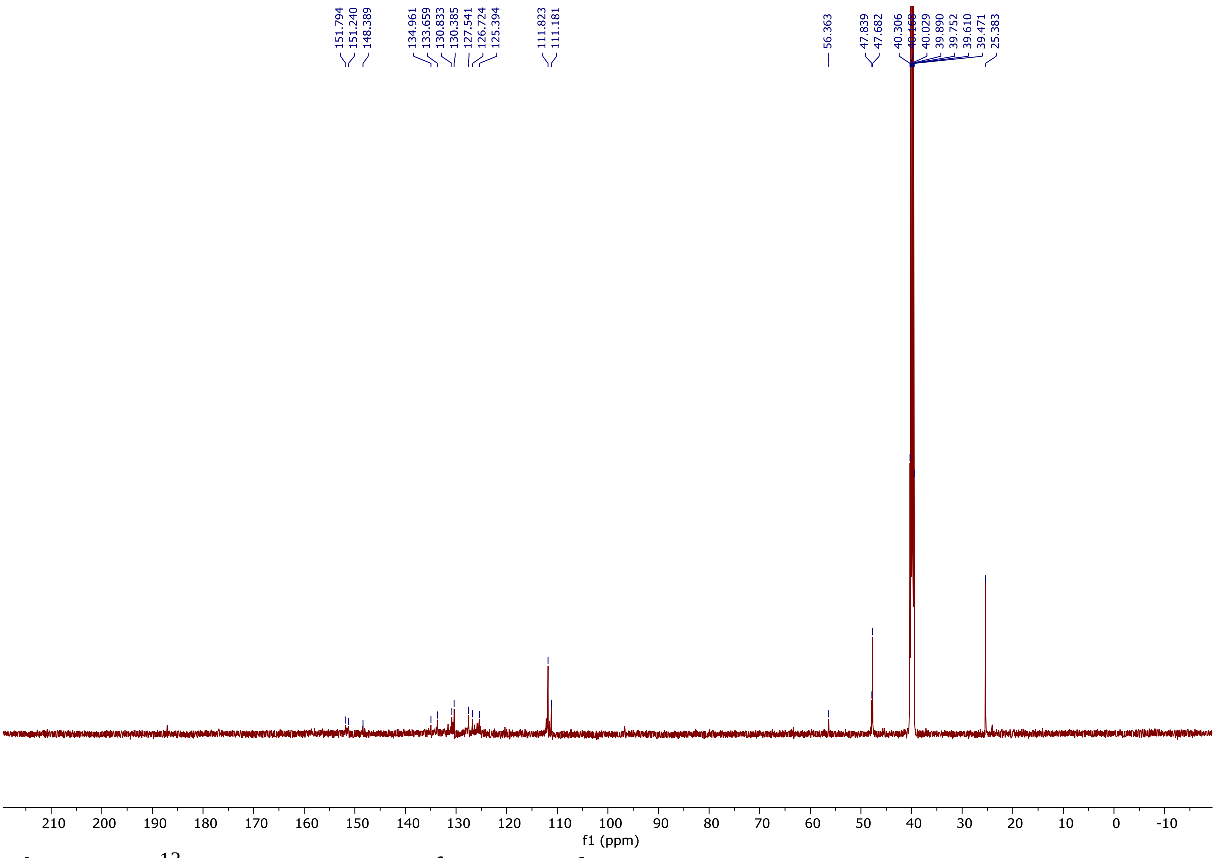

Figure S23 ^{13}C NMR spectrum of compound **19**

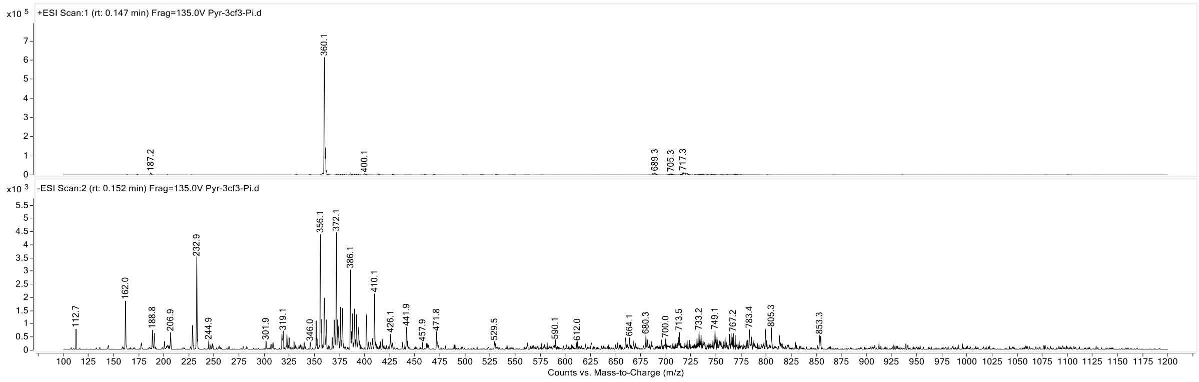

Figure S24. Mass spectrum of compound **19**

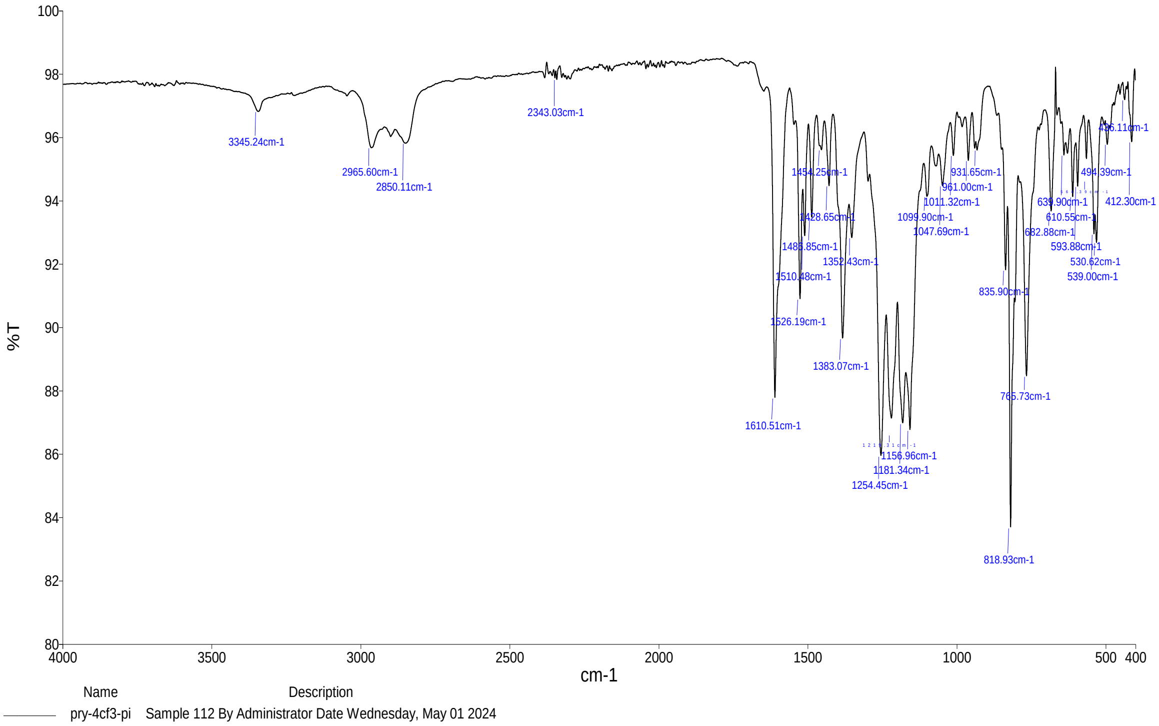

Figure S25. FTIR spectrum of compound **20**

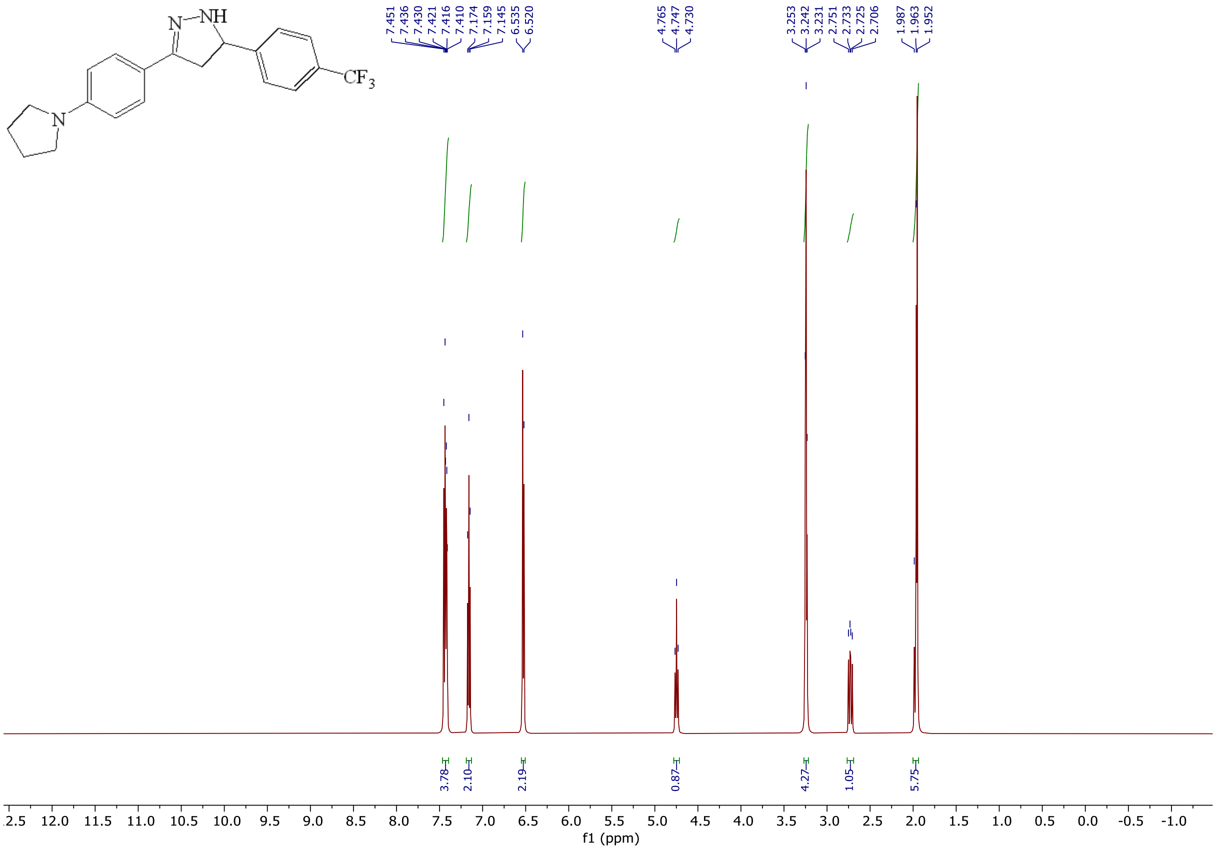

Figure S26. ^{1}H NMR spectrum of compound **20**

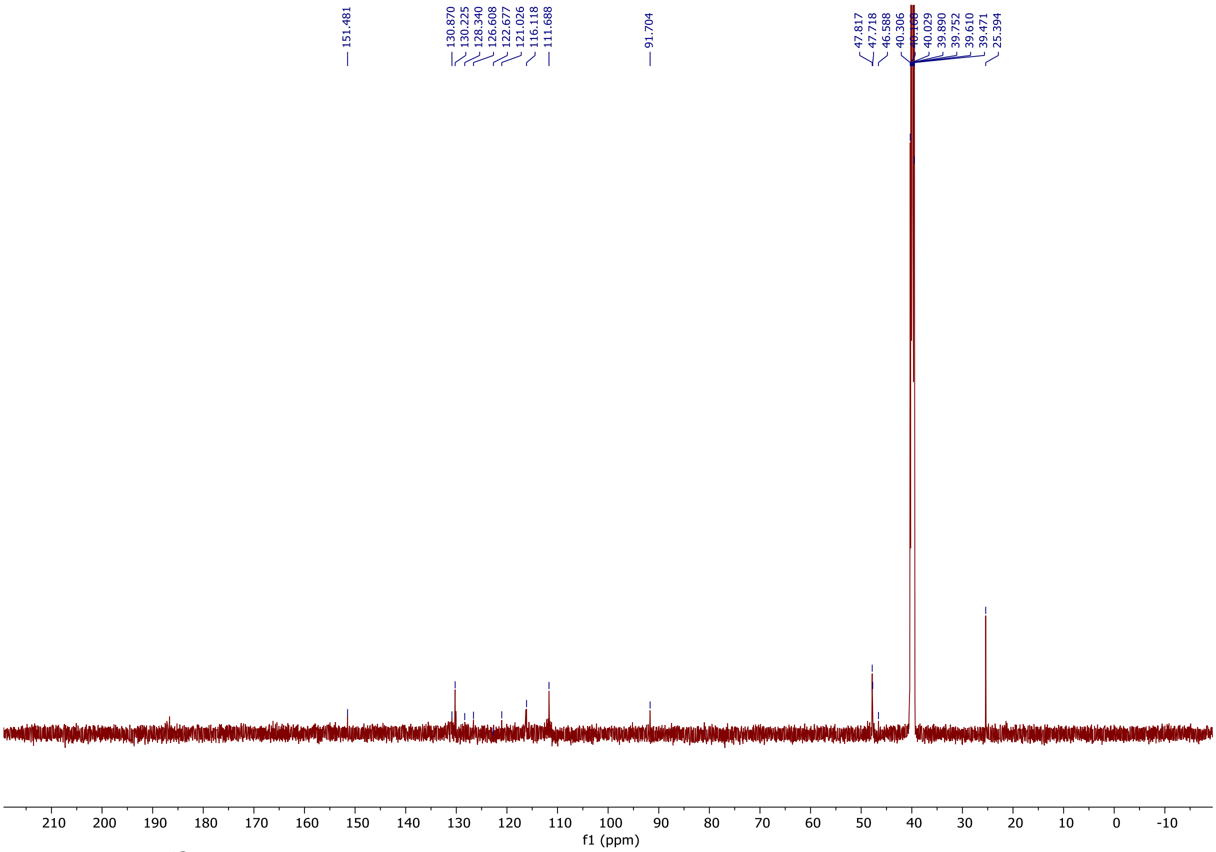

Figure S27. ^{13}C NMR spectrum of compound **20**

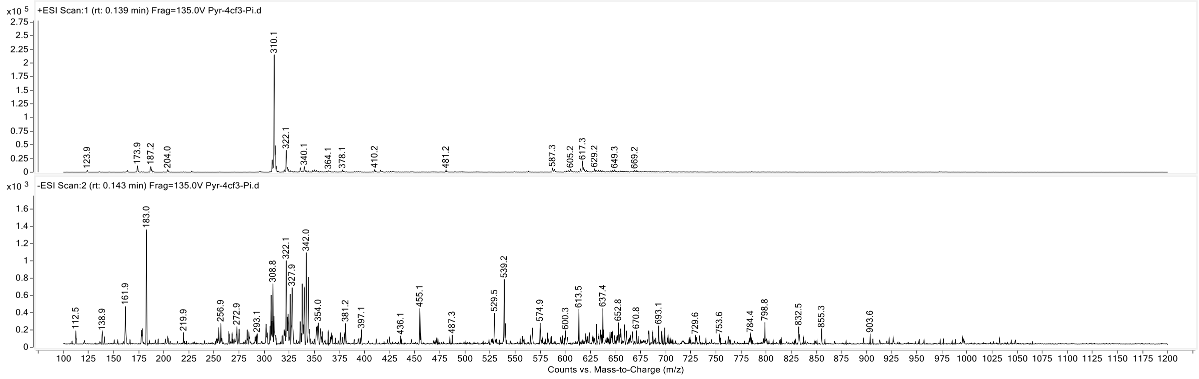

Figure S28. Mass spectrum of compound **20**

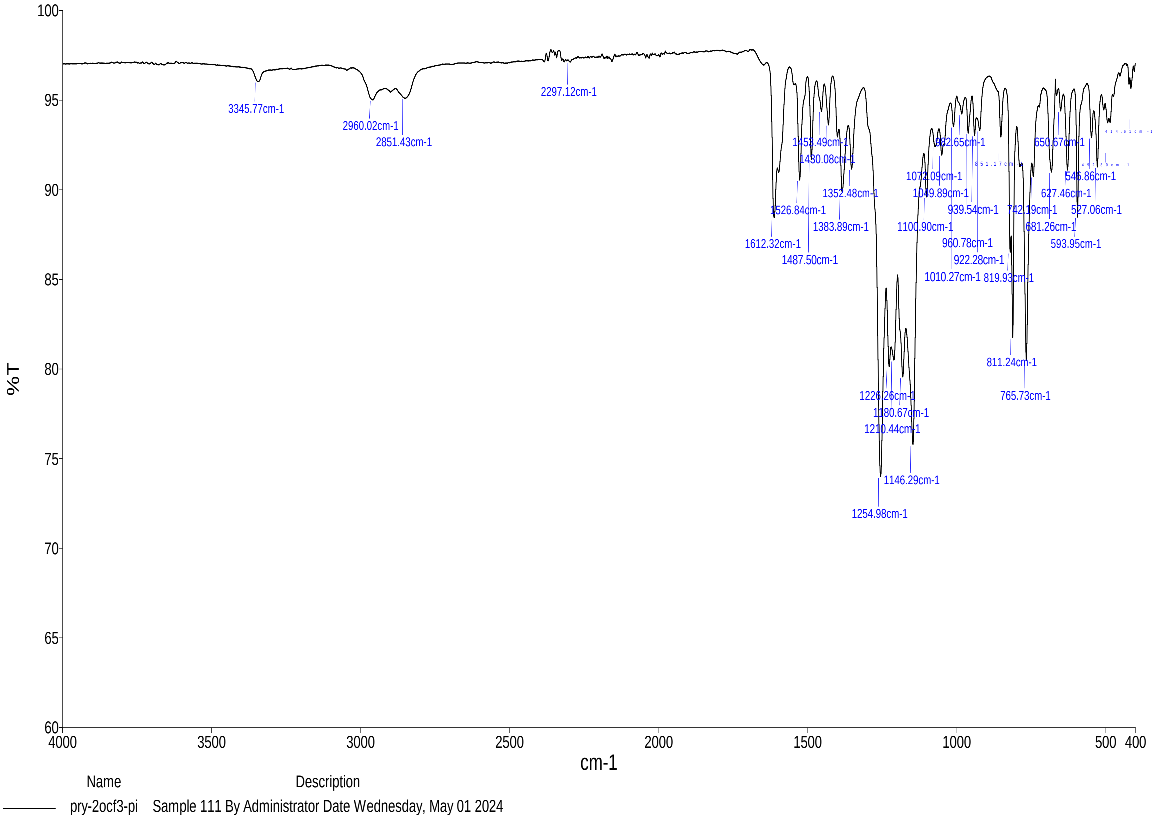

Figure S29. FTIR spectrum of compound **21**

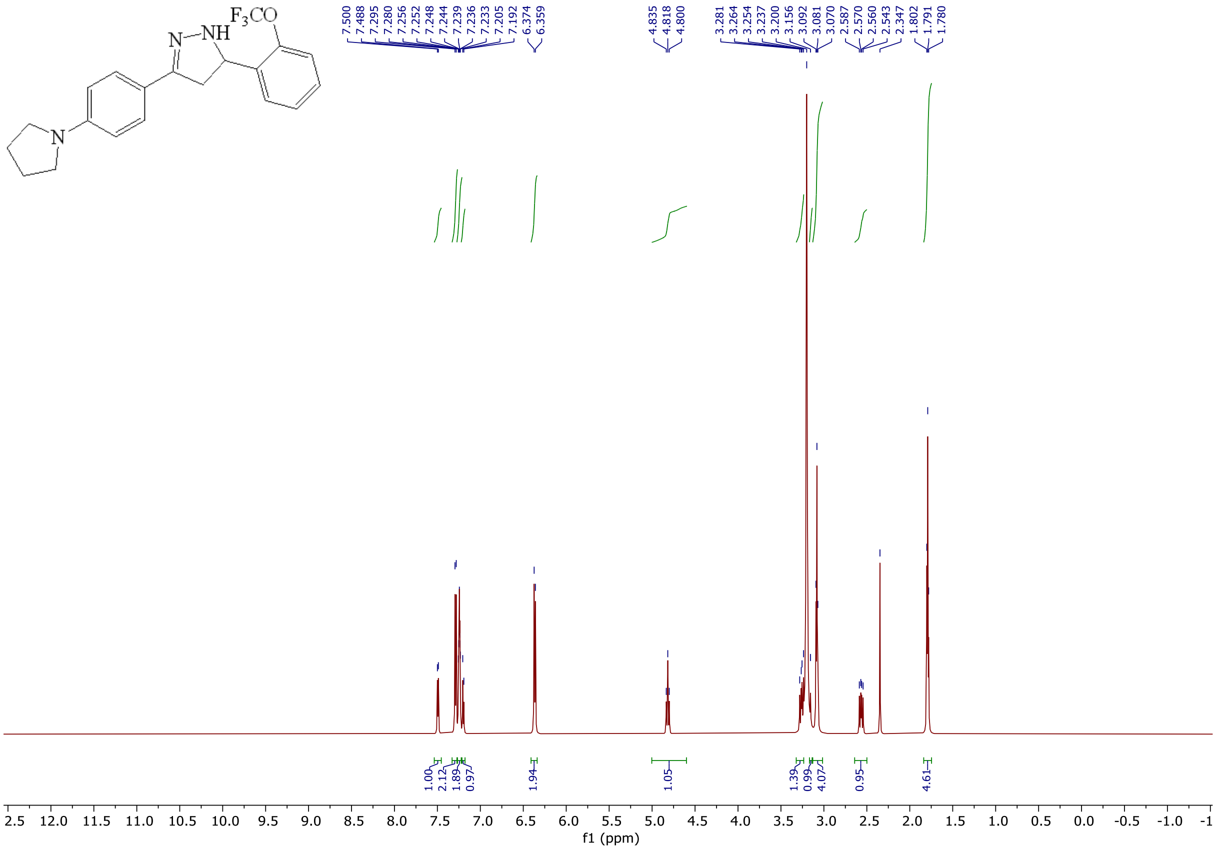

Figure S30. ^{1}H NMR spectrum of compound **21**

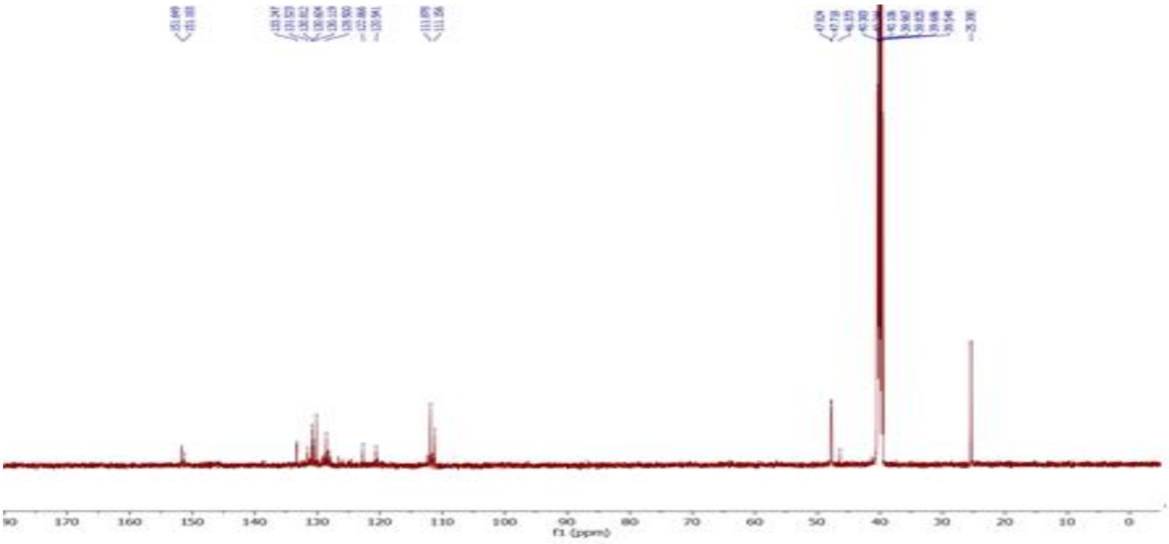

Figure S31^{13}C NMR spectrum of compound **21**

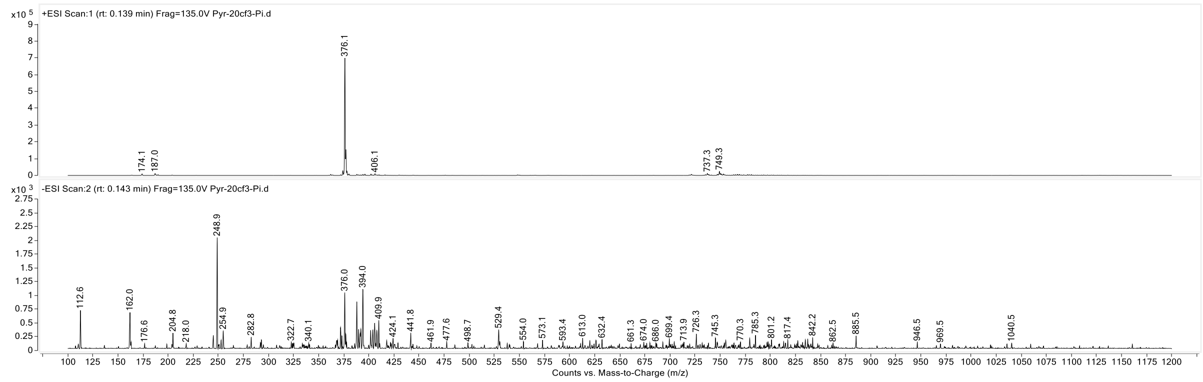

Figure S32. Mass spectrum of compound **21**

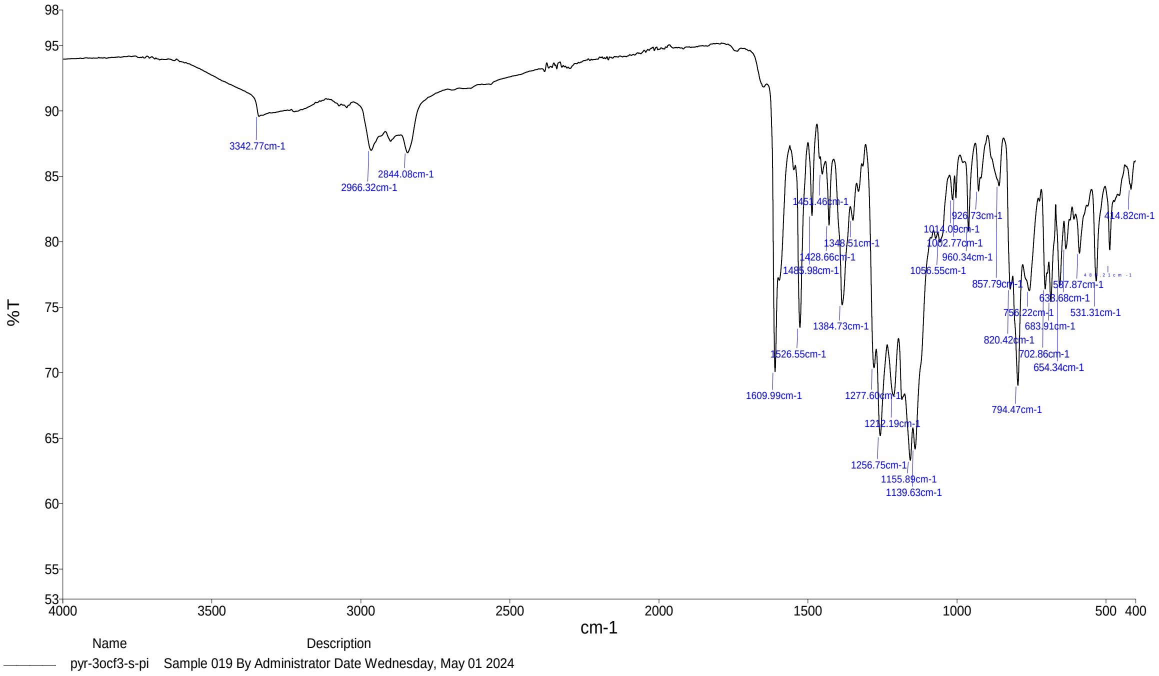

Figure S33. FTIR spectrum of compound **22**

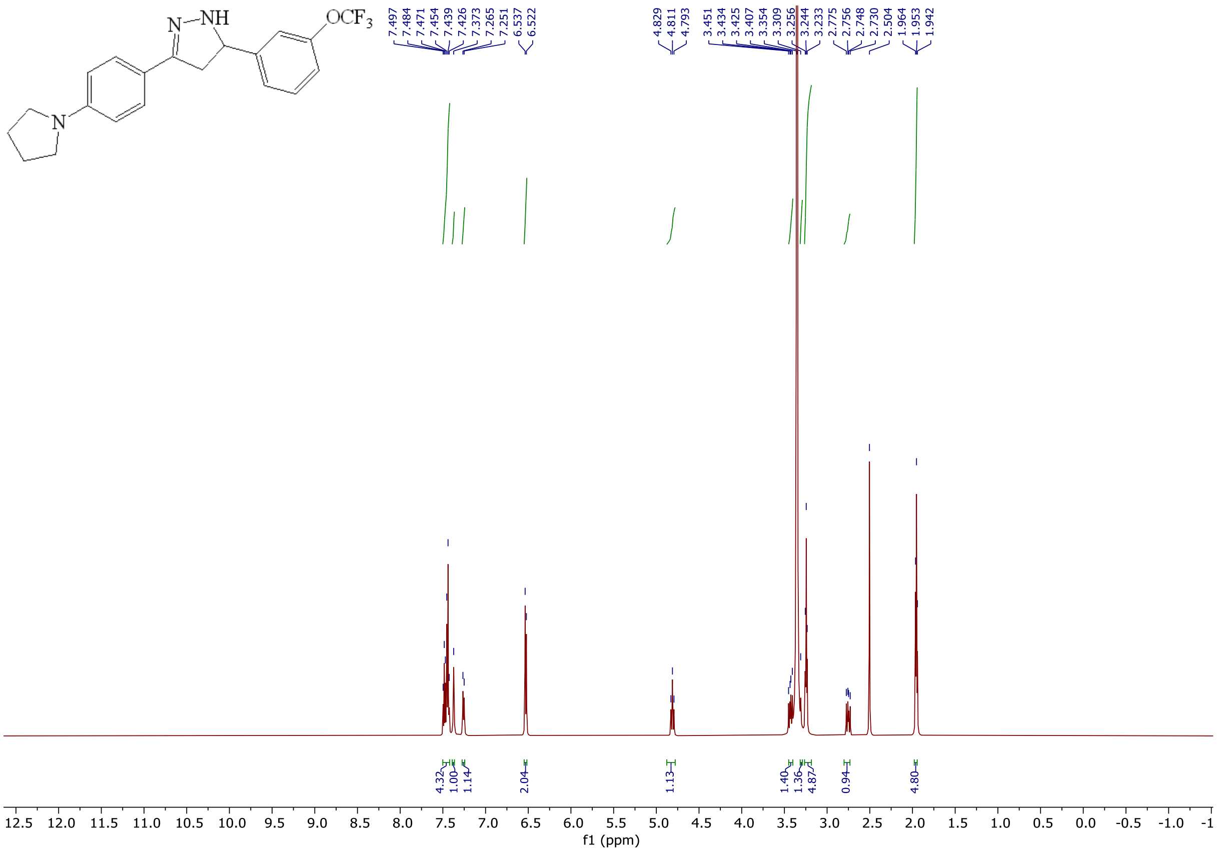

Figure S34. ^{1}H NMR spectrum of compound **22**

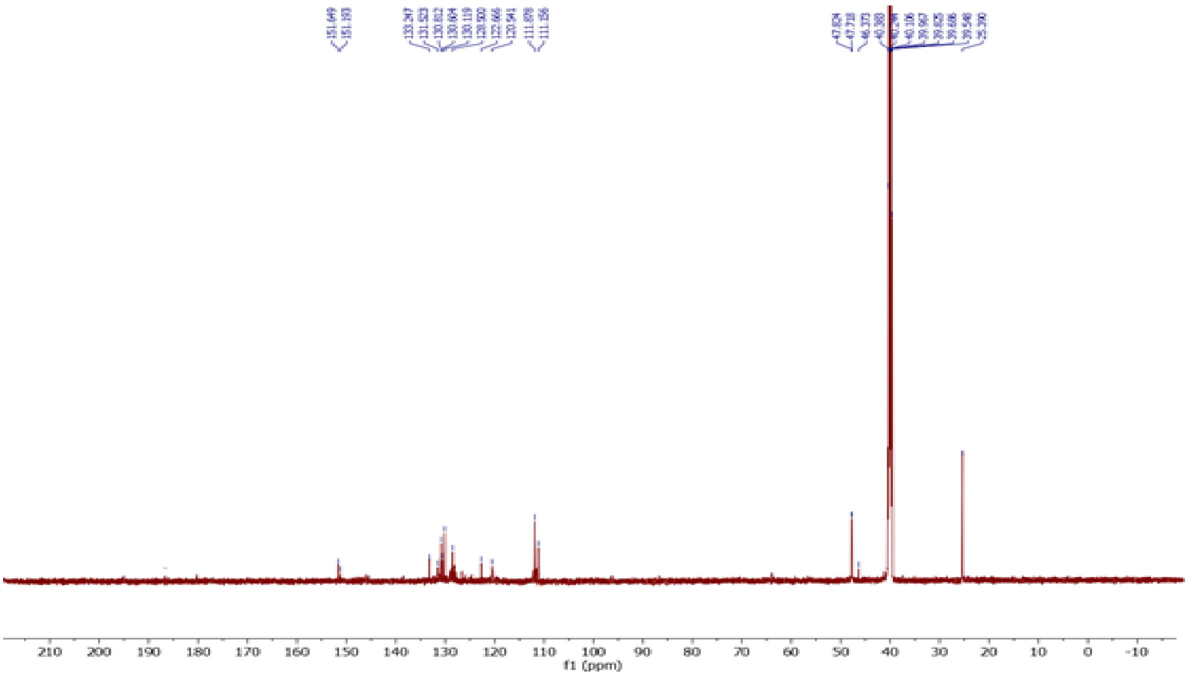

Figure S35. ^{13}C NMR spectrum of compound **22**

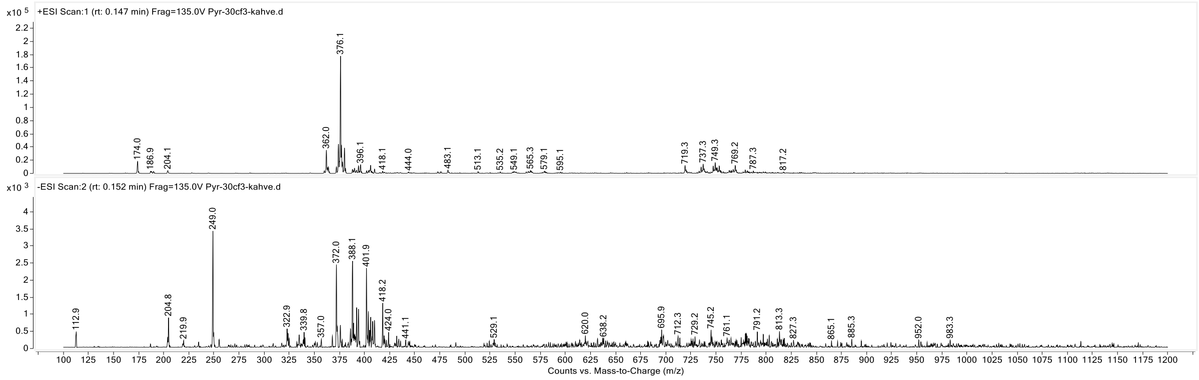

Figure S36. Mass spectrum of compound **22**

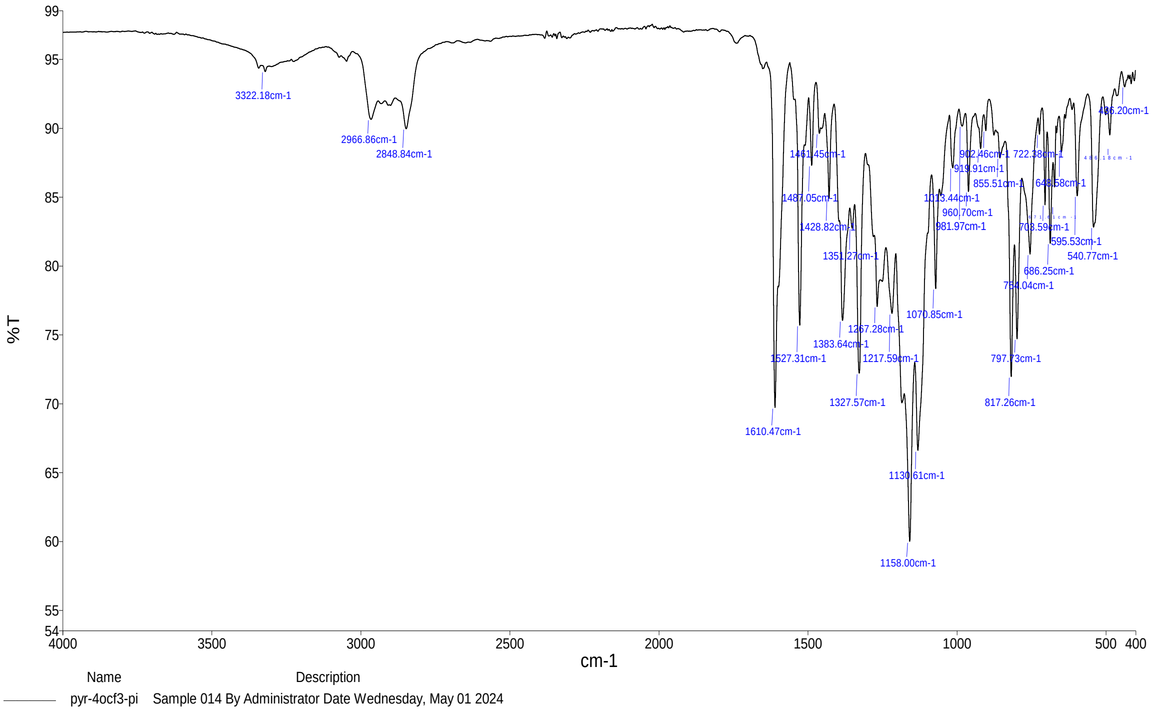

Figure S37. FTIR spectrum of compound **23**

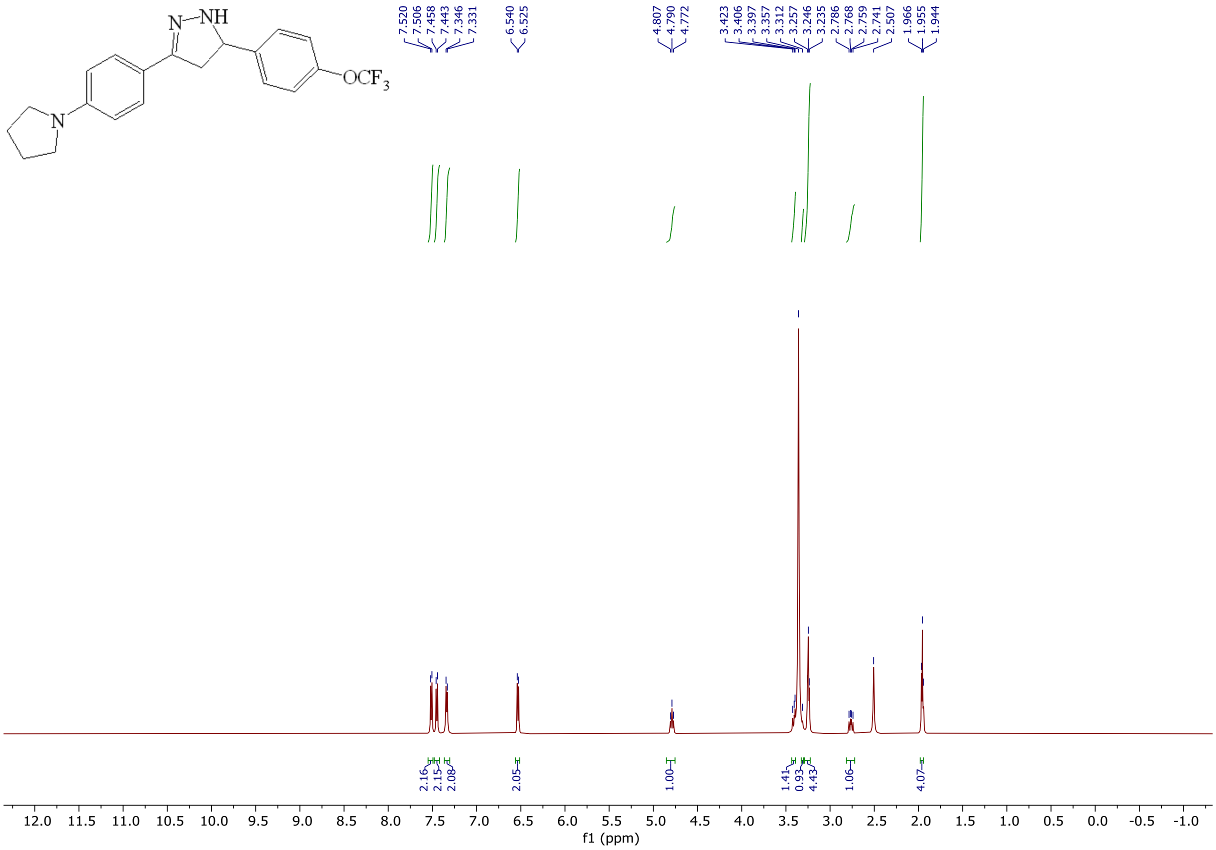

Figure S38. ^{1}H NMR spectrum of compound **23**

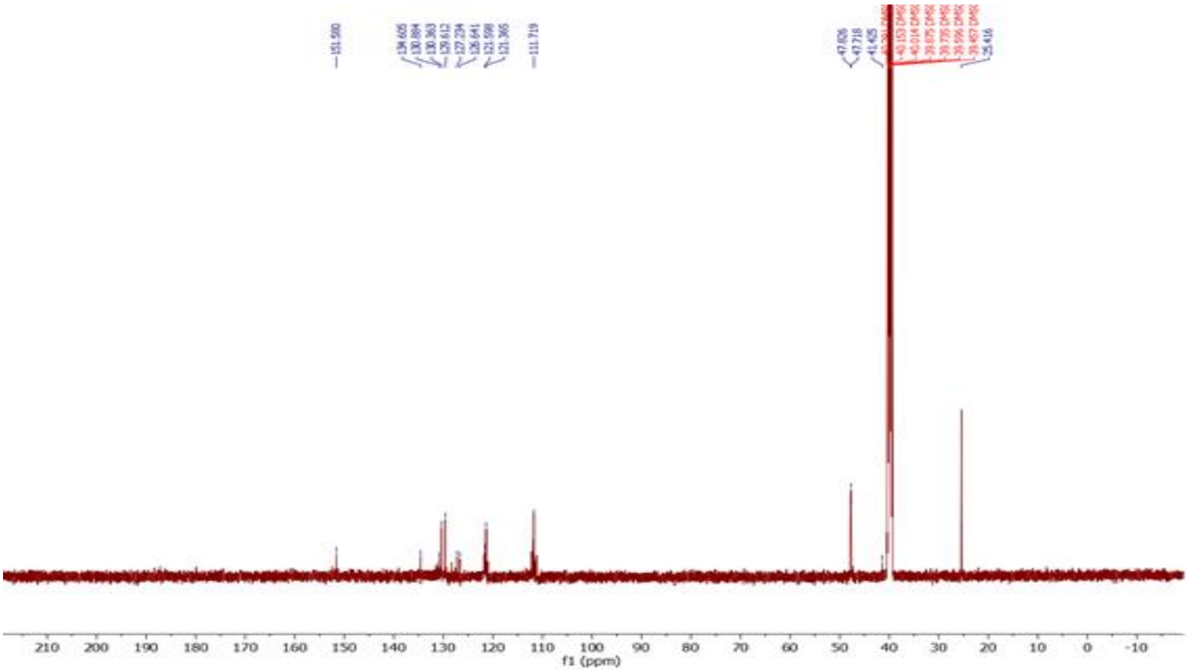

Figure S39. ^{13}C NMR spectrum of compound **23**

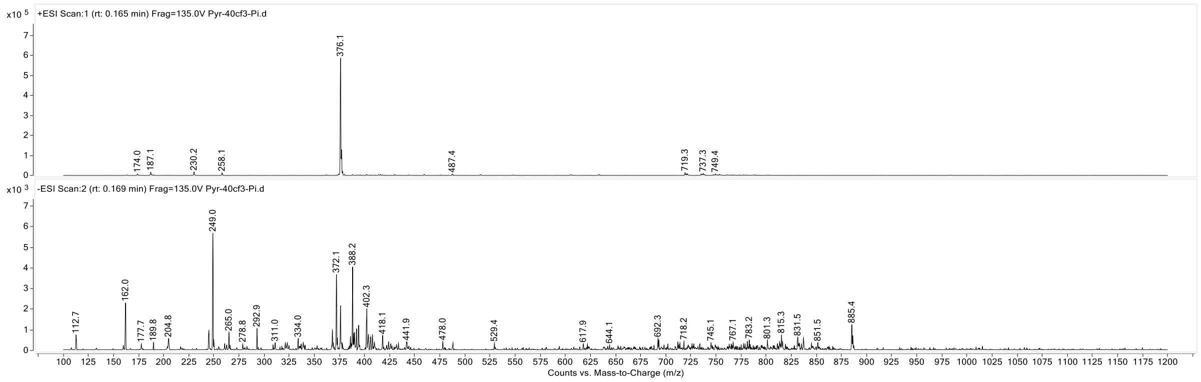

Figure S40. Mass spectrum of compound **23**

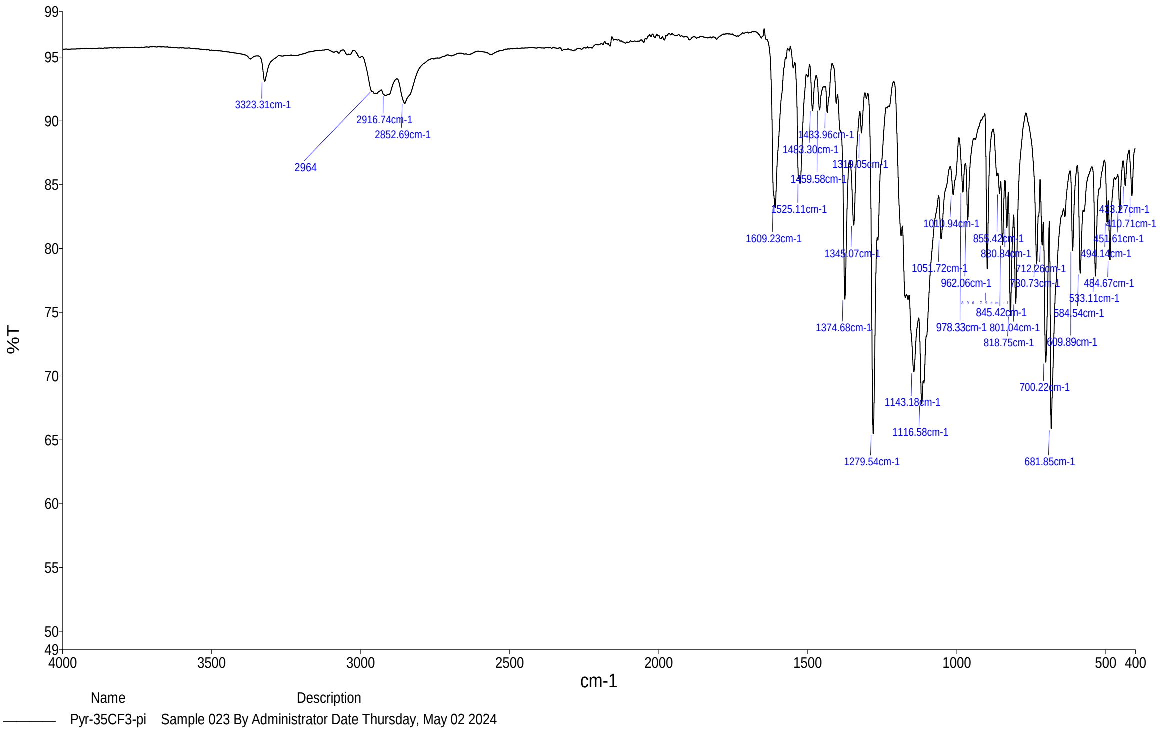

Figure S41. FTIR spectrum of compound **24**

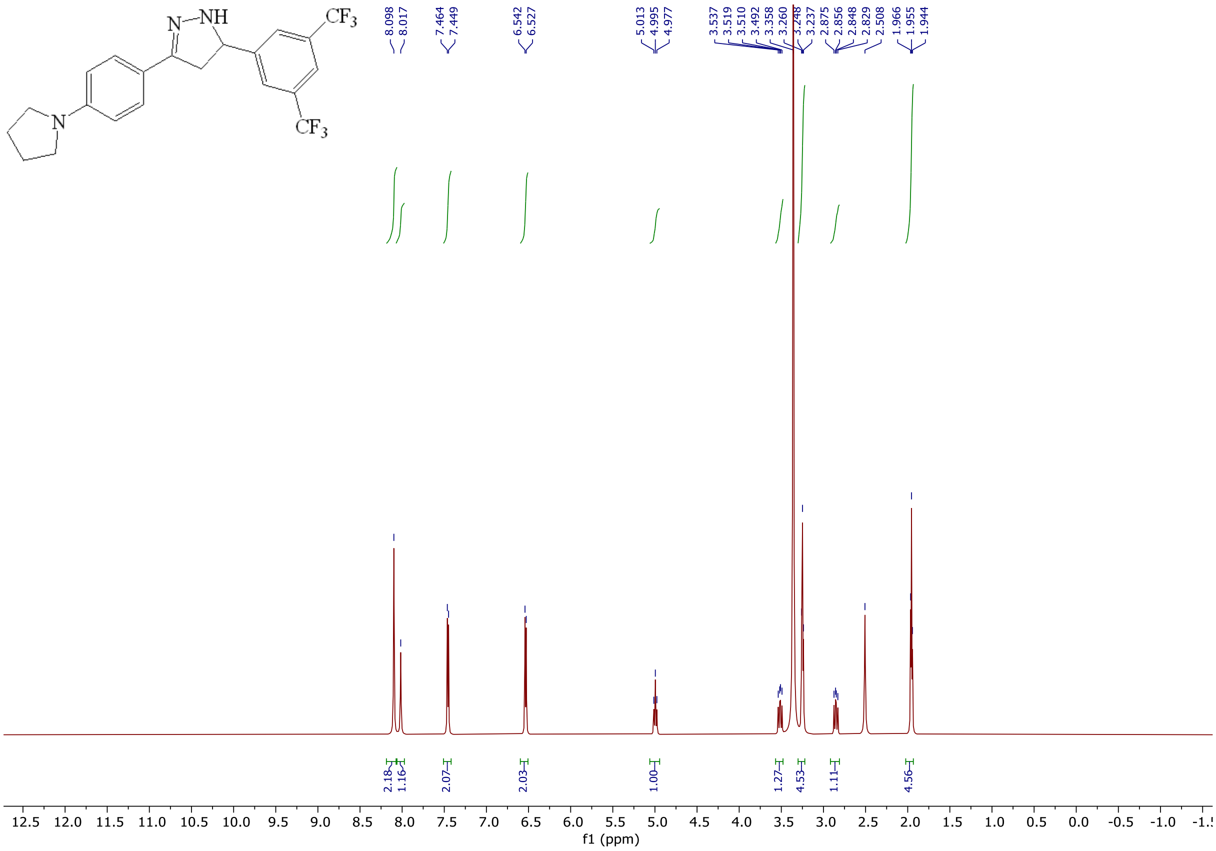

Figure S42. ^{1}H NMR spectrum of compound **24**

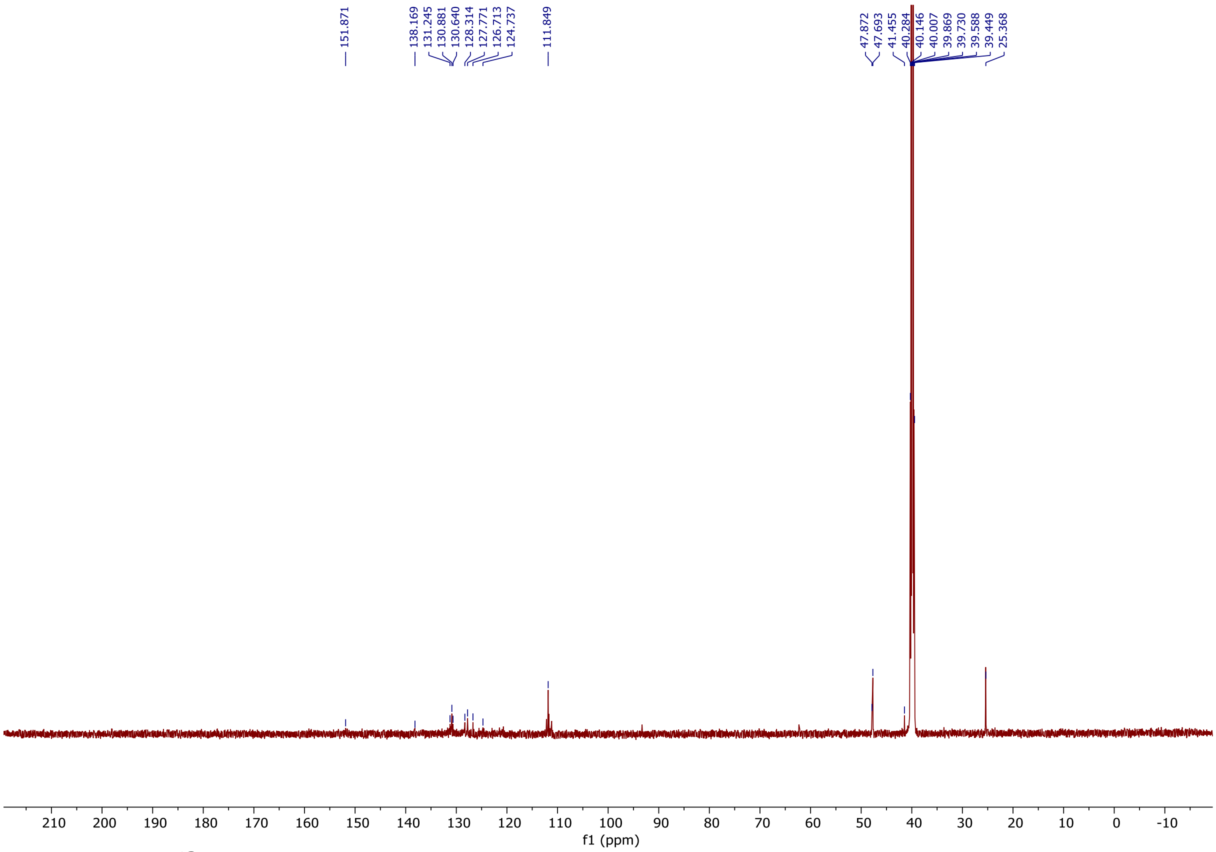

Figure S43. ^{13}C NMR spectrum of compound **24**

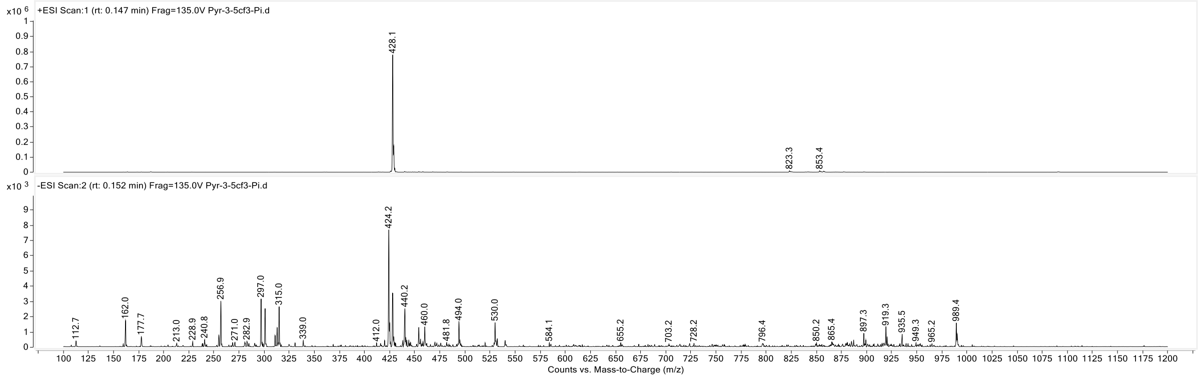

Figure S44. Mass spectrum of compound **24**

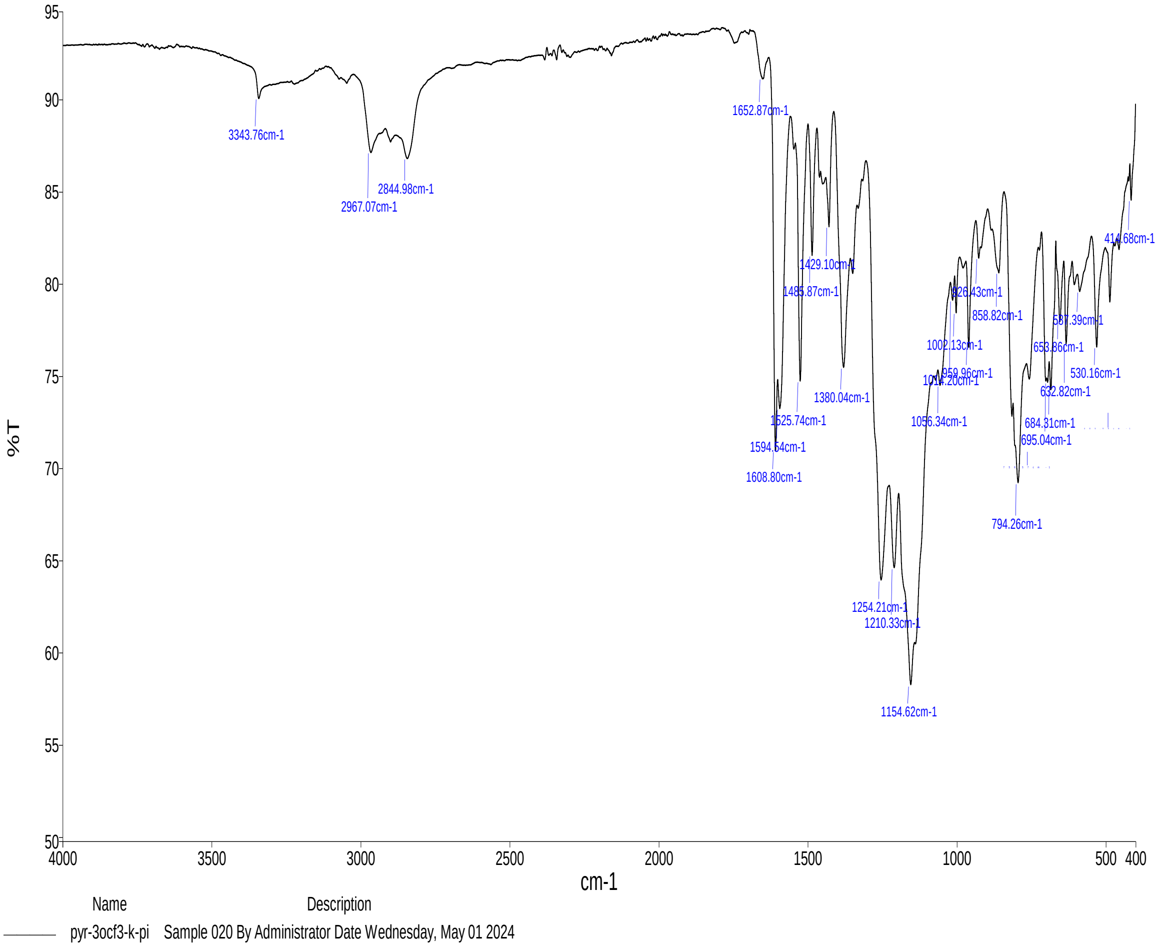

Figure S45. FTIR spectrum of compound **25**

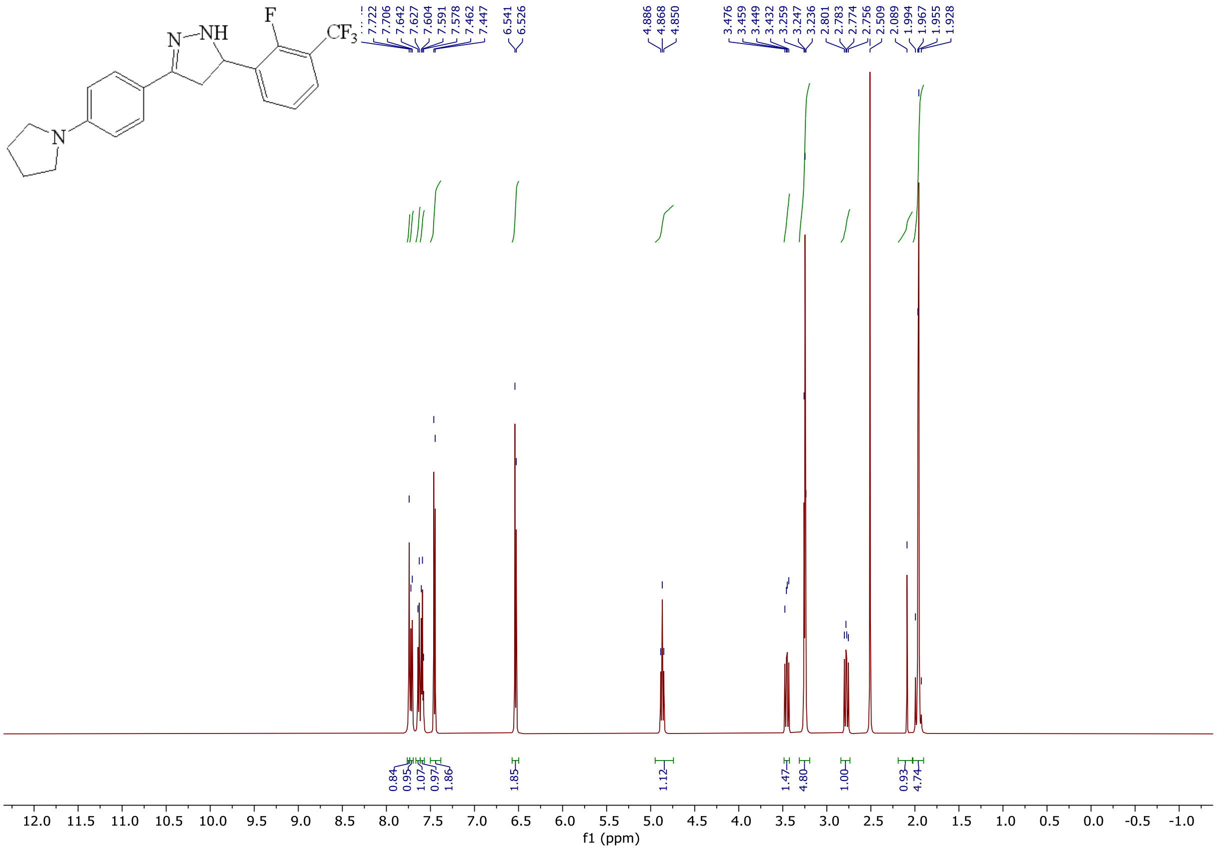

Figure S46. ^{1}H NMR spectrum of compound **25**

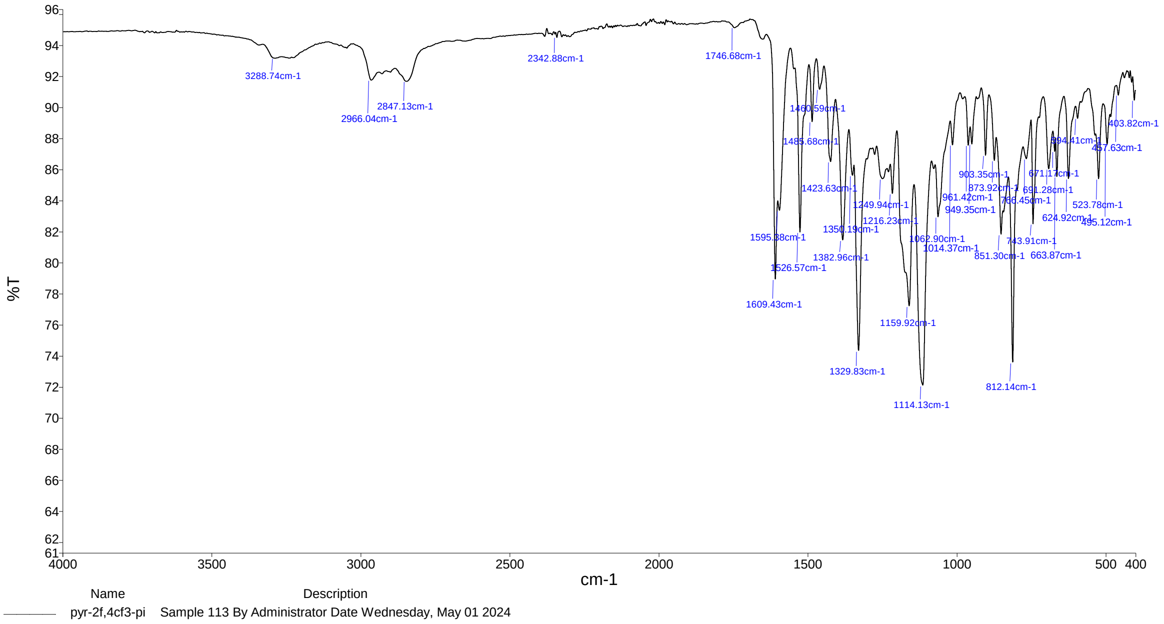

Figure S47. FTIR spectrum of compound **26**

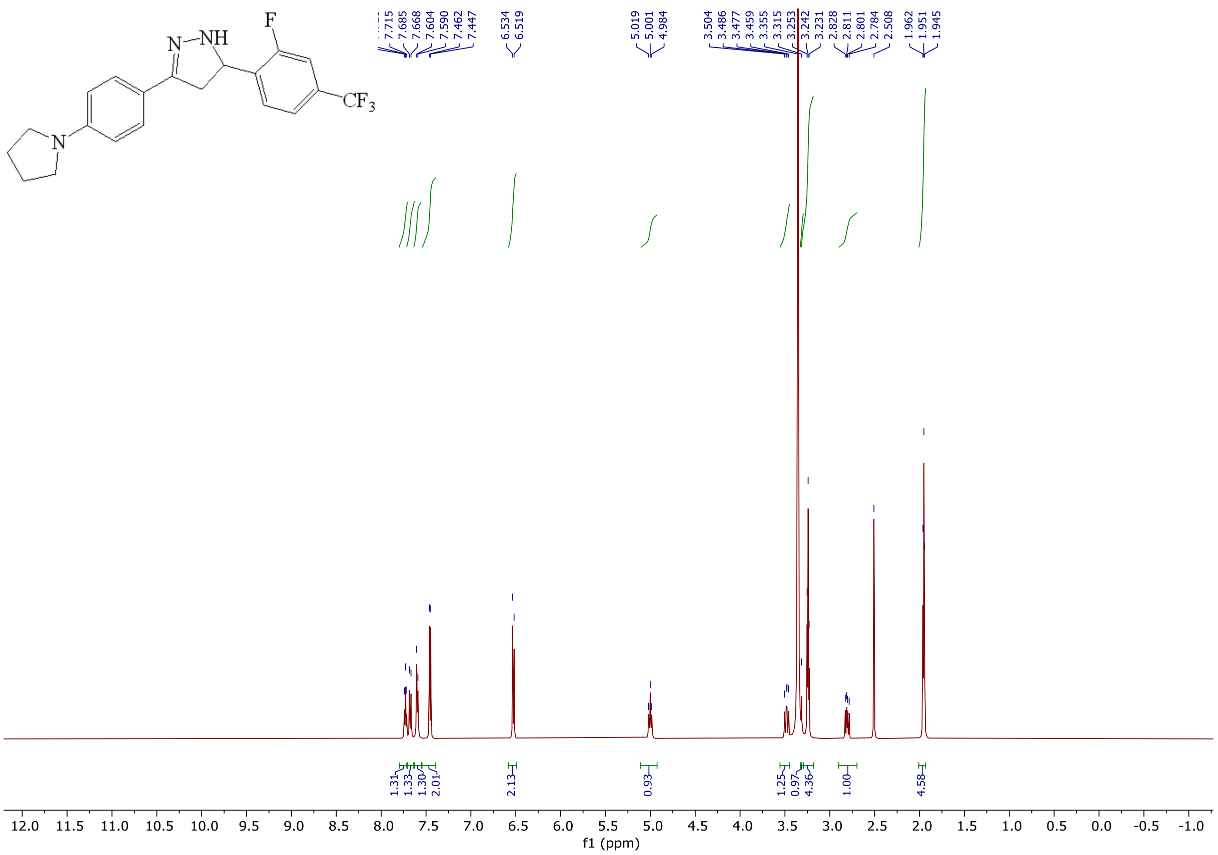

Figure S48. ^{1}H NMR spectrum of compound **26**

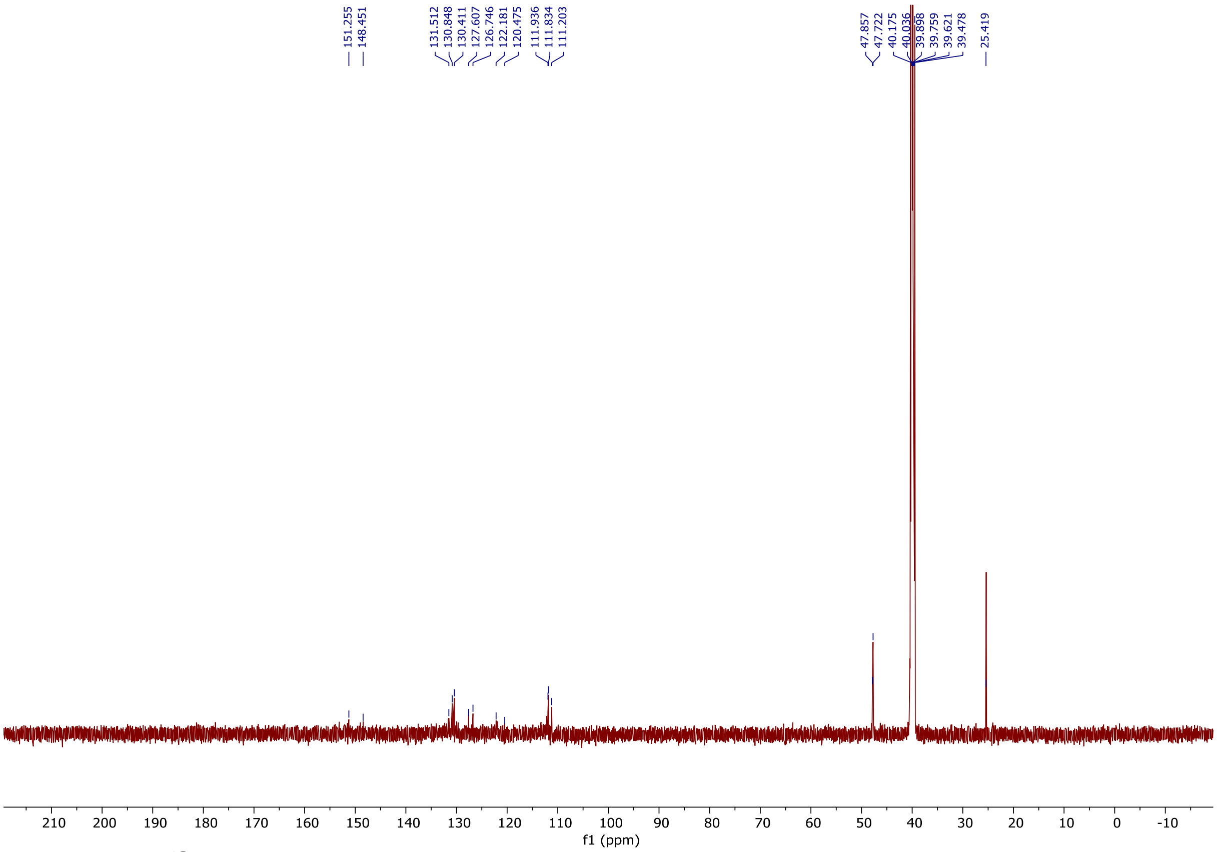

Figure S49. ^{13}C NMR spectrum of compound **26**

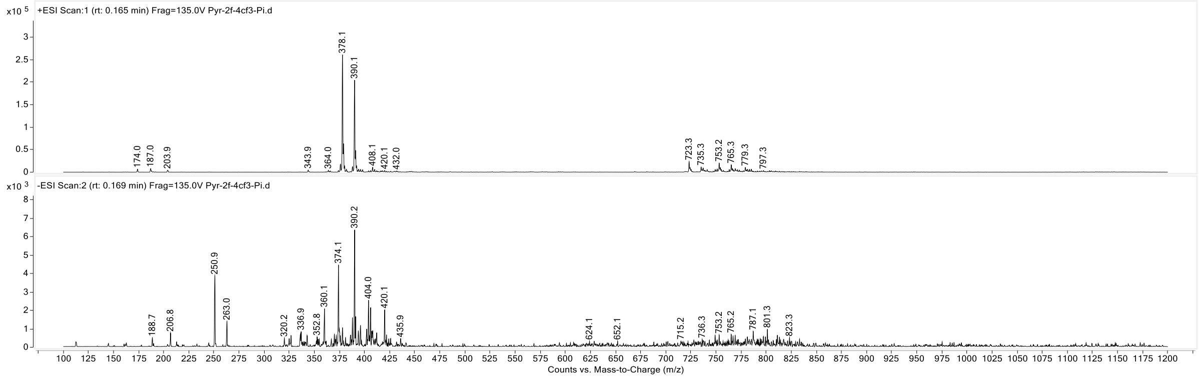

Figure S50. Mass spectrum of compound **26**

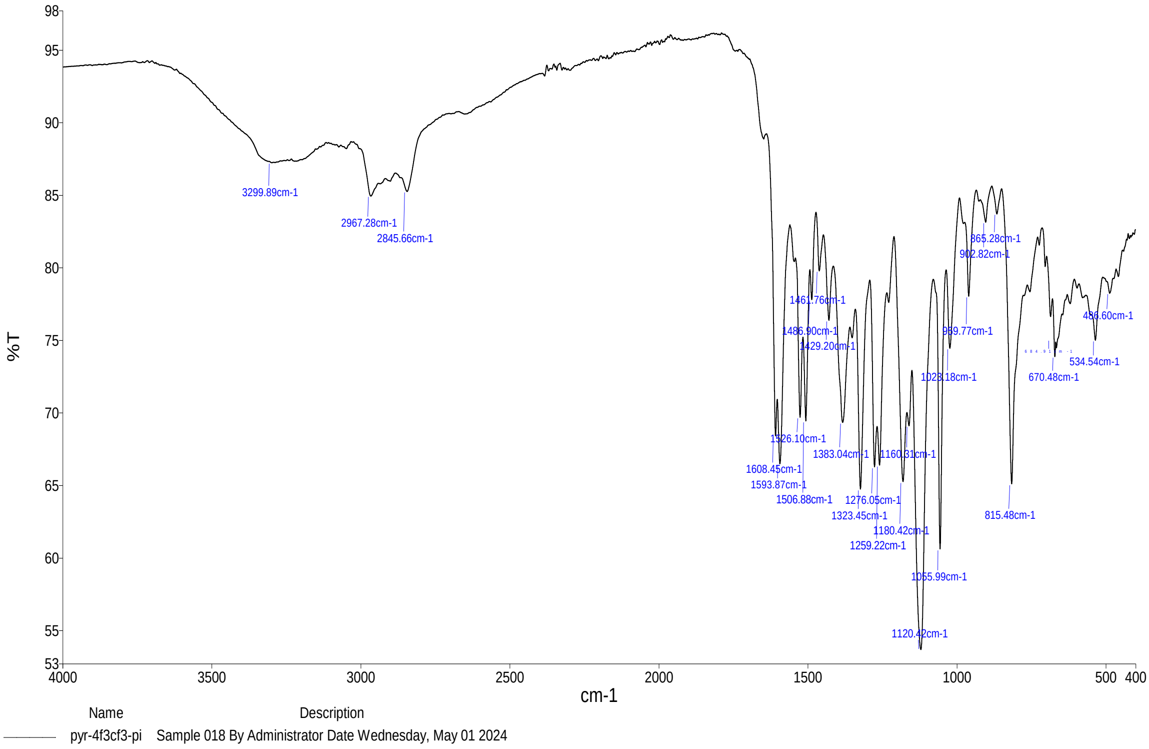

Figure S51. FTIR spectrum of compound **27**

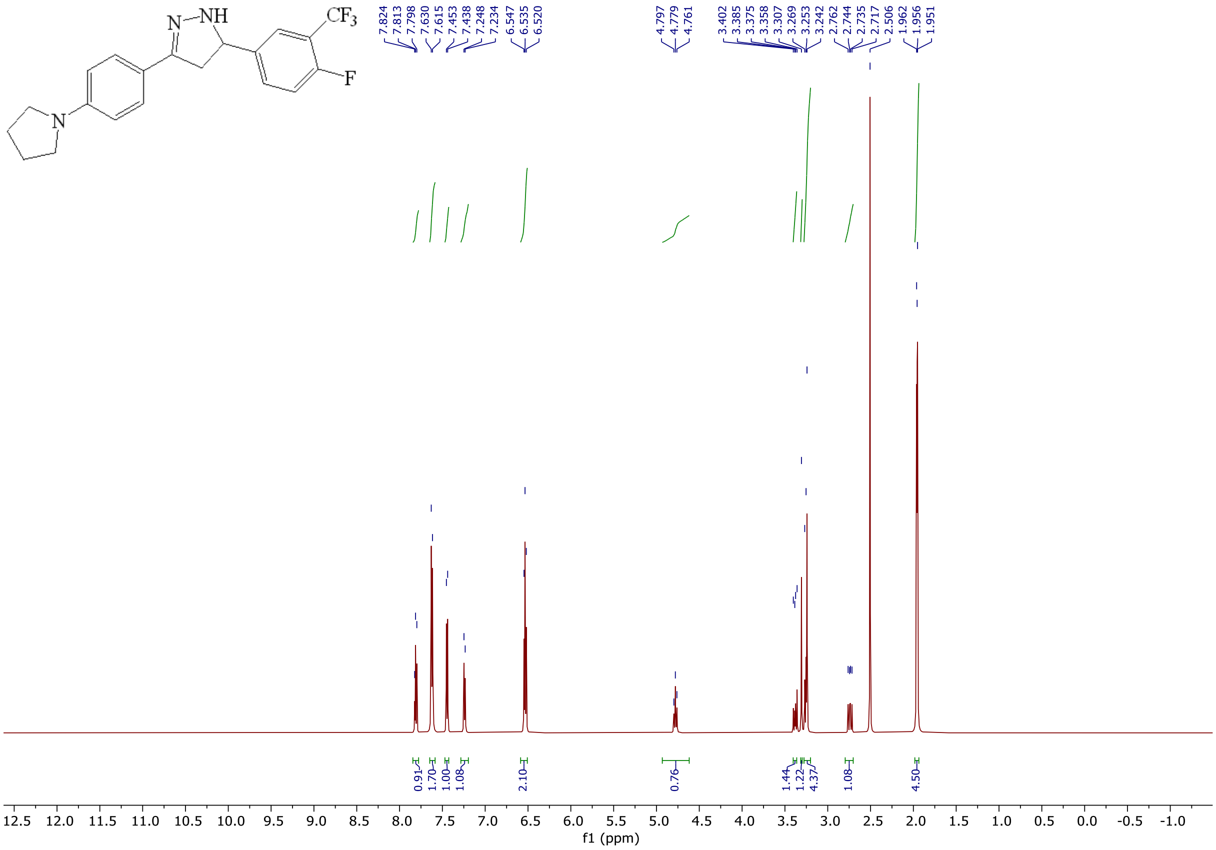

Figure S52. ^{1}H NMR spectrum of compound **27**

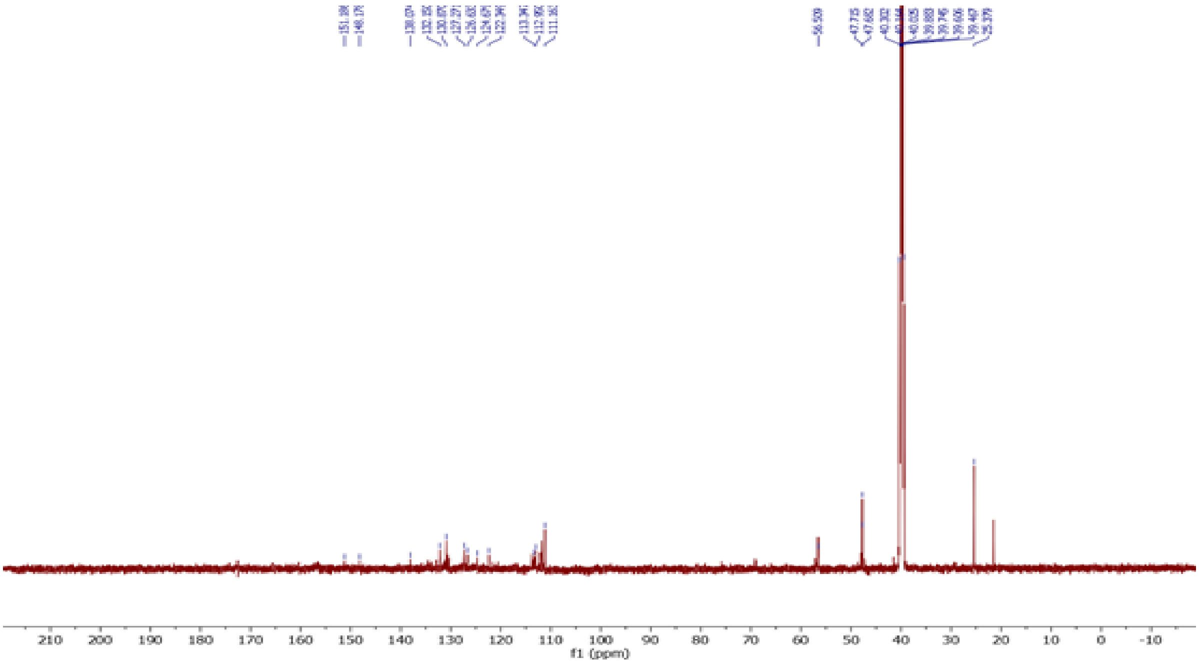

Figure S53. ^{13}C NMR spectrum of compound **27**

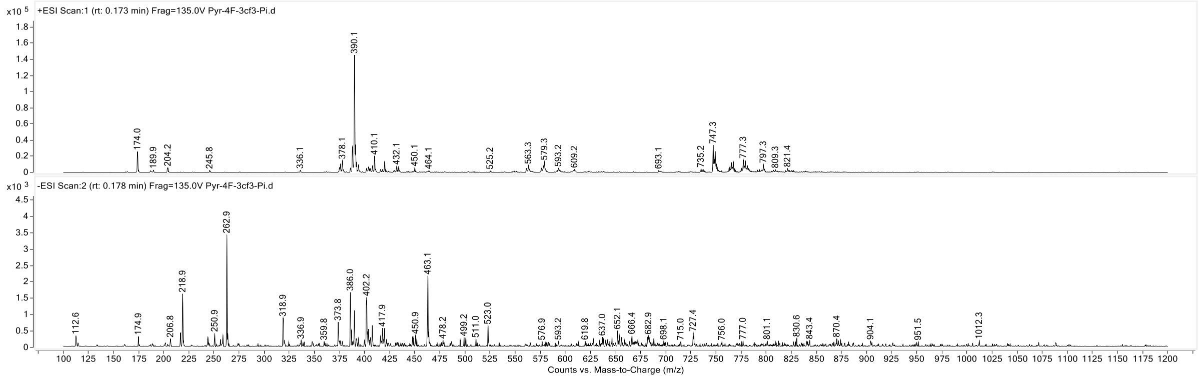

Figure S54. Mass spectrum of compound **27**
